# Bioactive Bioxanthracene and Cyclodepsipeptides from the Entomopathogenic Fungus *Blackwellomyces roseostromatus* BCC56290

**DOI:** 10.3390/antibiotics13070585

**Published:** 2024-06-24

**Authors:** Kunthida Phutthacharoen, Natalia A. Llanos-López, Rita Toshe, Wasana Noisripoom, Artit Khonsanit, Janet Jennifer Luangsa-ard, Kevin D. Hyde, Sherif S. Ebada, Marc Stadler

**Affiliations:** 1 Department of Microbial Drugs, Helmholtz Centre for Infection Research GmbH (HZI), Inhoffenstraße 7, 38124 Braunschweig, Germany; 6071105503@lamduan.mfu.ac.th (K.P.); natalia.llanos@helmholtz-hzi.de (N.A.L.-L.); ritatoshe840@gmail.com (R.T.); 2 Center of Excellence in Fungal Research, Mae Fah Luang University, Chiang Rai 57100, Thailand; kdhyde3@gmail.com; 3 Institute of Microbiology, Technische Universität Braunschweig, Spielmannstraße 7, 38106 Braunschweig, Germany; 4 National Center for Genetic Engineering and Biotechnology (BIOTEC), National Science and Technology Development Agency (NSTDA), 113 Thailand Science Park, Phahonyothin Rd., Khlong Nueng, Khlong Luang, Pathum Thani 12120, Thailand; wasana.noi@biotec.or.th (W.N.); artit.kho@biotec.or.th (A.K.); jajen@biotec.or.th (J.J.L.-a.); 5 School of Science, Mae Fah Luang University, Chiang Rai 57100, Thailand; 6 Department of Pharmacognosy, Faculty of Pharmacy, Ain Shams University, Cairo 11566, Egypt

**Keywords:** insect pathogen, Hypocreales, Ascomycota, beauveriolides

## Abstract

In the course of our ongoing research targeting the identification of potential biocontrol agents from entomopathogenic fungi (EPF), we explored a solid-state rice fungal extract of *Blackwellomyces roseostromatus* BCC56290 derived from infected lepidopteran larvae. Chemical and biological prospections afforded four unprecedentedly reported natural products differentiated into a dimeric naphthopyran bioxanthracene ES-242 derivative (**1**) and three cyclodepsipeptides (**2**–**4**) in addition to two known cyclodepsipeptides, cardinalisamides B (**5**) and C (**6**). Chemical structures of the isolated compounds were elucidated through comprehensive 1D/2D NMR and HR-ESI-MS data together with comparisons to the reported literature. The absolute configuration of the isolated cyclodepsipeptides was determined using Marfey’s method. All isolated compounds were assessed for their antimicrobial, cytotoxic, and nematicidal activities with some compounds revealing significant activities.

## 1. Introduction

Entomopathogenic fungi constitute a subgroup of soil-dwelling trophic fungi, infecting insects, majorly classified under the order Hypocreales, class Sordariomycetes, phylum Ascomycota [1]. The Hypocrealean Entomopathogenic Fungi (HEPF) comprise several genera including *Beauveria*, *Cordyceps,* and *Isaria* (family Cordycipitaceae) in addition to *Metarhizium* (family Clavicipitaceae) [2]. Species of *Beauveria* and *Metarhizium* serve as the most commercially applied biocontrol agents (BCAs) [3]. In addition, the HEPF revealed an immanent capacity to produce secondary metabolites (SMs) of diverse structural and pharmacological features including some marketed pharmaceuticals and/or agrochemicals [2,4,5]. The diversity in the HEPF parvome of SMs, ranging from molecules below 200 Da up to cyclic polypeptide (>1200 Da) [1], was reflected in a broad range of bioactivities mitigating the pathologic stresses affecting the hepatic, cardiovascular, immune, and nervous systems [2,4,5]. Among the other genera of the family Cordycipitaceae is the genus *Blackwellomyces* whose species were reported to infect the larvae of coleopteran and lepidopteran insects in particular *B. calendulinus* and *B. minutus* [6,7,8]. To the best of our knowledge, few SMs were reported from the genus *Blackwellomyces cardinalis* including the antitrypanosomal cyclohexadepsipeptides, cardinalisamides A–C [9], and oosporein, a bibenzoquinone derivative with potent antimicrobial, antifungal and insecticidal activities [10] whose biosynthetic gene cluster was recently described [11].

Along the course of our ongoing research targeting anti-infective secondary metabolites from HEPF, we came across the fungal strain *Blackwellomyces roseostromatus* BCC56290 derived from a soil-buried lepidopteran larva in Thailand. The current study reports the chemical and biological characterization of fungal secondary metabolites purified from its solid-state rice culture extract.

## 2. Results and Discussion

### 2.1. Isolation and Identification of ***1***–***6***

**Figure 1:**
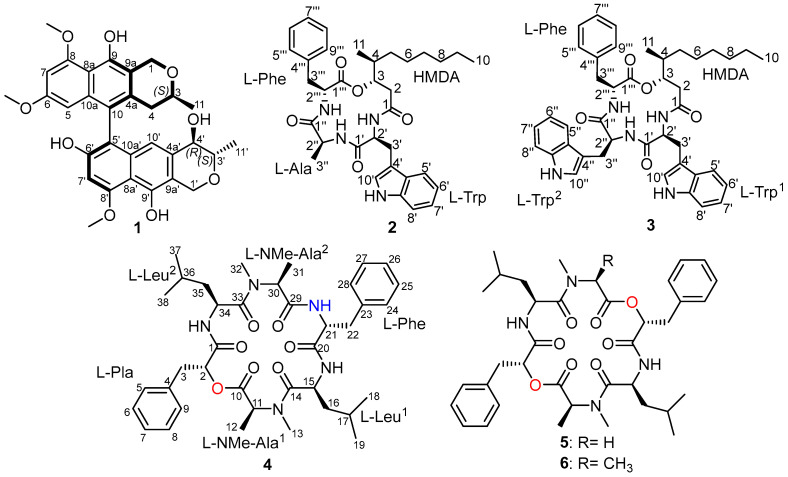
Chemical structures of **1**–**6**.

The solid-state rice culture of *B. roseostromatus* was subjected to chromatographic workup schemes starting with vacuum liquid chromatography followed by preparative HPLC isolations of the promising fractions. These procedures afforded four previously undescribed natural products (**1**–**4**) and two known cyclohexdepsipeptides (**5** and **6**) (Figure 1).

**Figure 2:**
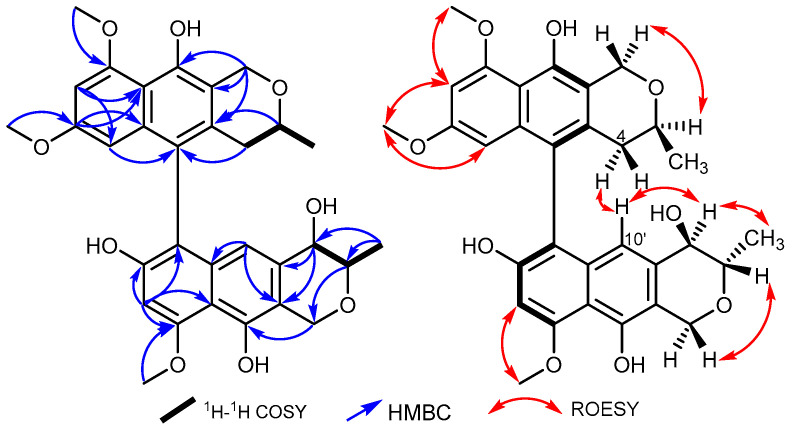
Key ^1^H–^1^H COSY, HMBC and ROESY correlations of **1**.

Compound **1** was obtained as a pale yellow amorphous solid. Its molecular formula was established as C_31_H_32_O_9_ based on the acquired HR-ESI-MS spectrum that revealed a protonated molecular ion peak at *m*/*z* 549.2110 [M + H]^+^ (calculated 549.2119) and a sodium adduct peak at *m*/*z* 571.1939 [M + Na]^+^ (calculated 571.1939) and hence indicating sixteen degrees of unsaturation. The ^13^C NMR spectral data and the HSQC spectrum of **1** (Table 1, Figure S7) displayed the presence of thirty-one carbon resonances with twenty of them recognized as sp^2^ differentiated into sixteen unprotonated and three methine carbon atoms. The assigned twenty sp^2^ carbon atoms accounted for ten degrees of unsaturation, thus indicating that compound **1** comprises six rings in its structure. A literature review of **1** suggested its chemical structure to be an analog of ES-242s, a group of bioxanthracene derivatives previously reported from fungal strains of the two genera *Verticillium* [12,13] and *Cordyceps* [14,15] with a novel *N*-methyl-D-aspartate (NMDA) receptor antagonistic activity [16]. The ^1^H–^1^H COSY spectrum of **1** (Figure 2) revealed the presence of two different spin systems. The first spin system extends from one aliphatic oxygenated methine proton at *δ*_H_ 3.64 (m, H-3) to a doublet methyl group at *δ*_H_ 1.06 (d, *J* = 6.2, H_3_-11) together with two diastereotopic methylene proton signals at *δ*_H_ 1.96 (dd, *J* = 16.7, 10.9 Hz, H-4α) and 2.20 (dd, *J* = 16.7, 3.0 Hz, H-4β) which were correlated via the HSQC spectrum to one sp^3^ secondary carbon atom at *δ*_C_ 34.2 (C-4). The second spin system was recognized between two oxygenated methine proton signals at *δ*_H_ 3.99 (t, *J* = 8.4 Hz, H-4′) and *δ*_H_ 3.31 (overlapped, H-3′) and extending to a doublet methyl group at *δ*_H_ 1.18 (d, *J* = 6.0 Hz, H_3_-11′). In addition, the ^1^H–^1^H COSY spectrum of **1** (Figure 2) revealed a long-range ^4^*J* correlation between two *meta*-coupled aromatic protons at *δ*_H_ 6.04 (d, *J* = 2.2 Hz, H-5) and 6.56 (d, *J* = 2.2 Hz, H-7). The HMBC and HSQC spectra of **1** (Figure 2, Figures S6 and S7) revealed the presence of three singlet methoxy groups at *δ*_H_ 3.46 (6-OCH_3_; *δ*_C_ 54.9), 4.04 (8-OCH_3_; *δ*_C_ 56.5) and 4.05 (8′-OCH_3_; *δ*_C_ 56.1) that revealed clear HMBC correlations to three oxygenated aromatic carbon atoms at *δ*_C_ 156.7, 157.3 and 156.2 ascribed to C-6, C-8 and C-8′, respectively. The relative stereochemistry of **1** was deduced through its ROESY spectrum (Figure 2) that revealed clear ROE correlations from H-3 to the pseudo-axial proton H-1α at *δ*_H_ 4.66 (d, *J* = 15.4 Hz) and from H_3_-11′ to the pseudo-axial proton H-4′ at *δ*_H_ 3.99 (t, *J* = 8.4 Hz) indicating cofacial orientations of each pair. In addition, the ROESY spectrum (Figure 2) revealed key correlations from H-7′ to 8′-OCH_3_ and from H-7 to both 6-OCH_3_ and 8-OCH_3_ confirming the chemical structure of **1** as an ES-242 derivative.

According to the obtained results, the axial chirality of **1** was suggested to be either a 6′-*O*-desmethyl derivative of ES-242-8 [14] or 4-deoxy-6′-*O*-desmethyl derivative of its atropisomer [15]. To distinguish between the two suggested atropisomers, the ROE correlation from H_2_-4 to H-10′ was examined and an intense ROE cross-peak was noticed from the pseudoaxial proton H-4α to H-10′ indicating the axial chirality at the C-10/C-5′ to be as depicted in **1** (Figure 1). Based on the obtained results, compound **1** was identified as a previously undescribed naphthopyran bioxanthracene derivative that was named ES-242-9.

**Figure 3:**
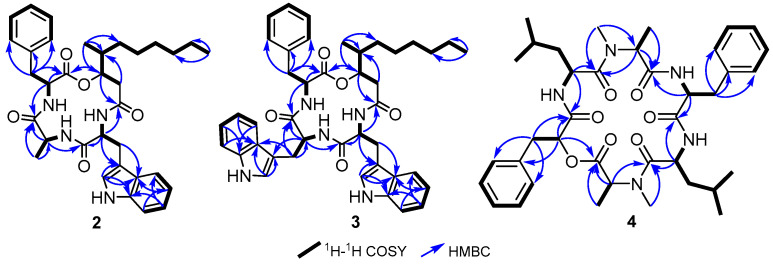
Key ^1^H–^1^H COSY and HMBC correlations of **2–4**.

Compound **2** was isolated as a yellowish-white amorphous solid. The HR-ESI-MS spectrum of **2** revealed a protonated molecular ion peak and a sodium adduct at *m*/*z* 589.3388 [M + H]^+^ (calculated 589.3384) and 611.3207 [M + Na]^+^ (calculated 611.3204), respectively. Thus, its molecular formula was established as C_34_H_44_N_4_O_5_ indicating fifteen degrees of unsaturation. The ^1^H NMR spectral data of **2** in DMSO-*d*_6_ (Table 2, Figure S11) revealed characteristic features of a peptide through the presence of three exchangeable amide NH signals (δ_H_ 7.31–8.51) together with three amino acid *α*-proton signals (δ_H_ 3.82–4.62) and two diastereotopic *β*-methylene groups (δ_H_ 3.02/3.12, 2.86/2.92). In addition, the ^1^H NMR spectral data of **2** also revealed one deshielded pyrrole NH proton at δ_H_ 10.86 (d, *J* = 2.4 Hz) and two doublet methyl groups at δ_H_ 1.11 (d, *J* = 6.9 Hz) and 0.75 (d, *J* = 6.9 Hz). The ^1^H–^1^H COSY spectrum revealed a spin system extending over four aromatic protons at δ_H_ 7.51 (H-5′)/6.97 (H-6′)/7.06 (H-7′)/7.32 (H-8′), a second spin system over three proton signals at δ_H_ 7.19 (H-5‴,9‴)/7.21 (H-6‴,8‴)/7.26 (H-7‴) integrated for five protons in addition to a spin system from one *α*-proton at δ_H_ 3.82 (p, *J* = 6.9 Hz, H-2″) to a doublet methyl group at δ_H_ 1.11 (d, *J* = 6.9 Hz, H_3_-3″) and an amide NH at δ_H_ 8.47 (d, *J* = 7.3 Hz). These structural features indicated that **2** is a peptide comprising three amino acids suggested to be tryptophan (Trp), alanine (Ala), and phenylalanine (Phe). In addition, the ^1^H–^1^H COSY spectrum revealed an additional spin system extending from a diastereotopic methylene group at δ_H_ 2.34/2.46 (H_2_-2) over two methines protons at δ_H_ 4.83 (H-3) and δ_H_ 1.91 (H-4)/δ_H_ 0.75 (d, *J* = 6.9 Hz, H_3_-11) then five methylene groups ending by a triplet methyl group at δ_H_ 0.85 (t, *J* = 6.9 Hz, H_3_-10) indicating the presence of a 3-hydroxy-4-methyldecanoyl moiety (HMDA). A literature search of **2** revealed its structural similarity to beauveriolides, cyclic tetradepsipeptides previously reported from entomopathogenic fungi of the genera *Beauveria* [17,18,19], *Cordyceps* [20,21] and *Isaria fumosorosea* (formerly *Paecilomyces fumosoroseus*) [18,22]. By careful comparison with the reported literature [17,18,19,20,21,22] alongside the HMBC spectrum of **2** (Figure 3), its amino acid sequence was determined as HMDA-Trp-Ala-Phe based on the key correlations from H-3 to C-1 (δ_C_ 170.3)/C-1‴ (δ_C_ 168.7); from H-2′ at δ_H_ 4.21 (q, *J* = 7.6 Hz, *α*H-Trp) to C-1/C-1′ (δ_C_ 171.4); from H-2″ at δ_H_ 3.82 (p, *J* = 6.9 Hz, *α*H-Ala) to C-1′/C-1″ (δ_C_ 170.7); and from H-2‴ at δ_H_ 4.62 (dd, *J* = 9.1, 7.8 Hz, *α*H-Phe) to C-1″/C-1‴.

Compound **3** was purified as a yellowish-white amorphous solid. Its HR-ESI-MS revealed a protonated molecular ion peak at *m*/*z* 704.3806 [M + H]^+^ (calculated 704.3806 for C_42_H_50_N_5_O_5_^+^) and a sodium adduct at *m*/*z* 726.3627 [M + Na]^+^ (calculated 726.3626 for C_42_H_49_N_5_NaO_5_^+^). Therefore, the molecular formula of **3** was determined as C_42_H_49_N_5_O_5_ indicating twenty-one degrees of unsaturation exceeding those in **2** by six degrees. The ^1^H NMR and the ^1^H–^1^H COSY spectral data of **3** in DMSO-*d*_6_ (Table 2, Figure 3 and Figure S21) revealed comparable characteristic features for **2** apart from the emergence of proton signals ascribed to a second Trp residue such as a deshielded pyrrole NH proton at δ_H_ 10.69 (d, *J* = 2.4 Hz) that revealed a key cross-peak to an olefinic proton at δ_H_ 6.81 (d, *J* = 2.4 Hz, H-10″) in addition to the presence of a second spin system among four olefinic protons at δ_H_ 7.47 (H-5″)/6.95 (H-6″)/7.04 (H-7″)/7.32 (H-8″). By comparing the 1D (^1^H and ^13^C) NMR data and the key HMBC correlations of **2** and **3** (Table 2, Figure 3, Figures S14 and S22), compound **3** was found to have a second Trp residue replacing Ala in **2** and hence has the amino acid sequence as HMDA-Trp^1^-Trp^2^-Phe. As for **2**, the absolute configurations of amino acid residues were determined to be 3*S*,4*S*-HMDA-L-Trp^1^-L-Trp^2^-L-Phe based on the common biosynthetic origin of beauveriolides [20], and the results of Marefy’s method (Figures S45–S46). Based on the aforementioned results, compound **3** was recognized as a previously undescribed cyclic tetradepsipeptide and was named beauveriolide U.

Compound **4** was obtained as a yellowish-brown amorphous solid with its molecular formula established as C_38_H_53_N_5_O_7_ indicating fifteen degrees of unsaturation based on its HR-ESI-MS that revealed a protonated molecular ion peak at *m*/*z* 692.4023 [M + H]^+^ (calculated 692.4018) and a sodium adduct at *m*/*z* 714.3836 [M + Na]^+^ (calculated 714.3837). The 1D (^1^H/^13^C) NMR spectral data of **4** (Table 3, Figure S27) exhibited the characteristic features of a peptide including the presence of three exchangeable amide NH signals (δ_H_ 7.42–7.92) together with two *N*-methyl moieties at δ_H_ 3.12 (δ_C_ 37.6) and 3.16 (δ_C_ 36.5) in addition to five amino acid *α*-proton signals (δ_H_ 3.47–4.93), and four diastereotopic *β*-methylene groups (δ_H_ 3.19/3.34, 2.90/3.15, 1.57/1.64, 1.47/1.53). The ^1^H–^1^H COSY spectrum of **4** (Figure 3) revealed two comparable spin systems each extending over a set of three aromatic protons signals at δ_H_ 7.15/7.27/7.18 and at δ_H_ 7.28/7.20/7.21 with each having a total integration index of five suggesting the presence of two monosubstituted aromatic rings. In addition, the ^1^H–^1^H COSY spectrum of **4** (Figure 3) revealed two pairs of comparable spin systems, one pair represents two leucine residues by spin systems extending from *α*-proton signals (δ_H_ 4.93/4.81) to two diastereotopic methylene groups (δ_H_ 1.57/1.64 or δ_H_ 1.47/1.53) then to two methine protons (δ_H_ 1.58/1.45) and ending either by pair of two doublet methyl groups (δ_H_ 0.84–0.89). The second pair of comparable spin systems revealed clear COSY cross-peaks between two overlapping *α*-proton signals at δ_H_ 3.48 to two doublet methyl groups at δ_H_ 1.32/1.19 with each having a coupling constant of 7.0 Hz. Based on the obtained results and by searching the reported literature, compound **4** was found to be structurally related to cardinalisamide C (**6**) [9], a symmetric cyclohexadepsipeptide isolated from *Blackwellomyces cardinalis* (aka *C. cardinalis*) NBRC 103832, an entomopathogenic fungus derived from an infected lepidopteran larva. A careful comparison of the ^1^H/^13^C NMR and HR-ESI-MS spectral data of **4** and cardinalisamide C (**6**) revealed that compound **4** lacks symmetry in **6** due to the replacement of one oxygen atom by nitrogen resulting from having one phenyllactic acid (Pla) and one Phe residue in **4** instead of having two Pla residues in **6**. This structural difference led to the asymmetry of **4**. The amino acid sequence in **4** was determined by acquiring its HMBC spectrum (Figure 3 and Figure S30) that revealed key correlations from an oxygenated methine proton at δ_H_ 5.05 (dd, *J* = 10.5, 3.9 Hz, H-2) to two carbonyl carbon atoms at δ_C_ 168.0 (C-1) and 170.0 (C-10): from an *α*-proton at δ_H_ 4.93 (dt, *J* = 9.5, 7.0 Hz, H-15) to C-10 and C14 (δ_C_ 171.2); from H-21 and H-30 to C-20 (δ_C_ 169.8) and C-29 (δ_C_ 170.2); and from H-34 to C-29 and C-33 (δ_C_ 171.8). Accordingly, compound **4** was found to be a cyclohexadepsipeptide composed of Pla-NMe-Ala^1^-Leu^1^-Phe-NMe-Ala^2^-Leu^2^. According to the results of Marfey’s analysis (Figures S46–S48) and based on the taxonomic proximity with common biosynthetic origin, the absolute configurations of the amino acid residues in **4** were deduced to be all in L-configuration. In conclusion, compound **4** was determined to be a previously undescribed cyclohexadepsipeptide and it was given a trivial name cardinalisamide D.

Compounds **5** and **6** were isolated similarly as yellowish-brown amorphous solids with their molecular formulas determined as C_37_H_50_N_4_O_8_ and C_38_H_52_N_4_O_8_ through their HR-ESI-MS spectra (Figures S34 and S40). A literature search of **5**/**6** and by comparing their ^1^H/^13^C NMR spectra to the reported literature [9], they were identified as cardinalisamides B and C, respectively.

### 2.2. Biological Assays

**Figure 4:**
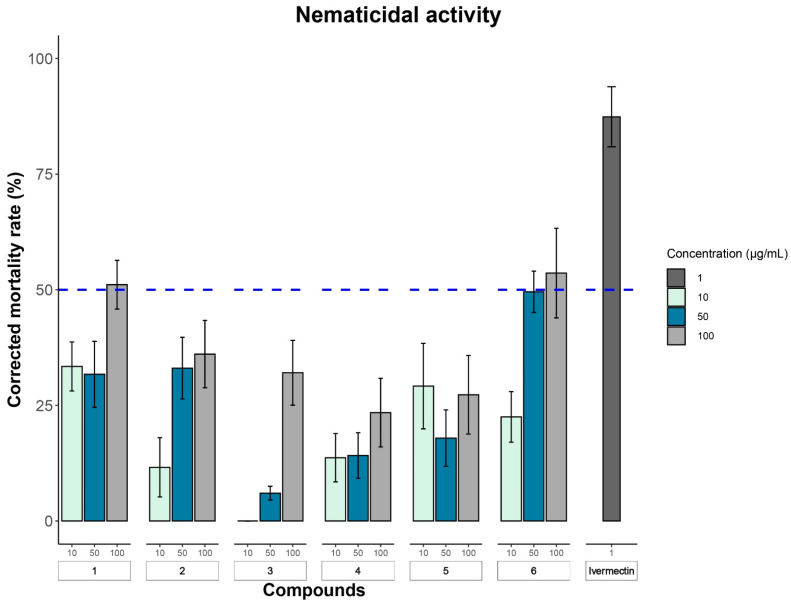
Bioassay of compounds **1**–**6** against *Caenorhabditis elegans*. Corrected mortality rate of compounds **1**–**6** (10 µg mL^−1^, 50 µg mL^−1^, and 100 µg mL^−1^) against *C. elegans* after 18 h of treatment. A solution of ivermectin (1 µg mL^−1^) was used as a positive control. The data were corrected using Schneider-Orelli’s formula based on the negative control as methanol and are shown as the mean ± SD (n ≥ 3). The blue dash line characterizes the IC_50_.

In our search for novel biological natural products with anti-infective activity, several bioassays were performed. All the isolated compounds **1**–**6** were assessed for their cytotoxic, antimicrobial, and nematicidal activity against a panel of different cell lines, Gram-positive/negative bacterial, fungal pathogens, and *Caenorhabditis elegans*, respectively. In cytotoxicity (MTT) assay, the obtained results (Table 4) revealed that among the tested compounds, only cardinalisamides B (**5**) and C (**6**) revealed significant pancytotoxic activities against almost all tested human cancer cell lines with IC_50_ values between 2.2 and 13.9 µM in spite of being relatively non-toxic against normal fibroblast (L929) cell line which gives a positive indication for their safety. The antimicrobial activity assay was conducted against a panel of twelve different bacterial and fungal pathogens (Table S1), however, none of the tested compounds proved to be active. According to the reported literature, related ES-242 derivatives revealed moderate to potent antimalarial activity against *Plasmodium falciparum* (K1, multidrug-resistant strain) at IC_50_ values between 3.3 and 12 µM [15]. Herein, the nematicidal activity assay results (Figure 4, Table S2) revealed that only ES-242-9 (**1**) and cardinalisamide C (**6**) exhibited significant effects against *C. elegans* with corrected mortality rates of 51.1 and 53.6% at 100 µg/mL, respectively. Being neither cytotoxic nor antimicrobial in the conducted assays, ES-242-9 (**1**) could be a suitable candidate for further assessment to develop a nematicidal agent. In the literature, cardinalisamides B (**5**) and (**6**) were first reported to have an almost equipotent in vitro antitrypanosomal activity against *Trypanosoma brucei*, with IC_50_ values of 12.8 and 12.5 μM, respectively [9].

The two previously undescribed beauveriolides T (**2**) and U (**3**) revealed no activity in any of the conducted assays. These cyclodepsipeptides belong to the class of beauveriolides, which are abundantly biosynthesized by HEPF [17,18,19]. Beauveriolides were first isolated from the insect pathogenic fungus *Beauveria bassiana* in 1977 [17]. Although various related derivatives have been reported to exhibit pharmacological properties, such as calmodulin (CaM) inhibition [23], and protective effects on HEI-OC1 cells [24], as well as stimulating glucose uptake in cultured rat L6 myoblasts [24], to the best of our knowledge, none has shown antimicrobial or nematicidal activities.

## 3. Materials and Methods

### 3.1. General Experimental Procedures

Optical rotation values were measured on a PerkinElmer 241 polarimeter at 20 °C (Anton-Paar Opto Tec GmbH, Seelze, Germany). UV spectra were acquired using a Shimadzu UV/VIS spectrophotometer UV-2450 (Shimadzu^®^, Kyoto, Japan). High-resolution electrospray ionization mass spectra (HR-ESI-MS) were measured on an Agilent 1200 Infinity Series HPLC-UV system (Agilent Technologies^®^, Santa Clara, CA, USA) equipped with a C_18_ Acquity UPLC BEH column (50 × 2.1 mm, 1.7 µm: Waters, Milford, MA, USA), solvent A: H_2_O + 0.1% formic acid (FA) (*v*/*v*); solvent B: acetonitrile (MeCN) + 0.1% FA (*v*/*v*), gradient: 5% B for 0.5 min increasing to 100% B in 19.5 min, holding at 100% B for 5 min, a flow rate of 0.6 mL min^−1^, UV/Vis detection 190–600 nm) connected to a Time-Of-Flight mass spectrometer (ESI-TOF-MS, Maxis, Bruker, Billerica, MA, USA) (scan range 100–2500 *m*/*z*, rate 2 Hz, capillary voltage 4500 V, dry temperature 200 °C). NMR spectra were recorded with an Avance III 500 spectrometer (Bruker^®^, Billerica, MA, USA, ^1^H-NMR: 500 MHz, and ^13^C-NMR: 125 MHz) dissolving compounds in deuterated DMSO-*d*_6_.

### 3.2. Fungal Material

*Blackwellomyces* sp. BCC56290 (Cordycipitaceae, Hypocreales, Sordariomycetes, Ascomycota) found on lepidopteran larva buried in soil in October 2012, by Artit Khonsanit, Kanoksri Tasanathai, Prasert Srikitikulchai, Rachada Promharn and Wasana Noisripoom in Chiang Mai Province, Kanlayaniwatthana District, Ban Chan Upriver Forest Nature Trail, located at coordinates 18°59′15″N and 98°17′09″E. The pure culture was deposited in the BIOTEC culture collection (BCC). Combined analyses of ITS, *LSU*, *EF1*, and *RPB1* sequences (GenBank accession numbers ITS = PP709052, *LSU* = PP711712, *EF1* = PP735441, and *RPB1* = PP735443) confirmed that *Blackwellomyces* sp. BCC56290 is nested within the type specimen of *B. roseostromatus* BCC91358 [7], confirming its identity (Maximum Likelihood phylogenetic tree inferred from 117 taxa of Cordycipitaceae based on combined ITS, *LSU*, *EF1* and *RPB1* sequence data, see Supplementary Figure S49). Fungal specimen was dried in a food dehydrator and deposited in the BIOTEC Bangkok Herbarium (BBH), Thailand Science Park, Pathum Thani Province, Thailand.

### 3.3. Fermentation, Extraction and Isolation

We evaluated the antiviral activities of *Blackwellomyces* species such as *B*. *aurantiacus*, *B*. *calendulinus*, *B*. *minutus*, and *B*. *roseostromatus*, against SARS-CoV-2 and CHIKV infections including the inhibitory effect on 3CLpro activity, cytotoxicity of the extracts to Vero E6, Huh7, and HEK293 cells used in the assays and found that *B*. *roseostromatus* BCC56290 extract inhibited CHIKV infection. We therefore chose this fungus due to our previous findings against the virus. Fermentation of the selected strain *B. roseostromatus* BCC56290 was grown on solid-state rice media (fermentations completed in 10 × 1000 mL Erlenmeyer flasks containing 180 g of rice in 180 mL distilled water) and inoculated with 10 pieces of fully grown 7 mm mycelial plugs. The rice cultures were incubated on static conditions in white light/dark cycles in the laboratory, under room temperature, until full growth of the mycelia was achieved (31 days from inoculation). Thereafter, the cultures were soaked overnight in acetone (500 mL) and extracted thrice under sonication. The solvent was evaporated to yield an aqueous phase (400 mL) that was extracted thrice with ethyl acetate in a ratio of 1:1 (*v*/*v*). The organic phase was filtered through anhydrous sodium sulfate and evaporated on a rotary evaporator to dryness. The extracts were transferred into vials and dried under nitrogen and thereafter their weights were determined.

The crude extract (4.43 g) was dissolved in 3.0 mL of MeOH and loaded on silica gel by trituration using mortar and pestle and then left to dry overnight. The loaded air-dried extract was applied on the top of a column dry-packed with silica gel. A vacuum liquid chromatography (VLC) procedure was initiated by applying a gradient elution starting with 100% *n*-hexane, *n*-hexane/EtOAc (7:3, 1:1, and 3:7) and 100% EtOAc. Then, the gradient was switched to 100% DCM, DCM/MeOH (9:1, 8:2, 1:1) and ended by washing with 100% MeOH (*v*/*v*) affording ten fractions (F1–F10).

Fraction 3 (F3, 179 mg, *n*-hexane/EtOAc (1:1)) was further purified using preparative reversed-phase liquid chromatography (PLC 2020, Gilson, Middleton, Wisconsin, USA) equipped with a Gemini C_18_ column (50 × 21 mm, 10 μm, Phenomenex, Aschaffenburg, Germany) as a stationary phase. Deionized water (Milli-Q, Millipore, Schwalbach, Germany) supplemented with 0.1% formic acid (FA) (solvent A) and acetonitrile (MeCN) with 0.1% FA (solvent B) were used as the mobile phase. The elution gradient used for fractionation started using 20% of solvent B (MeCN + 0.1% FA) and 80% of solvent A (deionized water + 0.1% FA) for 5 min. Then, the gradient continued from 20 to 40% of solvent B over 10 min and from 40 to 100% of solvent B over 30 min ending by holding 100% of solvent B for 10 min, the flow rate was set to 15 mL/min, and UV detection was carried out at 210, 225, 275, and 330 nm to yield **1** (1.6 mg, *t*_R_ = 11.0 min), **5** (2.0 mg, *t*_R_ = 18.0 min), and **6** (2.1 mg, *t*_R_ = 21.0 min).

Fractions 4 (F4, *n*-hexane/EtOAc (3:7)) and 5 (F5, 100% EtOAc) were pooled and concentrated under reduced pressure yielding 157 mg. Afterward, the combined fraction was subjected to preparative HPLC separations adopting the same conditions and gradient elution as for F3 to obtain **4** (4.1 mg, *t*_R_ = 17.0 min), **2** (1.5 mg, *t*_R_ = 19.0 min), and **3** (3.8 mg, *t*_R_ = 20.0 min).

Spectral data (see also Figures S1–S48)

ES-242-9 (**1**): Pale yellow amorphous solid; ${\left[\alpha \right]}_{D}^{20}$ +32.0° (*c* 0.1, acetone); UV/Vis (MeOH): λ_max_ (log *ε*) = 201 (4.91), 239 (4.91), 293.5 (4.07), 309 (4.04), 346 (3.95) nm; NMR data (^1^H NMR: 500 MHz, ^13^C NMR: 125 MHz in DMSO-*d*_6_) see Table 1; HR-(+)ESI-MS: *m*/*z* 549.2110 [M + H]^+^ (calcd. 549.2119 for C_31_H_33_O_9_^+^), 571.1939 [M + Na]^+^ (calcd. 571.1939 for C_31_H_32_NaO_9_^+^); *t*_R_ = 9.66 min (LC-ESI-MS). C_31_H_32_O_9_ (548.20 g/mol).

Beauveriolide T (**2**): Yellowish-white amorphous solid; ${\left[\alpha \right]}_{D}^{20}$ –66.7° (*c* 0.06, DMSO); UV/Vis (MeOH): λ_max_ (log *ε*) = 194 (4.21), 202 (4.44), 218.5 (4.43), 279.5 (3.71), 289.5 (3.63) nm; NMR data (^1^H NMR: 500 MHz, ^13^C NMR: 125 MHz in DMSO-*d*_6_) see Table 2; HR-(+)ESI-MS: *m*/*z* 589.3388 [M + H]^+^ (calcd. 589.3384 for C_34_H_45_N_4_O_5_^+^), 611.3207 [M + Na]^+^ (calcd. 611.3204 for C_34_H_44_N_4_NaO_5_^+^); *t*_R_ = 13.15 min (LC-ESI-MS). C_34_H_44_N_4_O_5_ (588.33 g/mol).

Beauveriolide U (**3**): Yellowish-white amorphous solid; ${\left[\alpha \right]}_{D}^{20}$ –51.0° (*c* 0.1, acetone); UV/Vis (MeOH): λ_max_ (log *ε*) = 201 (5.01), 218 (4.92), 262.5 (4.27) nm; NMR data (^1^H NMR: 500 MHz, ^13^C NMR: 125 MHz in DMSO-*d*_6_) see Table 2; HR-(+)ESI-MS: *m*/*z* 704.3806 [M + H]^+^ (calcd. 704.3806 for C_42_H_50_N_5_O_5_^+^), 726.3627 [M + Na]^+^ (calcd. 726.3626 for C_42_H_49_N_5_NaO_5_^+^); *t*_R_ = 14.23 min (LC-ESI-MS). C_42_H_49_N_5_O_5_ (703.41 g/mol).

Cardinalisamide D (**4**): Yellowish-brown amorphous solid; ${\left[\alpha \right]}_{D}^{20}$ –136.0° (*c* 0.1, acetone); UV/Vis (MeOH): *λ*_max_ (log *ε*) = 194 (4.65), 203 (4.97) nm; NMR data (^1^H NMR: 500 MHz, ^13^C NMR: 125 MHz in DMSO-*d*_6_) see Table 3; HR-(+)ESI-MS: *m*/*z* 692.4023 [M + H]^+^ (calcd. 692.4018 for C_38_H_54_N_5_O_7_^+^), 714.3836 [M + Na]^+^ (calcd. 714.3837 for C_38_H_53_N_5_NaO_7_^+^); *t*_R_ = 12.91 min (LC-ESI-MS). C_38_H_53_N_5_O_7_ (691.45 g/mol).

Cardinalisamide B (**5**): Yellowish-brown amorphous solid; ${\left[\alpha \right]}_{D}^{20}$ –93.0° (*c* 0.1, acetone); UV/Vis (MeOH): *λ*_max_ (log *ε*) = 204 (4.83) nm; NMR data (^1^H NMR: 500 MHz, ^13^C NMR: 125 MHz in DMSO-*d*_6_) comparable to those reported in the literature [19,20]; HR-(+)ESI-MS: *m*/*z* 679.3709 [M + H]^+^ (calcd. 679.3701 for C_37_H_51_N_4_O_8_^+^), 701.3521 [M + Na]^+^ (calcd. 701.3521 for C_37_H_50_N_4_NaO_8_^+^); *t*_R_ = 13.58 min (LC-ESI-MS). C_37_H_50_N_4_O_8_ (678.42 g/mol).

Cardinalisamide C (**6**): Yellowish-brown amorphous solid; ${\left[\alpha \right]}_{D}^{20}$ –129.0° (*c* 0.1, acetone); UV/Vis (MeOH): *λ*_max_ (log *ε*) = 201 (4.86), 256.5 (4.13) nm; NMR data (^1^H NMR: 500 MHz, ^13^C NMR: 125 MHz in DMSO-*d*_6_) comparable to those reported in the literature [19,20]; HR-(+)ESI-MS: *m*/*z* 693.3866 [M + H]^+^ (calcd. 693.3867 for C_38_H_53_N_4_O_8_^+^), 715.3676 [M + Na]^+^ (calcd. 715.3677 for C_38_H_52_N_4_NaO_8_^+^); *t*_R_ = 14.68 min (LC-ESI-MS). C_38_H_52_N_4_O_8_ (692.45 g/mol).

### 3.4. Derivatization with Marfey’s Reagent and Elucidation of Amino Acid Configurations

Determination of amino acid stereochemistry of beauveriolide U (**3**) and cardinalisamide D (**4**) was conducted using Marfey’s method, following the experimental procedure outlined by Viehrig et al. [25]. For the hydrolysis, 500 µL of 6 N HCl was added to 0.5 mg of the compound and incubated at 90 °C for 18 h. The resulting hydrolysate was evaporated under vacuum conditions and suspended in 200 µL of Milli-Q water. Subsequently, 20 μL of 1 M NaHCO_3_ and 100 μL of acetone containing 1% derivatization agent Nα-(2,4-dinitro-5-fluorophenyl)-l-alaninamide (FDAA) were added. The mixture was incubated at 40 °C for 40 min and evaporated to dryness under vacuum. Finally, the residual product was diluted in 1 mL MeOH and analyzed using an HPLC system connected to an amaZon speed ESI-MS as described before. The _L_- or _D_- configuration of the amino acids was determined by comparing the observed retention times with those of authentic amino acids subjected to the same derivatization procedure. The retention times (in minutes) of the FDAA-derivatized amino acids were as follows: alanine (_L-_: 5.61, _D-_: 6.40), tryptophan (_L-_: 7.82, _D-_: 8.41; leucine (_L-_: 8.07, _D-_: 9.0), and phenylalanine (_L-_: 8.01, _D-_: 8.81).

### 3.5. Cytotoxicity Assay

In vitro cytotoxic activity of the isolated compounds was tested by applying the MTT (3-(4,5-dimethylthiayol-2-yl)-2,5-diphenyltetrazolium bromide) assay as previously reported [26,27]. The mammalian cell lines used in the tests were sourced from DSMZ and included mouse fibroblasts (L929) and endocervical adenocarcinoma (KB3.1), human lung carcinoma (A549), breast adenocarcinoma (MCF-7), human ovarian cancer (SKOV-3), prostate carcinoma (PC-3), and epidermoid carcinoma cells (A431). Epothilone B was used as the positive control.

### 3.6. Antimicrobial Assay

The antimicrobial activity of the isolated secondary metabolites was determined using our established protocol [26,27], against clinically relevant microorganisms obtained from the German Collection of Microorganisms and Cell Cultures (DSMZ, Braunschweig, Germany). These included *Staphylococcus aureus* (DSM 346), *Bacillus subtilis* (DSM 10), *Acinetobacter baumanii* (DSM 30008), *Escherichia coli* (DSM 1116), *Chromobacterium violaceum* (DSM 30191), *Pseudomonas aeruginosa* (PA14), *Mycolicibacterium smegmatis* (ATCC 700084), *Candida albicans* (DSM 1665), *Mucor hiemalis* (DSM 2656), *Rhodotorula glutinis* (DSM 10134), *Schizosaccharomyces pombe* (DSM 70572) and *Pichia anomala* (DSM 6766). Nystatin was used as an antifungal positive control whereas oxytetracycline, ciprofloxacin, gentamicin, and kanamycin were used as positive controls against Gram-positive and Gram-negative bacteria.

### 3.7. Nematicidal Activity

The nematicidal activity assay was conducted following a previously described procedure with slight modifications [28,29]. *C. elegans* was cultured on nematode growth medium (NGM) containing 3 g NaCl, 20 g agar, 2.5 g peptone, 1 mL 1 M CaCl_2_, 25 mL of 1 M (pH 6.0) KPO_4_, 1 mL 1 M MgSO_4_, and 1 mL cholesterol (5 mg/mL in ethanol) per liter of medium. Plates were coated with *Escherichia coli* strain OP50, which served as the food source for nematodes. Synchronization of the nematode population was performed using a high egg density plate (~120 h). The plate was rinsed three times with 4 mL of 0.9% NaCl solution and then transferred to a 15 mL falcon tube. The nematode suspension was centrifuged at 1000 rpm for 3 min, and the supernatant was discarded. This procedure was repeated until a clear nematode suspension was obtained. After the last washing step, 2 mL of the suspension was mixed with 5 mL of bleaching solution (1 mL sodium hypochlorite solution, 0.5 mL 5 M NaOH, and 3.5 mL Milli-Q water). The solution was gently shaken for approximately 5 min to break down the nematode tissue, and monitored under the microscope. The reaction was stopped by adding 7 mL 0.9% NaCl solution when traces of adults were still visible. Subsequently, the suspension was centrifuged at 2500 rpm for 2 min and washed 3 times as previously described. After the final washing step, the supernatant was removed, and the volume was adjusted to 7 mL with 0.9% NaCl solution. The nematode egg suspension was incubated at 23 °C on a rotary shaker at 80 rpm for 18 h. The hatched nematodes were transferred to a fresh NGM plate supplemented with *E.coli* OP50. After 50–70 h, the J4 and adult *C. elegans* were washed from the plate as mentioned above. The nematode concentration was determined and diluted to approximately 1000 nematodes/mL.

Pure compounds were tested at concentrations of 10, 50, and 100 μg/mL in 48-well microtiter plates. The compounds, dissolved in MeOH, were added to the well plate and subsequently dried under nitrogen. After complete evaporation of the solvent, 300 μL of the nematode suspension (1000 nematodes/mL) was added to the compounds. Each treatment was replicated three times. MeOH was used as the negative control while 1 μg/mL Ivermectin served as the positive control. Nematodes were monitored 15 min after inoculation, and the plates were incubated at 24 °C and 150 rpm for 18 h. After incubation, both alive and dead nematodes were counted in three replicates under a stereomicroscope, and the mortality rate was calculated. Erect and non-moving nematodes were considered dead. A compound was deemed active if it resulted in mortality rates of at least 50% at a concentration of 100 μg/mL (lethal dose, 50%). The observed percentage of dead nematodes was corrected by considering the natural mortality observed in the negative control, using the Schneider-Orelli formula [30].

## 4. Conclusions

In this study, we explored, both chemically and biologically, the total mycelial extract derived from a solid-state rice culture of the entomopathogenic fungus *B. roseostromatus* BCC56290. Chemically, six secondary metabolites were successfully distinguished including four unprecedentedly reported natural products, namely one bioxanthracene ES-242-9 (**1**), three cyclodepsipeptides (**2**–**4**) together with two known congeners, cardinalisamides B (**5**) and C (**6**). Among the different bioassays conducted, compounds **5** and **6** revealed significant cytotoxic activity against the tested cell lines with minor or no toxicity against the normal cells that might have a positive impact on their specificity toward cancerous cells. In the antimicrobial assay, none of the isolated compounds revealed significant activity against any tested bacterial or fungal pathogens. In nematicidal activity against *C. elegans*, both ES-242-9 (**1**) and **6** revealed comparable mortality rates, however, revealing neither cytotoxic nor antimicrobial activity by **1** supports its potential as a candidate for further development of a biocontrol agent.

## Figures and Tables

**Table 1:** ^1^H and ^13^C NMR data of ES-242-9 (**1**).

Pos.	δ_C_, *^a^*^,*c*^ Type	δ_H_ *^b^* Multi (*J* [Hz])	Pos.	δ_C_, *^a^*^,*c*^ Type	δ_H_ *^b^* Multi (*J* [Hz])
1	64.3, CH_2_	*α* 4.66 d (15.4) *β* 5.00 d (15.4)	1′	64.1, CH_2_	*α* 4.59 d (15.2) *β* 4.82 d (15.2)
3	69.9, CH	3.64 m	3′	74.9, CH	3.31 overlapped
4	34.2, CH_2_	*α* 1.96 dd (16.7, 10.9) *β* 2.20 dd (16.7, 3.0)	4′	69.6, CH	3.99 t (8.4)
4a	134.1, C		4a′	139.2, C	
5	98.3, CH	6.04 d (2.2)	5′	110.2, C	
6	156.7, C		6′	152.0, C	
7	96.3, CH	6.56 d (2.2)	7′	97.3, CH	6.73 s
8	157.3, C		8′	156.2, C	
8a	108.8, C		8a′	108.1, C	
9	148.1, C		9′	148.3, C	
9a	114.3, C		9a′	113.8, C	
10	122.0, C		10′	111.9, CH	6.57 d (1.2)
10a	134.9, C		10a′	135.0, C	
11	21.6, CH_3_	1.06 d (6.2)	11′	18.6, CH_3_	1.18 d (6.0)
6-OCH_3_	54.9, CH_3_	3.46 s	4′-OH	-	5.19 d (8.0)
8-OCH_3_	56.5, CH_3_	4.04 s	6′-OH	-	9.20 br s
9-OH	-	9.49 s	8′-OCH_3_	56.1, CH_3_	4.05 s
			9′-OH	-	9.46 s

Measured in DMSO-*d*_6_ *^a^* at 125 MHz/ *^b^* at 500 MHz. *^c^* Assignment confirmed by HMBC and HSQC spectra.

**Table 2:** ^1^H and ^13^C NMR data of beauveriolides T (**2**) and U (**3**).

Pos.	2		3	
	δ_C_, *^a^*^,*c*^ Type	δ_H_ *^b^* Multi (*J* [Hz])	δ_C_, *^a^*^,*c*^ Type	δ_H_ *^b^* Multi (*J* [Hz])
HMDA				
1	170.3, CO		170.3, CO	
2	35.8, CH_2_	*α* 2.34 dd (13.8, 9.2) *β* 2.46 dd (13.8, 4.2)	35.6, CH_2_	*α* 2.37 dd (14.5, 8.3) *β* 2.41 dd (14.5, 4.7)
3	76.1, CH	4.83 ddd (9.7, 5.8, 4.2)	76.1, CH	4.85 td (7.4, 4.9)
4	35.2, CH	1.91 ddd (9.8, 5.9, 2.4)	34.9, CH	2.01 m
5	31.0, CH_2_	*α* 0.96 m; *β* 1.34 m	31.1, CH_2_	*α* 0.95 m; *β* 1.36 m
6	26.5, CH_2_	*α* 1.12 overlapped *β* 1.28 overlapped	26.4, CH_2_	*α* 1.12 overlapped *β* 1.29 overlapped
7	31.3, CH_2_	1.23 m	31.3, CH_2_	1.23 m
8	29.0, CH_2_	1.23 m	29.0, CH_2_	1.23 m
9	22.1, CH_2_	1.25 m	22.2, CH_2_	1.25 m
10	14.0, CH_3_	0.85 t (6.9)	14.0, CH_3_	0.84 t (6.8)
11	15.3, CH_3_	0.75 d (6.9)	15.4, CH_3_	0.75 d (6.9)
Trp/Trp^1^				
1′	171.4, CO		170.2, CO	
2′	55.9, CH	4.21 q (7.6)	56.5, CH	4.18 overlapped
3′	25.6, CH_2_	*α* 3.02 dd (14.5, 7.9) *β* 3.12 dd (14.5, 7.6)	25.6, CH_2_	*α* 3.07 dd (15.3, 7.1) *β* 3.11 overlapped
4′	109.8, C		110.0, C	
4a′	127.0, C		127.1, C	
5′	118.0, CH	7.51 d (8.0)	118.2, CH	7.48 dd (8.0)
6′	118.1, CH	6.97 ddd (8.0, 6.9, 1.0)	118.4, CH	6.98 overlapped
7′	120.8, CH	7.06 ddd (8.0, 6.9, 1.2)	121.0, CH	7.07 m
8′	111.2, CH	7.32 d (8.0)	111.5, CH	7.35 d (8.1)
8a′	136.0, C		136.1, C	
9′-NH	-	10.86 d (2.4)	-	10.83 d (2.5)
10′	123.3, CH	7.11 d (2.4)	123.6, CH	7.10 d (2.5)
NH	-	8.51 d (7.4)	-	8.43 d (7.7)
Ala/Trp^2^				
1″	170.7, CO		171.9, CO	
2″	48.6, CH	3.82 p (6.9)	54.2, CH	4.15 overlapped
3″	15.4, CH_3_	1.11 d (6.9)	25.0, CH_2_	*α* 2.94 dd (14.8, 7.1) *β* 3.25 dd (14.7, 7.1)
4″			111.0, C	
4a″			127.4, C	
5″			118.3, CH	7.47 d (7.9)
6″			118.2, CH	6.95 overlapped
7″			120.8, CH	7.04 m
8″			111.3, CH	7.32 d (8.2)
8a″			136.0, C	
9″-NH			-	10.69 d (2.4)
10″			123.0, CH	6.81 d (2.4)
NH	-	8.47 d (7.3)	-	8.30 d (7.5)
Phe				
1‴	168.7, CO		169.1, CO	
2‴	54.8, CH	4.62 dd (9.1, 7.8)	55.0, CH	4.60 q (8.0)
3‴	37.6, CH_2_	*α* 2.86 dd (13.9, 7.5) *β* 2.92 dd (13.9, 8.0)	37.3, CH_2_	2.90 q (6.5)
4‴	136.7, C		136.8, C	
5‴, 9‴	128.6, CH	7.19 overlapped	128.8, CH	7.18 overlapped
6‴, 8‴	126.5, CH	7.21 overlapped	126.7, CH	7.21 overlapped
7‴	128.1, CH	7.26 t (7.1)	128.3, CH	7.24 t (7.1)
NH	-	7.31 d (8.8)	-	7.77 d (8.7)

Measured in DMSO-*d*_6_ *^a^* at 125 MHz/ *^b^* at 500 MHz. *^c^* Assignment confirmed by HMBC and HSQC spectra.

**Table 3:** ^1^H and ^13^C NMR data of cardinalisamide D (**4**).

Pos.	δ_C_, *^a^*^,*c*^ Type	δ_H_ *^b^* Multi (*J* [Hz])	Pos.	δ_C_, *^a^*^,*c*^ Type	δ_H_ *^b^* Multi (*J* [Hz])
L-Pla			L-Phe		
1	168.0, CO		20	169.8, CO	
2	74.0, CH	5.05 dd (10.5, 3.9)	21	55.8, CH	4.03 ddd (11.7, 8.0, 3.4)
3	37.4, CH_2_	*α* 2.90 dd (14.1, 10.5) *β* 3.15 overlapped	22	34.2, CH_2_	*α* 3.19 overlapped *β* 3.34 overlapped
4	137.2, C		23	139.5, C	
5, 9	129.1, CH	7.15 m (2H)	24, 28	129.2, CH	7.28 m (2H)
6, 8	128.0, CH	7.27 m (2H)	25, 27	126.3, CH	7.20 m (2H)
7	125.9, CH	7.18 m (1H)	26	128.8, CH	7.21 m (1H)
L-NMe-Ala^1^			21-NH	-	7.92 d (7.9)
10	170.0, CO		L-NMe-Ala^2^		
11	58.0, CH	3.48 q (7.0)	29	170.2, CO	
12	13.2, CH_3_	1.19 d (7.0)	30	60.8, CH	3.48 q (7.0)
13	36.5, CH_3_	3.16 s	31	12.8, CH_3_	1.32 d (7.0)
L-Leu^1^			32	37.6, CH_3_	3.12 s
14	171.2, CO		L-Leu^2^		
15	45.8, CH	4.93 dt (9.5, 7.0)	33	171.8, CO	
16	40.4, CH_2_	*α* 1.57 overlapped *β* 1.64 overlapped	34	46.1, CH	4.81 td (8.2, 6.3)
17	23.5, CH	1.58 overlapped	35	39.6, CH_2_	*α* 1.47 overlapped *β* 1.53 overlapped
18	21.5–23.0, CH_3_	0.84–0.89 overlapped	36	24.0, CH	1.45 overlapped
19	21.5–23.0, CH_3_	0.84–0.89 overlapped	37	21.5–23.0, CH_3_	0.84–0.89 overlapped
15-NH	-	7.85 d (9.6)	38	21.5–23.0, CH_3_	0.84–0.89 overlapped
			34-NH	-	7.42 d (8.9)

Measured in DMSO-*d*_6_ *^a^* at 125 MHz/ *^b^* at 500 MHz. *^c^* Assignment confirmed by HMBC and HSQC spectra.

**Table 4:** Cytotoxic (IC_50_ in µM) activity results of **1**–**6** against mammalian cells.

	IC_50_ (µM)						Positive Control
Test Cell Line	1	2	3	4	5	6	Epothilone B (nM)
L929 (murine)	**	*	*	**	**	26.0	0.65
KB3.1 (cervix)	29.2	*	*	36.2	2.2	8.4	0.17
PC-3 (prostate)	n.t.	n.t.	n.t.	n.t.	2.5	7.5	0.09
MCF-7 (breast)	n.t.	n.t.	n.t.	n.t.	6.9	13.9	0.07
SKOV-3 (ovary)	n.t.	n.t.	n.t.	n.t.	25.0	12.3	0.09
A431 (skin)	n.t.	n.t.	n.t.	n.t.	4.3	8.5	0.06
A549 (lung)	n.t.	n.t.	n.t.	n.t.	11.5	11.1	0.05

(*): Slight inhibition of cell proliferation, (**): no cytotoxic activity observed, n.t.: not tested.

## Data Availability

Data are contained within the article and Supplementary Materials. The authors provide the raw NMR files upon request.

## Supplementary Materials

The following supporting information can be downloaded at: https://www.mdpi.com/article/10.3390/antibiotics13070585/s1, Figures S1–S8. HPLC, LR-/HR-ESI-MS, 1D/2D NMR spectra of **1**; Figures S9–S16. HPLC, LR-/HR-ESI-MS, 1D/2D NMR spectra of **2**; Figures S17–S24. HPLC, LR-/HR-ESI-MS, 1D/2D NMR spectra of **3**; Figures S25–S32. HPLC, LR-/HR-ESI-MS, 1D/2D NMR spectra of **4**; Figures S33–S38. HPLC, LR-/HR-ESI-MS, 1D/2D NMR spectra of **5**; Figures S39–S44. HPLC, LR-/HR-ESI-MS, 1D/2D NMR spectra of **6**; Figures S45–S48. HPLC, LR-ESI-MS of advanced Marefy’s method for compounds **3** and **4**. Figure S49. Maximum likelihood phylogenetic tree inferred from 117 taxa of Cordycipitaceae based on combined ITS, LSU, EF1, and RPB1 sequence data. Tables S1 and S2: Antimicrobial and nematicidal activity assays of **1**–**6**.
