# Supplementary material for: Bioactive Bioxanthracene and Cyclodepsipeptides from the Entomopathogenic Fungus Blackwellomyces roseostromatus BCC56290
Source: Antibiotics (Basel). 2024 Jun 24;13(7):585. doi: 10.3390/antibiotics13070585 (PMC11273930; doi:10.3390/antibiotics13070585)
Supplement: Supplementary file 1 [file antibiotics-13-00585-s001.zip › antibiotics-3051533-supplementary.pdf]

Supporting Information for:

Bioactive Bioxanthracene and Cyclodepsipeptides from the Entomopathogenic Fungus *Blackwellomyces roseostromatus* BCC56290

Kunthida Phutthacharoen,[1,2,a] Natalia A. Llanos-López,[1,3,a] Rita Toshe,[1,3] Wasana Noisripoom,[4] Artit Khonsanit,[4] J. Jennifer Luangsa-ard,[4] Kevin D. Hyde,[2,5] Sherif S. Ebada,*[1,6] and Marc Stadler*[1,3]

[1] Department of Microbial Drugs, Helmholtz Centre for Infection Research GmbH (HZI), Inhoffenstraße 7, 38124 Braunschweig, Germany

[2] Center of Excellence in Fungal Research, Mae Fah Luang University, Chiang Rai 57100, Thailand

[3] Institute of Microbiology, Technische Universität Braunschweig, Spielmannstraße 7, 38106 Braunschweig, Germany

[4] National Center for Genetic Engineering and Biotechnology (BIOTEC), National Science and Technology Development Agency (NSTDA), 113 Thailand Science Park, Phahonyothin Rd., Khlong Nueng, Khlong Luang, Pathum Thani 12120, Thailand

[5] School of Science, Mae Fah Luang University, Chiang Rai 57100, Thailand

[6] Department of Pharmacognosy, Faculty of Pharmacy, Ain Shams University, 11566 Cairo, Egypt

[a] The authors equally contributed to this work.

* Correspondence: sherif.elsayed@helmholtz-hzi.de; sherif_elsayed@pharma.asu.edu.eg (S.S.E.); Marc.Stadler@helmholtz-hzi.de (M.S.); Tel.: +49-531-6181-4240; Fax +49-531-6181-9499

Contents of Supporting Information

#	Contents	Page
**1**	Figure S1. LR-ESI-MS of **1**.	**S4**
**2**	Figure S2. HR-ESI-MS of **1**.	**S5**
**3**	Figure S3. ^{1}H NMR spectrum of **1** in DMSO-d_6 at 500 MHz.	**S6**
**4**	Figure S4. DEPTQ spectrum of **1** in DMSO-d_6 at 125 MHz.	**S7**
**5**	Figure S5. ^{1}H–^{1}H COSY spectrum of **1** in DMSO-d_6 at 500 MHz.	**S8**
**6**	Figure S6. HMBC spectrum of **1** in DMSO-d_6 at 500 MHz.	**S9**
**7**	Figure S7. HSQC spectrum of **1** in DMSO-d_6 at 500 MHz.	**S10**
**8**	Figure S8. ROESY spectrum of **1** in DMSO-d_6 at 500 MHz.	**S11**
**9**	Figure S9. LR-ESI-MS of **2**.	**S12**
**10**	Figure S10. HR-ESI-MS of **2**.	**S13**
**11**	Figure S11. ^{1}H NMR spectrum of **2** in DMSO-d_6 at 500 MHz.	**S14**
**12**	Figure S12. DEPTQ spectrum of **2** in DMSO-d_6 at 125 MHz.	**S15**
**13**	Figure S13. ^{1}H-^{1}H COSY spectrum of **2** in DMSO-d_6 at 500 MHz.	**S16**
**14**	Figure S14. HMBC spectrum of **2** in DMSO-d_6 at 500 MHz.	**S17**
**15**	Figure S15. HSQC spectrum of **2** in DMSO-d_6 at 500 MHz.	**S18**
**16**	Figure S16. ROESY spectrum of **2** in DMSO-d_6 at 500 MHz.	**S19**
**17**	Figure S17. LR-ESI-MS of **3**.	**S20**
**18**	Figure S18. HR-ESI-MS of **3**.	**S21**
**19**	Figure S19. ^{1}H NMR spectrum of **3** in DMSO-d_6 at 500 MHz.	**S22**
**20**	Figure S20. DEPTQ spectrum of **3** in DMSO-d_6 at 125 MHz.	**S23**
**21**	Figure S21. ^{1}H-^{1}H COSY spectrum of **3** in DMSO-d_6 at 500 MHz.	**S24**
**22**	Figure S22. HMBC spectrum of **3** in DMSO-d_6 at 500 MHz.	**S25**
**23**	Figure S23. HSQC spectrum of **3** in DMSO-d_6 at 500 MHz.	**S26**
**24**	Figure S24. ROESY spectrum of **3** in DMSO-d_6 at 700 MHz.	**S27**
**25**	Figure S25. LR-ESI-MS of **4**.	**S28**
**26**	Figure S26. HR-ESI-MS of **4**.	**S29**
**27**	Figure S27. ^{1}H NMR spectrum of **4** in DMSO-d_6 at 500 MHz.	**S30**
**28**	Figure S28. DEPTQ spectrum of **4** in DMSO-d_6 at 125 MHz.	**S31**
**29**	Figure S29. ^{1}H-^{1}H COSY spectrum of **4** in DMSO-d_6 at 500 MHz.	**S32**
**30**	Figure S30. HMBC spectrum of **4** in DMSO-d_6 at 500 MHz.	**S33**
**31**	Figure S31. HSQC spectrum of **4** in DMSO-d_6 at 500 MHz.	**S34**
**32**	Figure S32. ROESY spectrum of **4** in DMSO-d_6 at 700 MHz.	**S35**
**33**	Figure S33. LR-ESI-MS of **5**.	**S36**
**34**	Figure S34. HR-ESI-MS of **5**.	**S37**
**35**	Figure S35. ^{1}H NMR spectrum of **5** in DMSO-d_6 at 500 MHz.	**S38**
**36**	Figure S36. ^{1}H-^{1}H COSY spectrum of **5** in DMSO-d_6 at 500 MHz.	**S39**
**37**	Figure S37. HMBC spectrum of **5** in DMSO-d_6 at 500 MHz.	**S40**
**38**	Figure S38. HSQC spectrum of **5** in DMSO-d_6 at 500 MHz.	**S41**
**39**	Figure S39. LR-ESI-MS of **6**.	**S42**
**40**	Figure S40. HR-ESI-MS of **6**.	**S43**
**41**	Figure S41. ^{1}H NMR spectrum of **6** in DMSO-d_6 at 500 MHz.	**S44**
**42**	Figure S42. ^{1}H-^{1}H COSY spectrum of **6** in DMSO-d_6 at 500 MHz.	**S45**
**43**	Figure S43. HMBC spectrum of **6** in DMSO-d_6 at 500 MHz.	**S46**
**44**	Figure S44. HSQC spectrum of **6** in DMSO-d_6 at 500 MHz.	**S47**
**45**	Figure S45. LC-ESI-MS spectra of beauverolide U (**3**), D-tryptophan and L-tryptophan with FDAA. Top-bottom: beauverolide U (**3**) + FDAA, D-	**S48**

	tryptophan + FDAA, L-tryptophan + FDAA. $[M+H]^+$ of the adduct is displayed in blue. Extracted ion chromatogram (*m/z* 457) and UV-chromatogram at 190-600 nm are shown. Bar indicates identical MS-Peaks (pink L-tryptophan).	
**46**	Figure S46. LC-ESI-MS spectra of cardinalisamide D (**4**), beauverolide U (**3**), DL-phenylalanine and L-phenylalanine with FDAA. Top-bottom: cardinalisamide D (**4**) + FDAA, beauverolide U (**3**) + FDAA, DL-phenylalanine + FDAA, L-phenylalanine + FDAA. $[M+H]^+$ of the adducts are displayed in blue. Extracted ion chromatogram (*m/z* 418) and UV-chromatogram at 190-600 nm are shown. Bars indicate identical MS-Peaks (pink L-phenylalanine and blue D-phenylalanine).	**S48**
**47**	Figure S47. LC-ESI-MS spectra of cardinalisamide D (**4**), DL-alanine and L-alanine with FDAA. Top-bottom: cardinalisamide D (**4**) + FDAA, DL-alanine + FDAA, L-alanine + FDAA. $[M+H]^+$ of the adducts is displayed in blue. Extracted ion chromatogram (*m/z* 342) and UV-chromatogram at 190-600 nm are shown. Bar indicates identical MS-Peaks (pink L-alanine).	**S49**
**48**	Figure S48. LC-ESI-MS spectra of cardinalisamide D (**4**), DL-leucine and L-leucine with FDAA. Top-bottom: cardinalisamide D (**4**) + FDAA, DL-leucine + FDAA, L-leucine + FDAA. $[M+H]^+$ of the adducts is displayed in blue. UV-chromatogram at 190-600 nm is shown. Bar indicates identical MS-Peaks (pink L-leucine).	**S49**
**49**	Figure S49. Maximum likelihood phylogenetic tree inferred from 117 taxa of Cordycipitaceae based on combined ITS, LSU, *EF1* and *RPB1* sequence data. MLBP values ≥70% are given above the nodes. Strain/culture numbers are given after the taxon names. The tree is rooted with *Purpureocillium lilacinum* CBS431.87 and CBS284.36. Newly generated sequence is in **blue**.	**S50**
**50**	Table S1. Minimum inhibitory concentration (MIC) of compounds **1–6**.	**S51**
**51**	Table S2. Nematicidal activity of compounds **1–6**.	**S51**

Generic Display Report

Analysis Info

		Acquisition Date	07.07.2023 04:32:07
Analysis Name	S:\DATA\AmaZon\gph22_Kunthide-Gift Phutthacharoen\07-23\MY 07796-F3 R1-F4_RC7_01_14786.d		
Method	14786.m	Operator	Lab
Sample Name	MY 07796-F3 R1-F4	Instrument	amaZon speed
Comment			

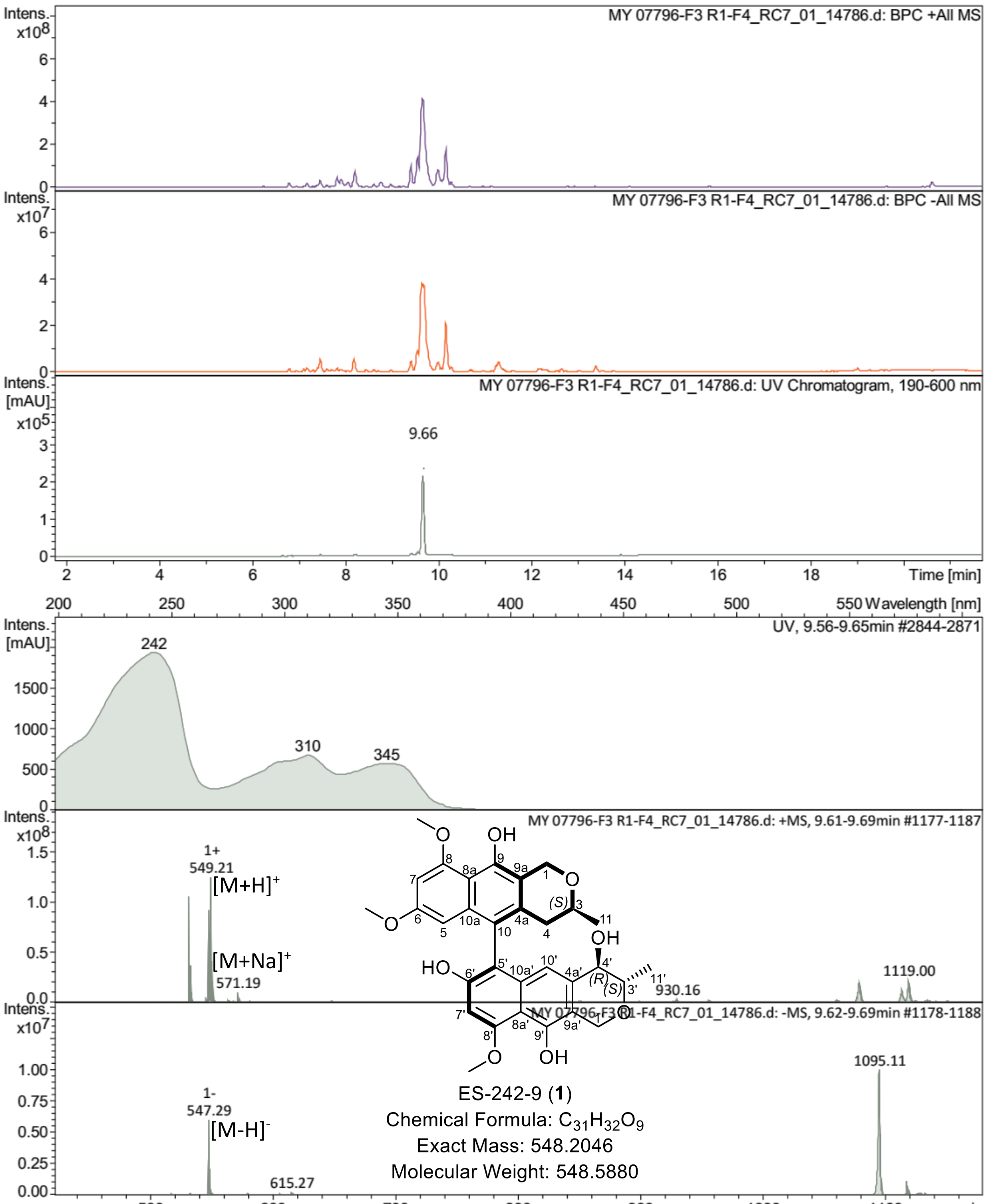

Bruker Compass DataAnalysis 4.4 printed: 19.07.2023 13:08:05 by: sel22 Page 1 of 1

Figure S1. LR-ESI-MS of **1**.

Display Report

Analysis Info			
		Acquisition Date	10.07.2023 17:50:38
Analysis Name	S:\DATA\MaXis\GPH22_Gift_Kunthide_Phutthacharoen\23_07\MY07996-F3R1-F4_27_01_12254.d		
Method	pos_säure_10000_screening_ms_100_2500_line.m	Operator	ate06
Sample Name	MY07996-F3 R1-F4	Instrument	maXis 255552.00037
Comment	Screening01<br>Waters Acquity UPLC BEH C_18 1,7um 2.1x50mm		

Acquisition Parameter

Source Type	ESI	Ion Polarity	Positive	Set Nebulizer	4.0 Bar
Focus	Not active	Set Capillary	4500 V	Set Dry Heater	200 °C
Scan Begin	50 m/z	Set End Plate Offset	-500 V	Set Dry Gas	10.0 l/min
Scan End	2500 m/z	Set Charging Voltage	0 V	Set Divert Valve	Waste
		Set Corona	0 nA	Set APCI Heater	0 °C

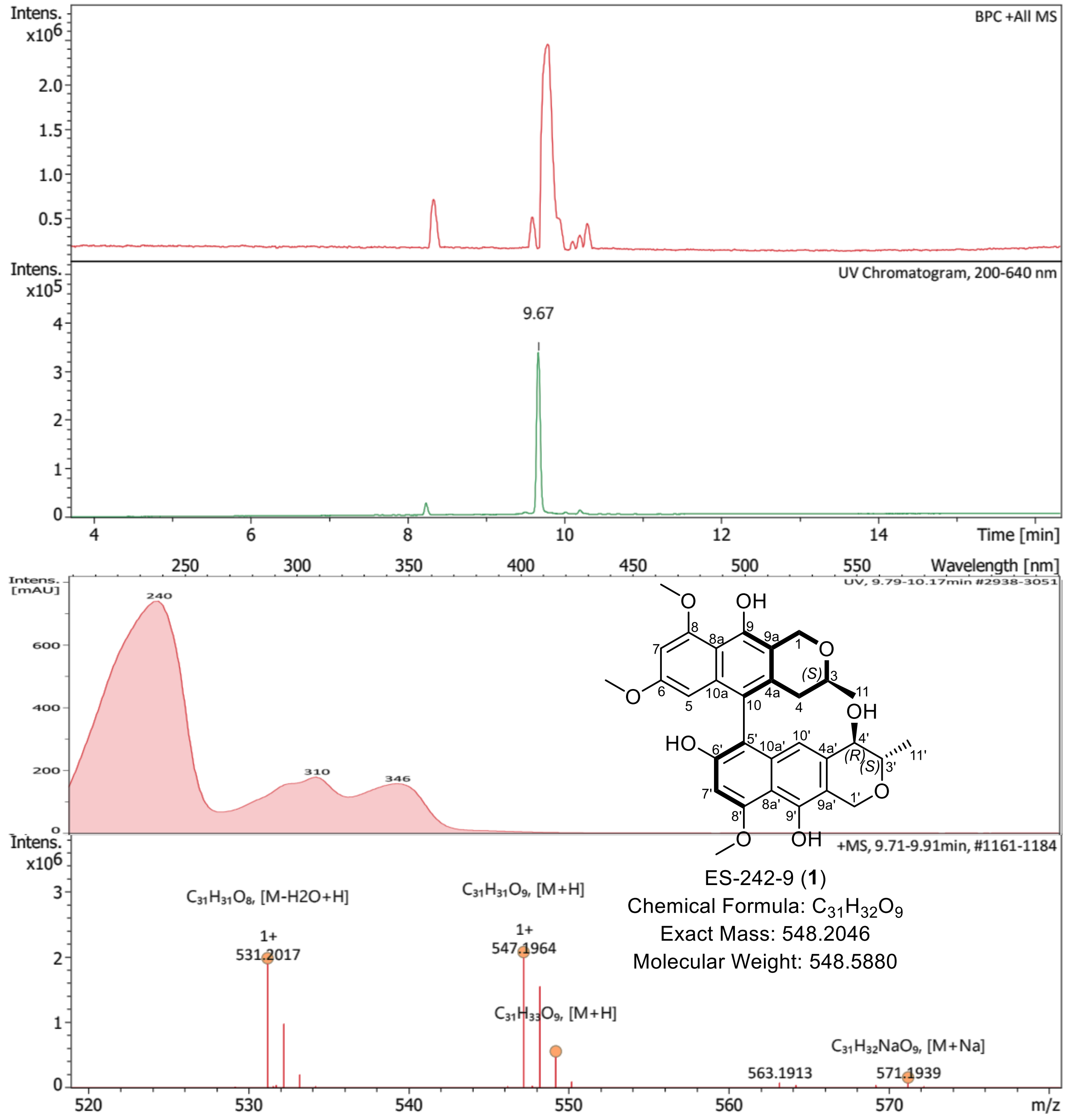

MY07996-F3 R1-F4_27_01_12254.d

Bruker Compass DataAnalysis 6.1 printed: 15.03.2024 09:22:06 by: sel22 Page 1 of 1

Figure S2. HR-ESI-MS of **1**.

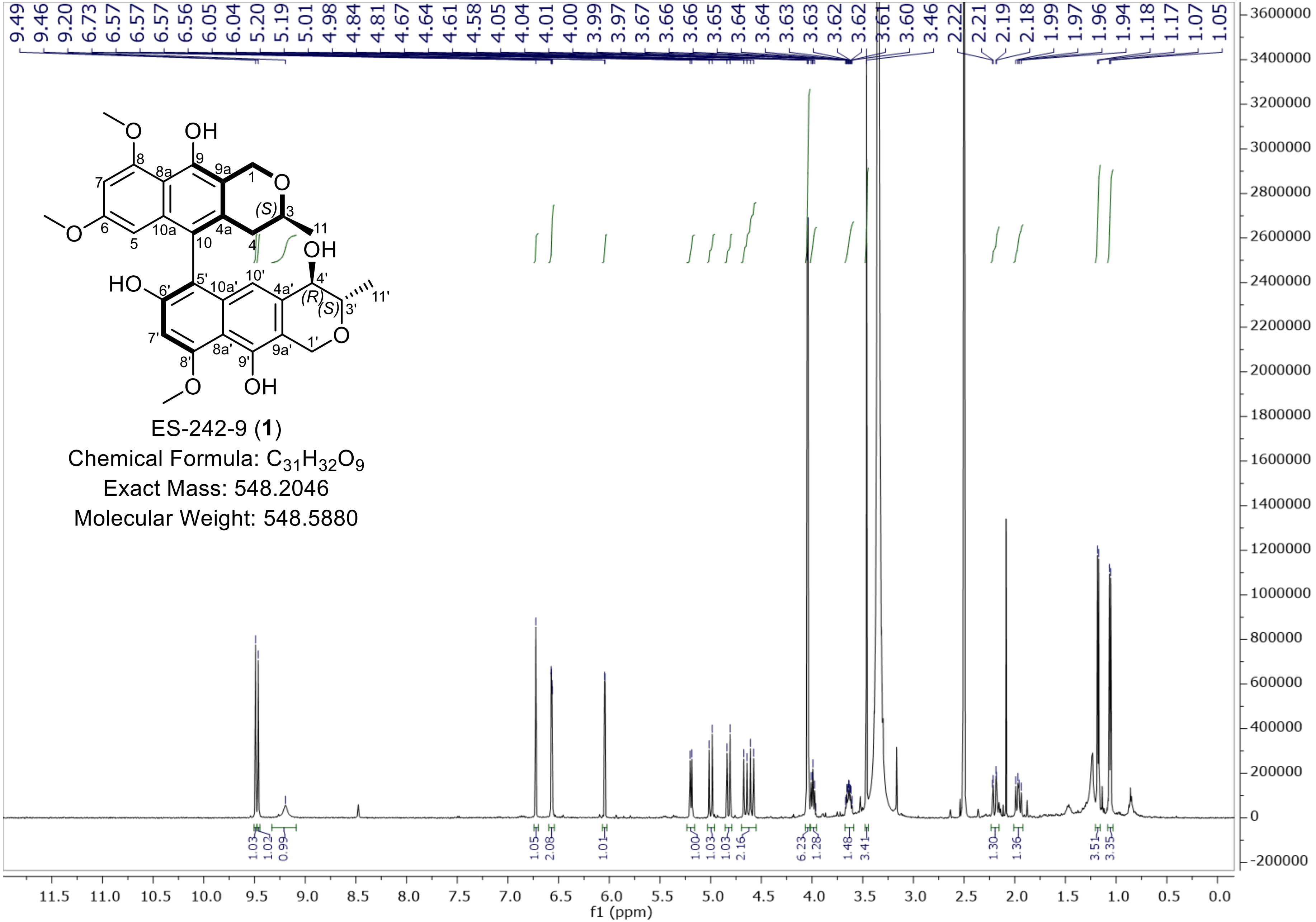

Figure S3. ^{1}H NMR spectrum of **1** in DMSO-d_6 at 500 MHz.

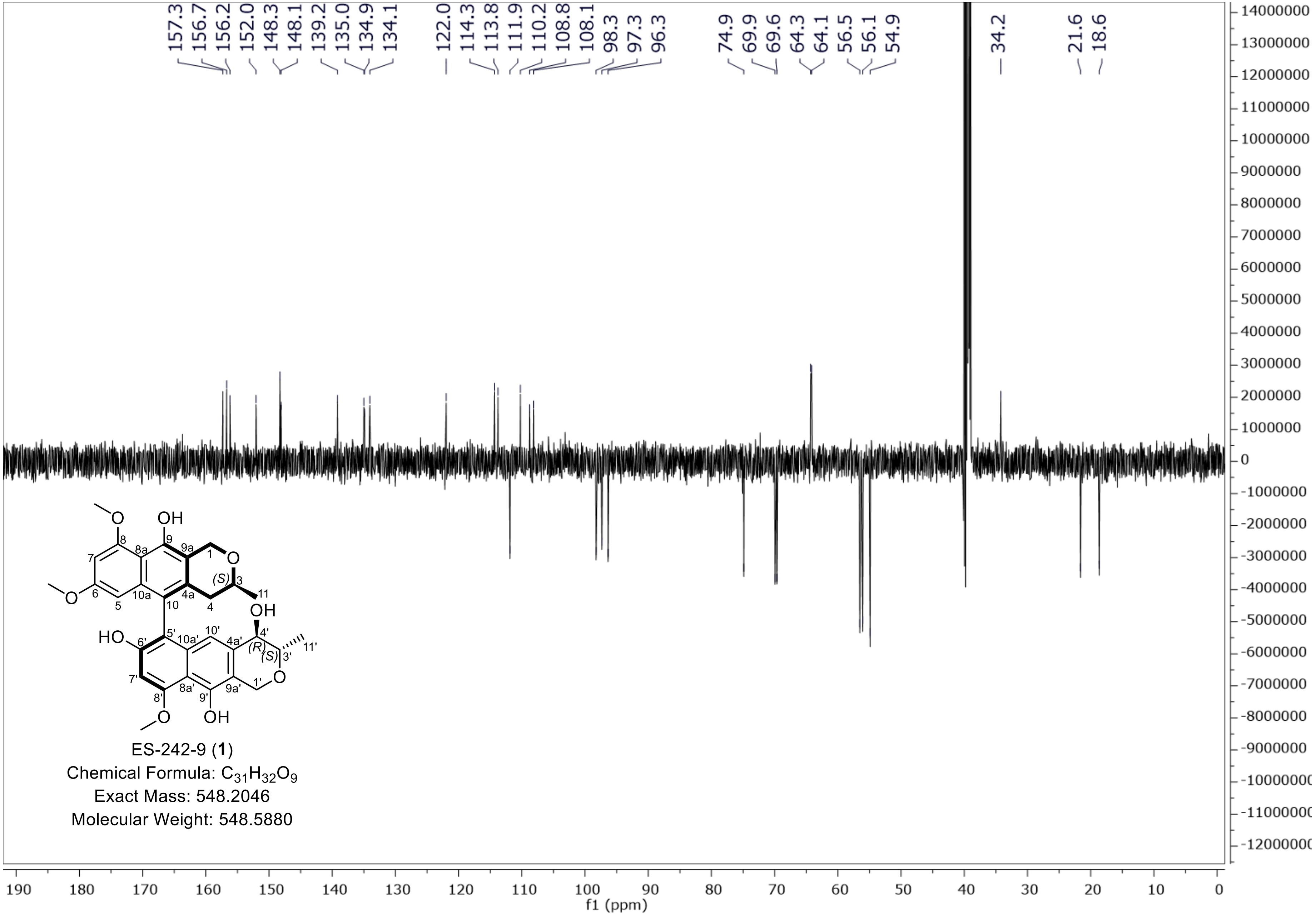

ES-242-9 (**1**)
Chemical Formula: $C_{31}H_{32}O_9$
Exact Mass: 548.2046
Molecular Weight: 548.5880

Figure S4. DEPTQ spectrum of **1** in DMSO-d_6 at 125 MHz.

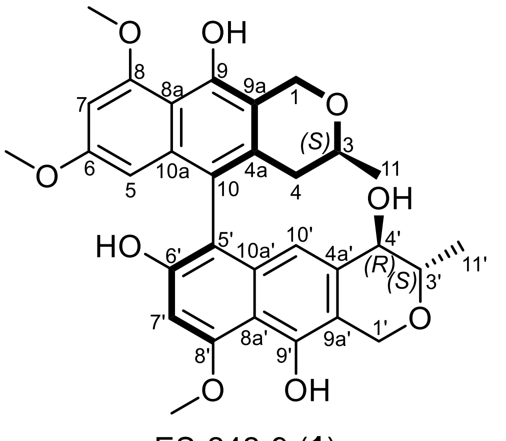

ES-242-9 (**1**)
Chemical Formula: $C_{31}H_{32}O_9$
Exact Mass: 548.2046
Molecular Weight: 548.5880

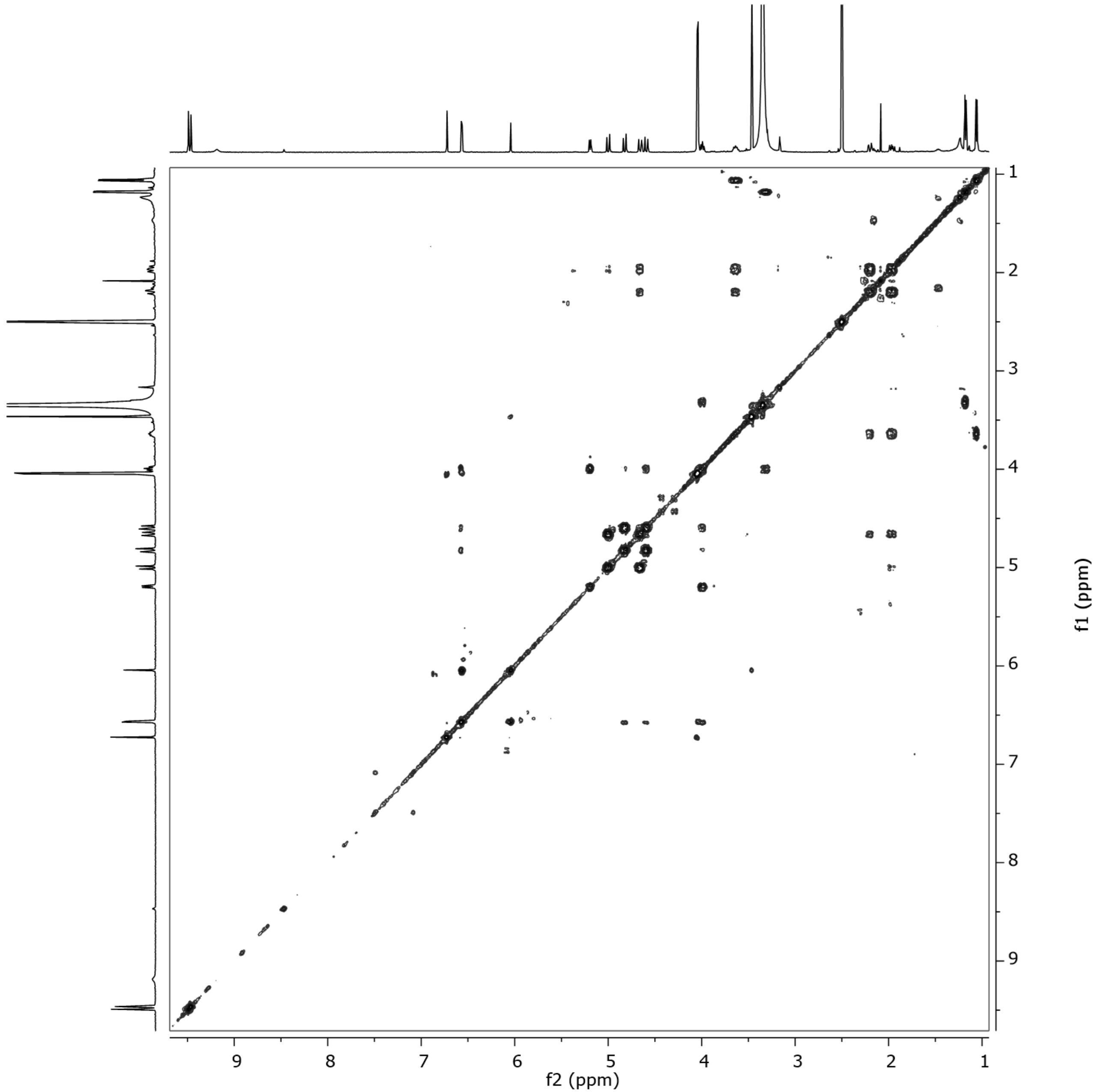

Figure S5. ^1H–^1H COSY spectrum of **1** in DMSO-d_6 at 500 MHz.

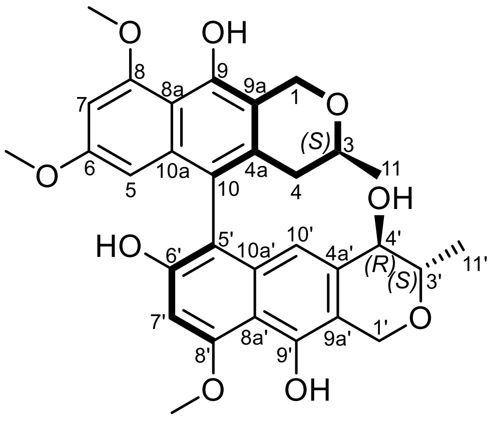

ES-242-9 (**1**)
Chemical Formula: $C_{31}H_{32}O_9$
Exact Mass: 548.2046
Molecular Weight: 548.5880

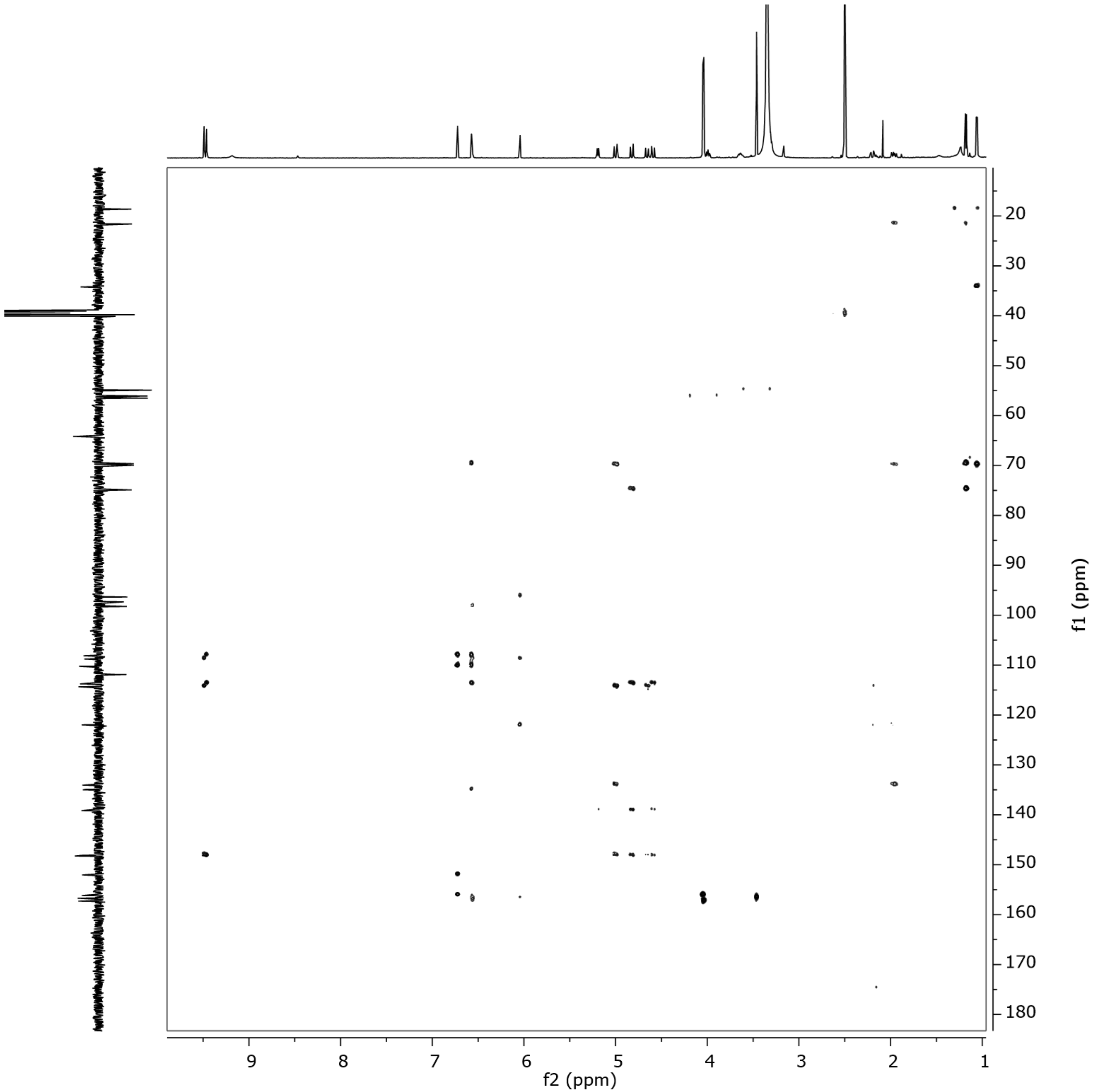

Figure S6. HMBC spectrum of **1** in DMSO-d_6 at 500 MHz.

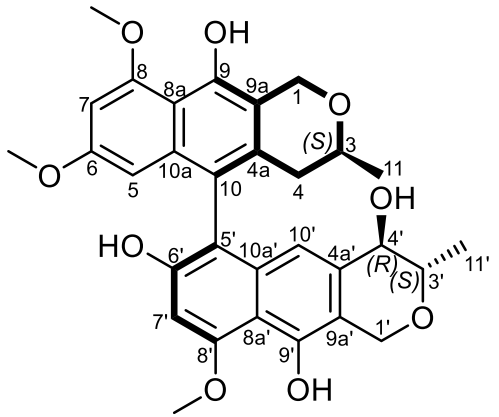

ES-242-9 (**1**)
Chemical Formula: $C_{31}H_{32}O_9$
Exact Mass: 548.2046
Molecular Weight: 548.5880

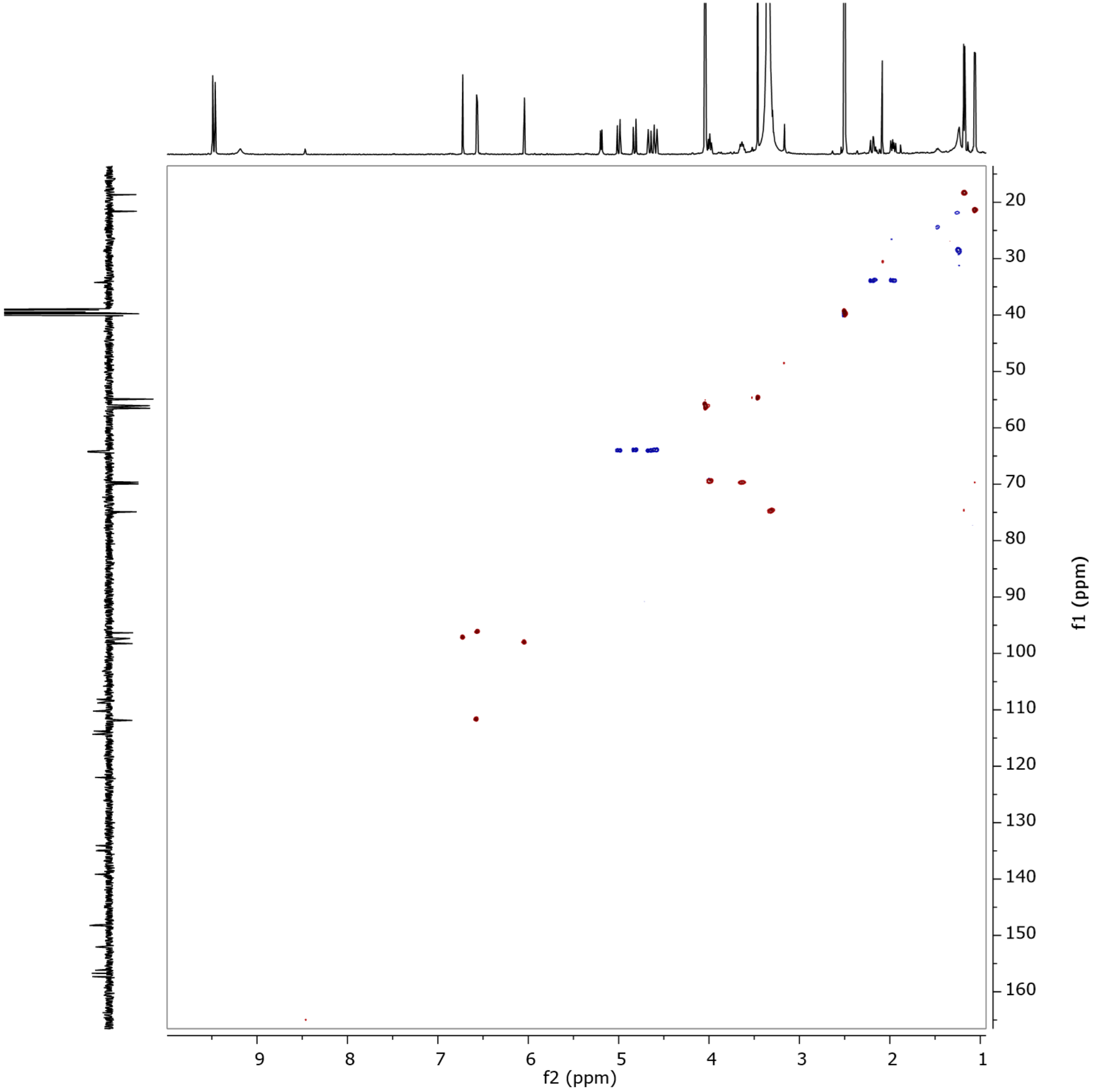

Figure S7. HSQC spectrum of **1** in DMSO-d_6 at 500 MHz.

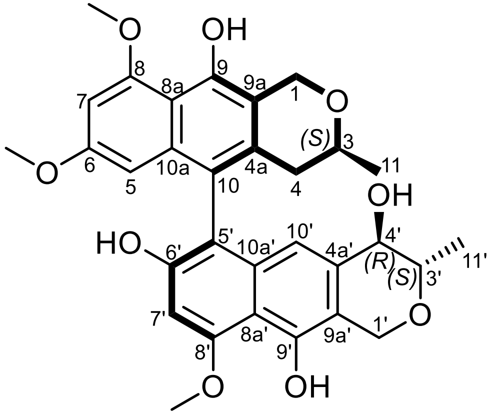

ES-242-9 (**1**)
Chemical Formula: $C_{31}H_{32}O_9$
Exact Mass: 548.2046
Molecular Weight: 548.5880

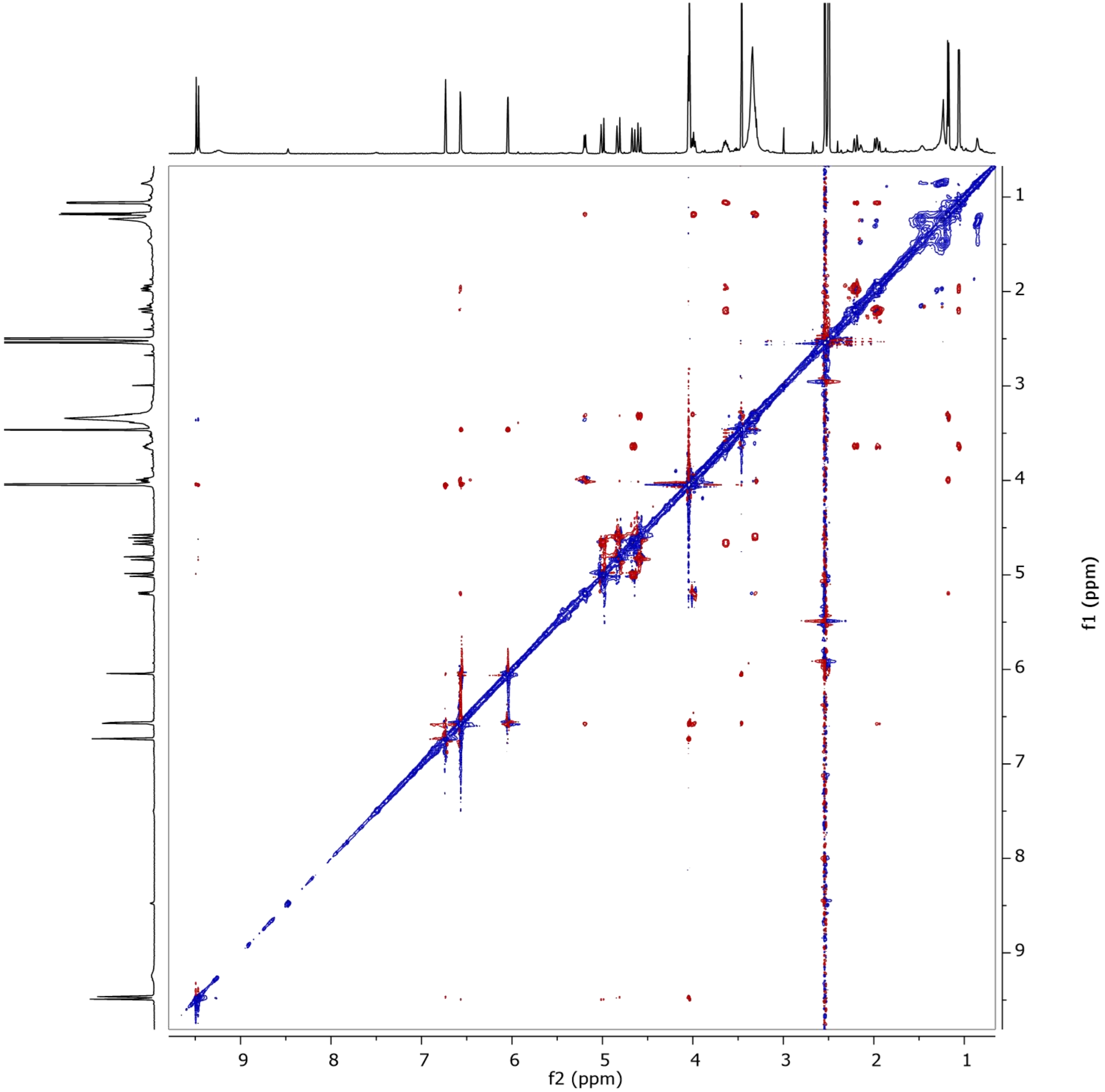

Figure S8. ROESY spectrum of **1** in DMSO-d_6 at 500 MHz.

Generic Display Report

Analysis Info

Analysis Name	S:\DATA\AmaZon\gph22_Kunthide-Gift Phutthacharoen\07-23\MY 07996-F4+F5 R1-F4_diluted_1_1_RA6_01_14766.d	Acquisition Date	06.07.2023 16:27:56
Method	14766.m	Operator	Lab
Sample Name	MY 07996-F4+F5 R1-F4_diluted_1_1	Instrument	amaZon speed
Comment			

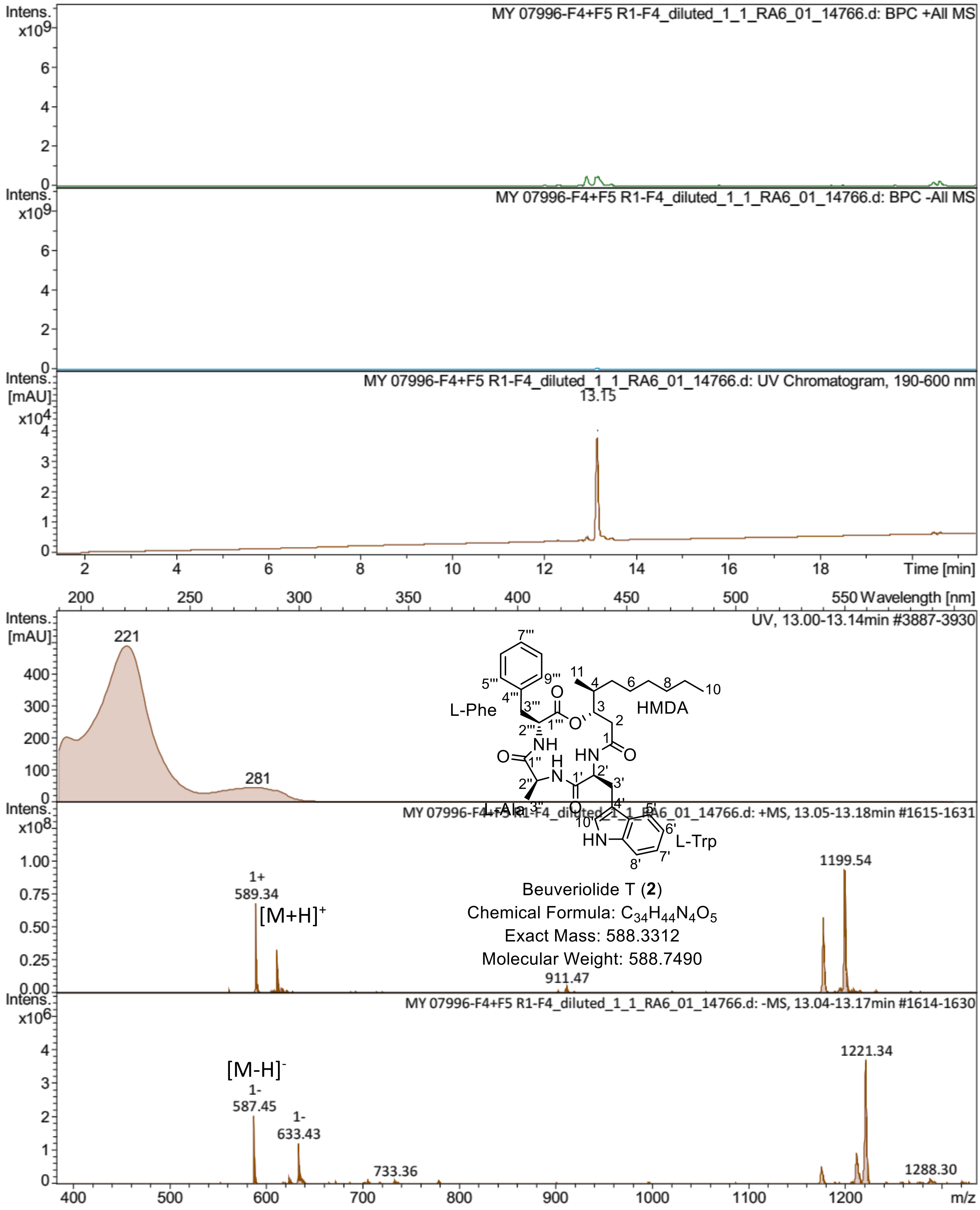

Bruker Compass DataAnalysis 4.4 printed: 19.07.2023 13:02:57 by: sel22 Page 1 of 1

Figure S9. LR-ESI-MS of **2**.

Display Report

Analysis Info

Analysis Name	S:\DATA\MaXis\GPH22_Gift_Kunthide_Phutthacharoen\23_07\23_07_20\MY 007996 F4+F5 R1-F4_56_01_12565.d	Acquisition Date	21.07.2023 00:55:05
Method	pos_säure_10000_screening_ms_100_2500_line.m	Operator	ate06
Sample Name	MY 007996 F4+F5 R1-F4	Instrument	maXis 255552.00037
Comment	Screening01<br>Waters Acquity UPLC BEH C_18 1,7um 2.1x50mm		

Acquisition Parameter

Source Type	ESI	Ion Polarity	Positive	Set Nebulizer	4.0 Bar
Focus	Not active	Set Capillary	4500 V	Set Dry Heater	200 °C
Scan Begin	50 m/z	Set End Plate Offset	-500 V	Set Dry Gas	10.0 l/min
Scan End	2500 m/z	Set Charging Voltage	0 V	Set Divert Valve	Waste
		Set Corona	0 nA	Set APCI Heater	0 °C

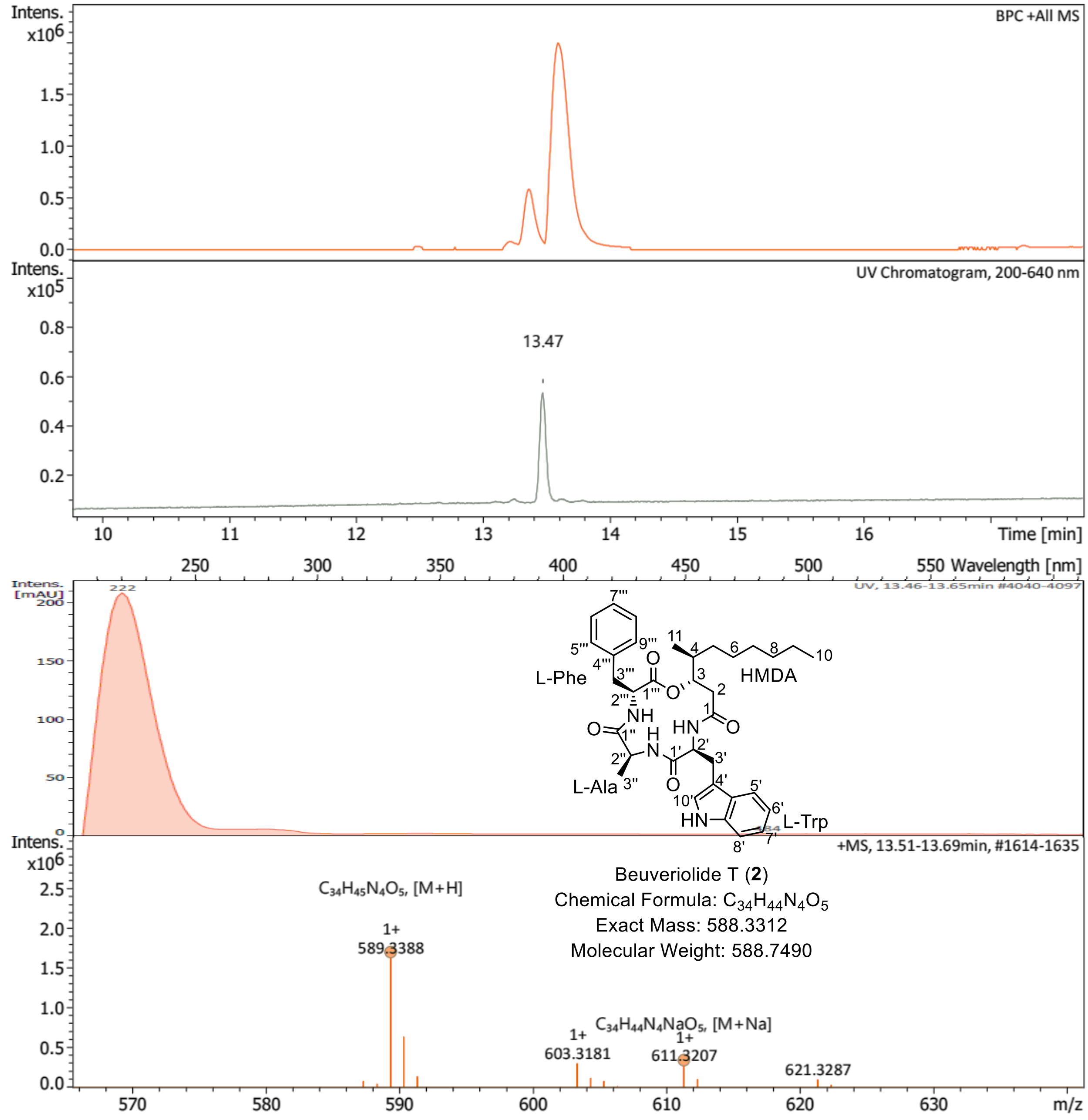

MY 007996 F4+F5 R1-F4_56_01_12565.d

Bruker Compass DataAnalysis 6.1 printed: 15.03.2024 10:57:56 by: sel22 Page 1 of 1

Figure S10. HR-ESI-MS of **2**.

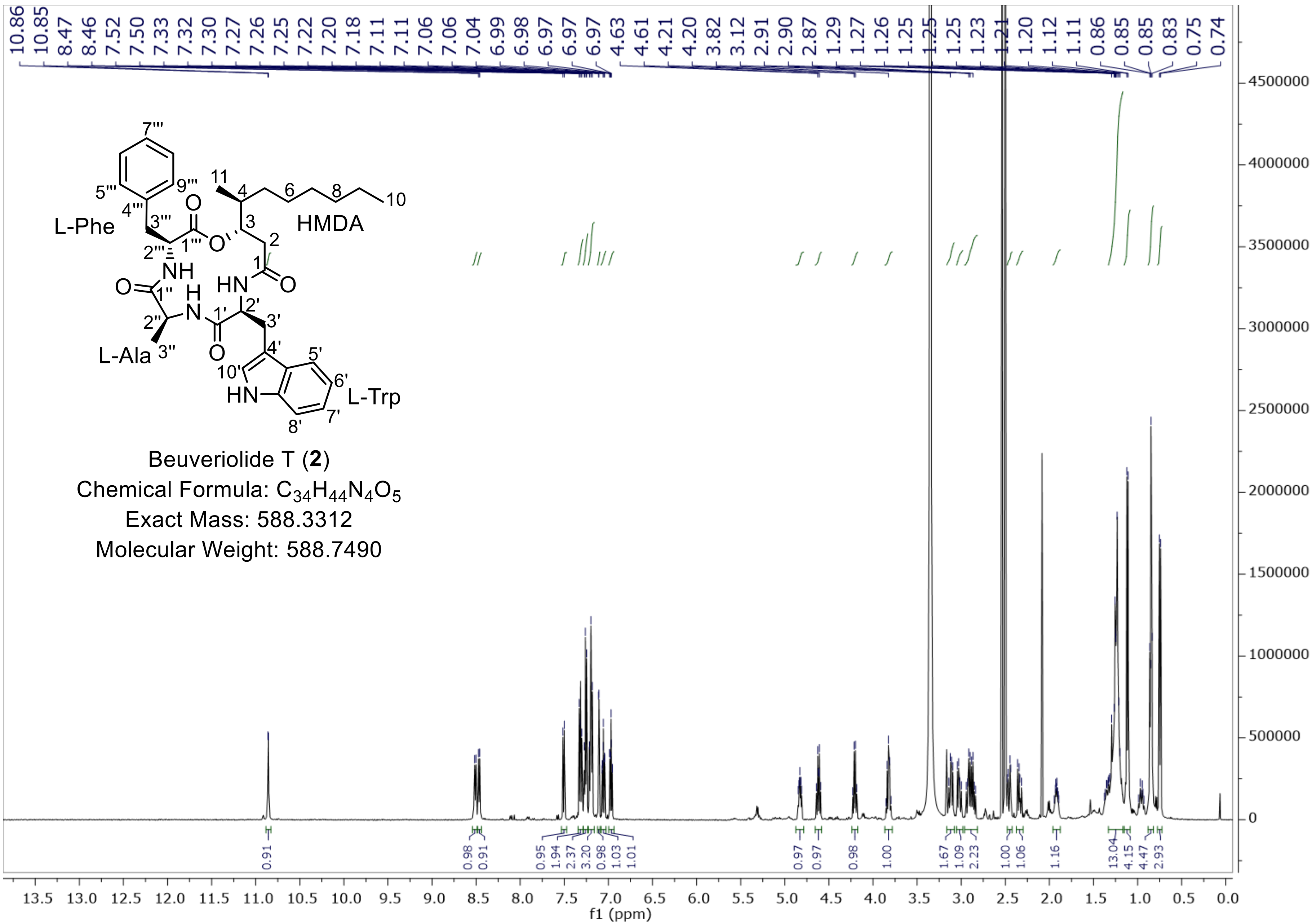

Figure S11. ^{1}H NMR spectrum of **2** in DMSO-d_6 at 500 MHz.

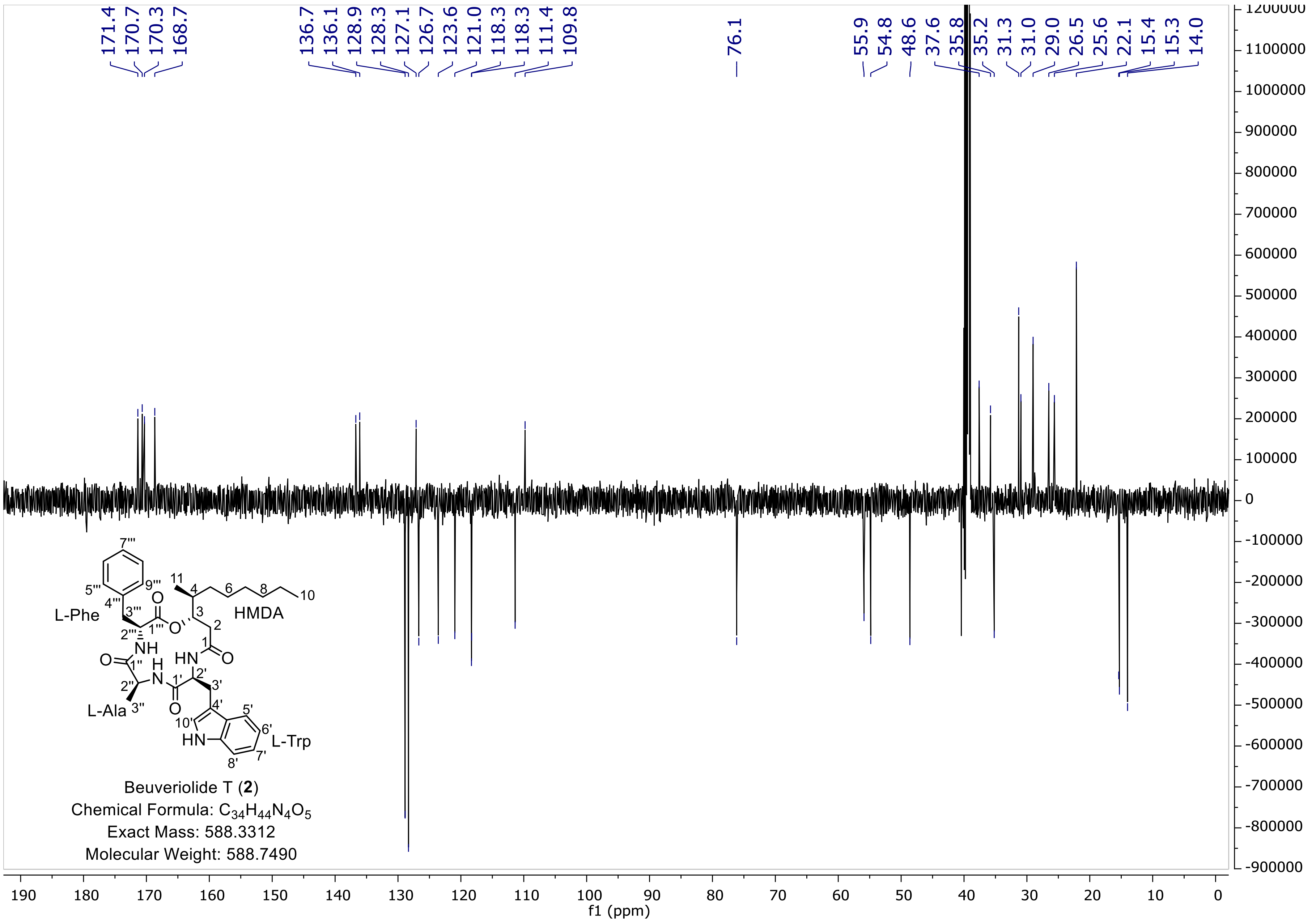

Figure S12. DEPTQ spectrum of **2** in DMSO-d_6 at 125 MHz.

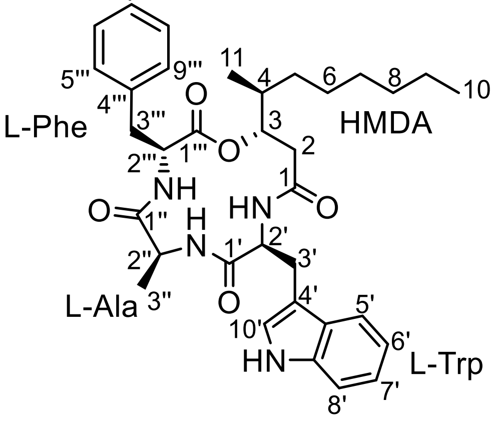

Beuveriolide T (**2**)
Chemical Formula: $C_{34}H_{44}N_4O_5$
Exact Mass: 588.3312
Molecular Weight: 588.7490

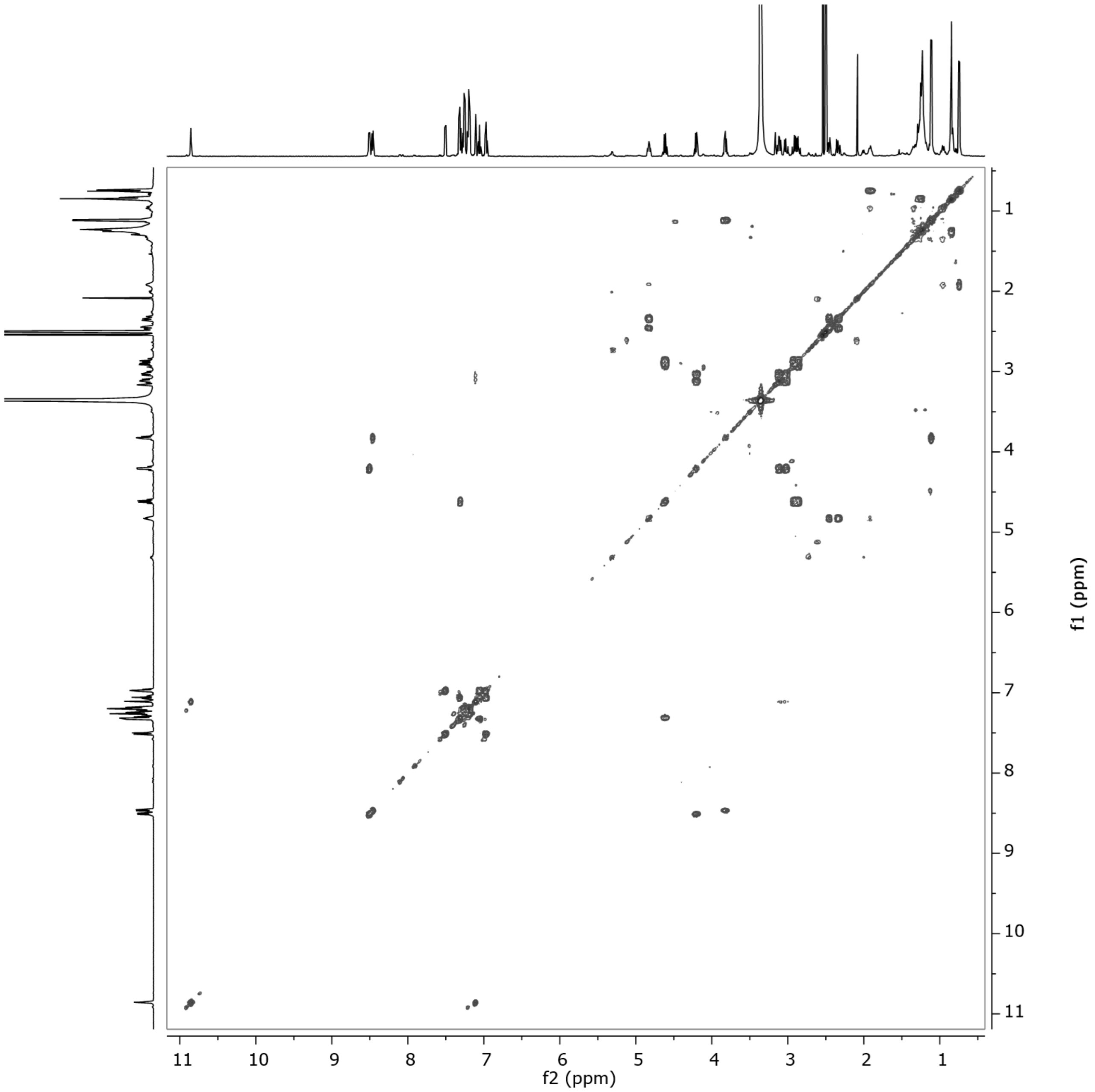

Figure S13. ^{1}H-^{1}H COSY spectrum of **2** in DMSO-d_6 at 500 MHz.

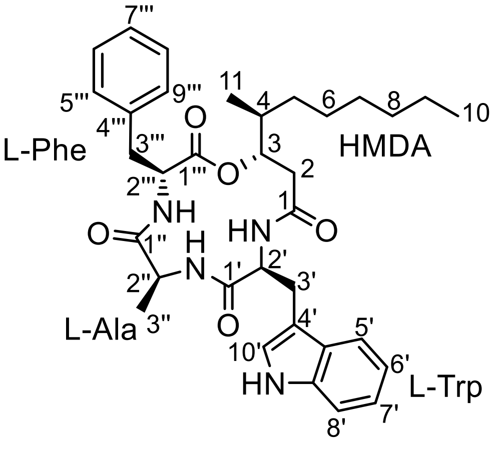

Beuveriolide T (**2**)
Chemical Formula: $C_{34}H_{44}N_4O_5$
Exact Mass: 588.3312
Molecular Weight: 588.7490

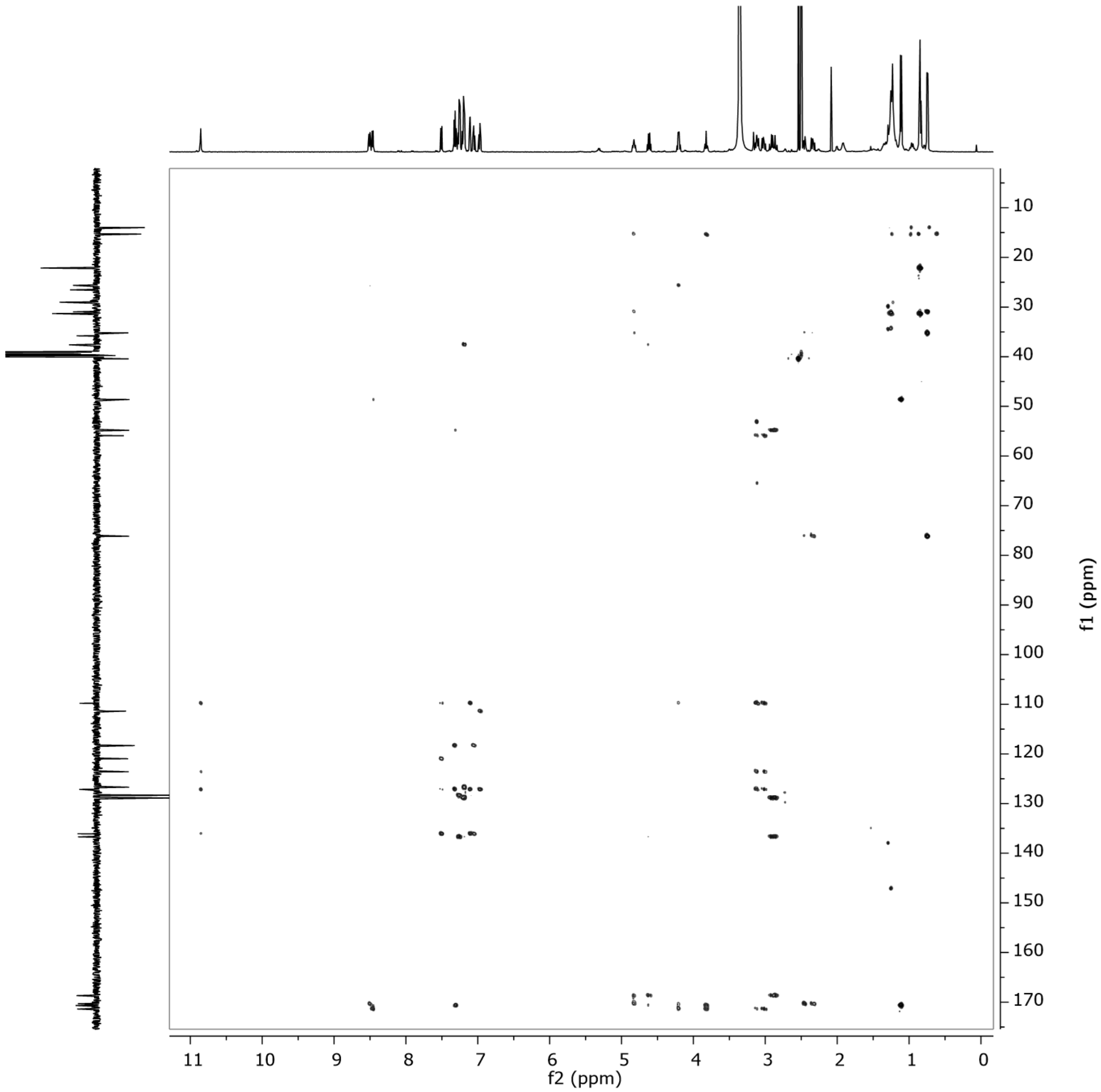

Figure S14. HMBC spectrum of **2** in DMSO-d_6 at 500 MHz.

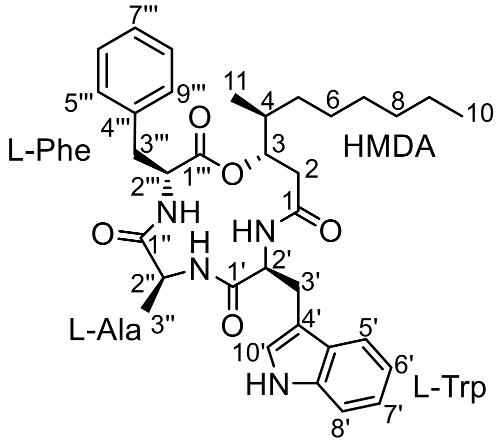

Beuveriolide T (**2**)
Chemical Formula: $C_{34}H_{44}N_4O_5$
Exact Mass: 588.3312
Molecular Weight: 588.7490

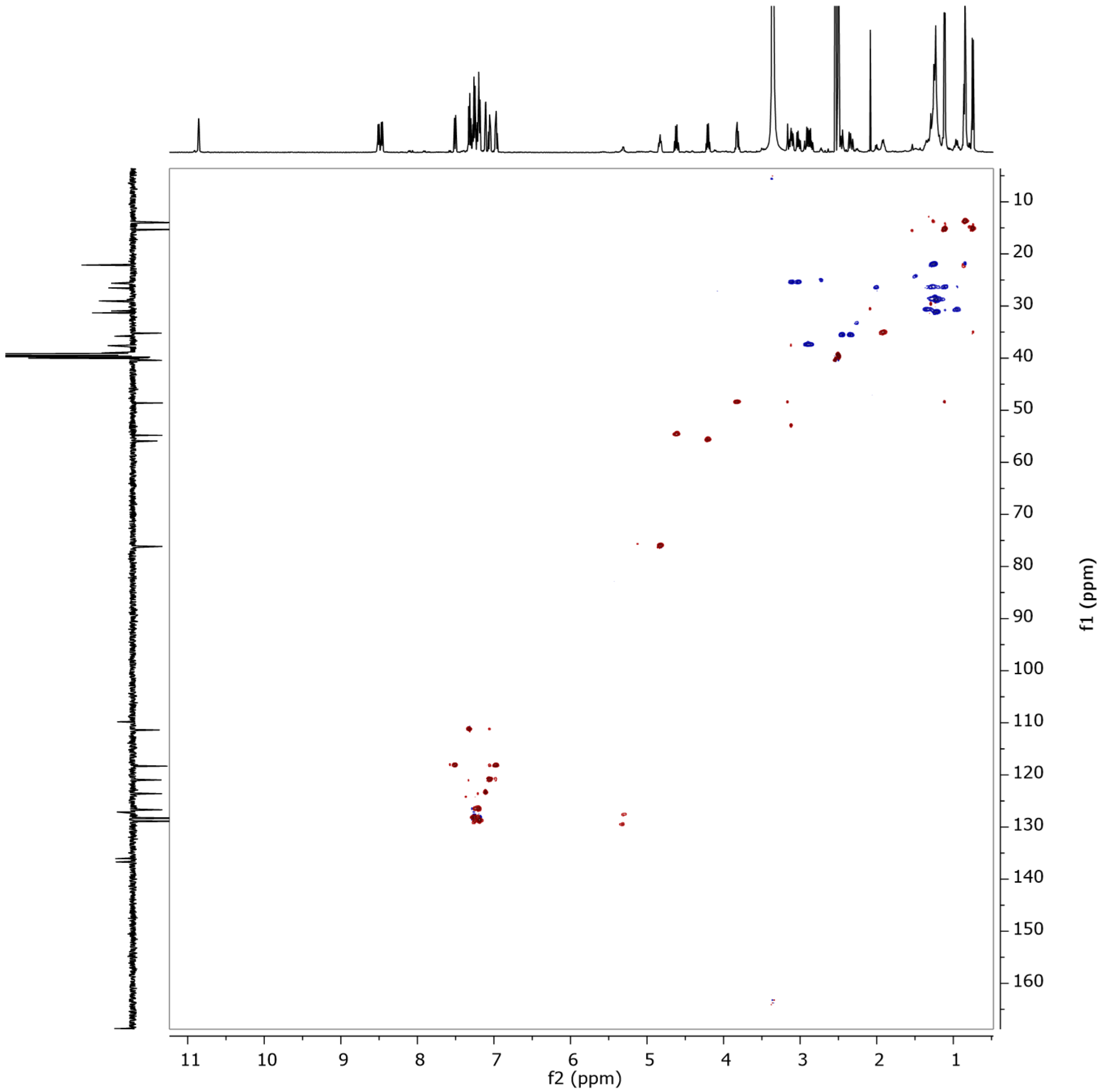

Figure S15. HSQC spectrum of **2** in DMSO-d_6 at 500 MHz.

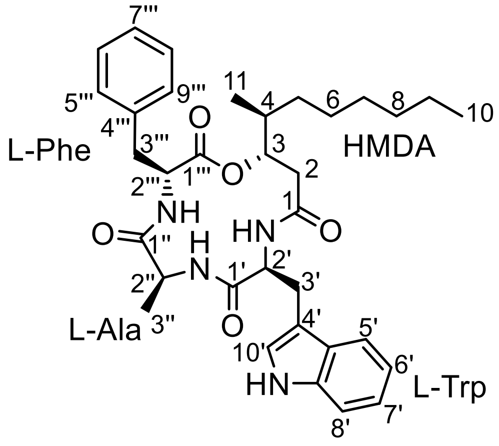

Beuveriolide T (**2**)
Chemical Formula: $C_{34}H_{44}N_4O_5$
Exact Mass: 588.3312
Molecular Weight: 588.7490

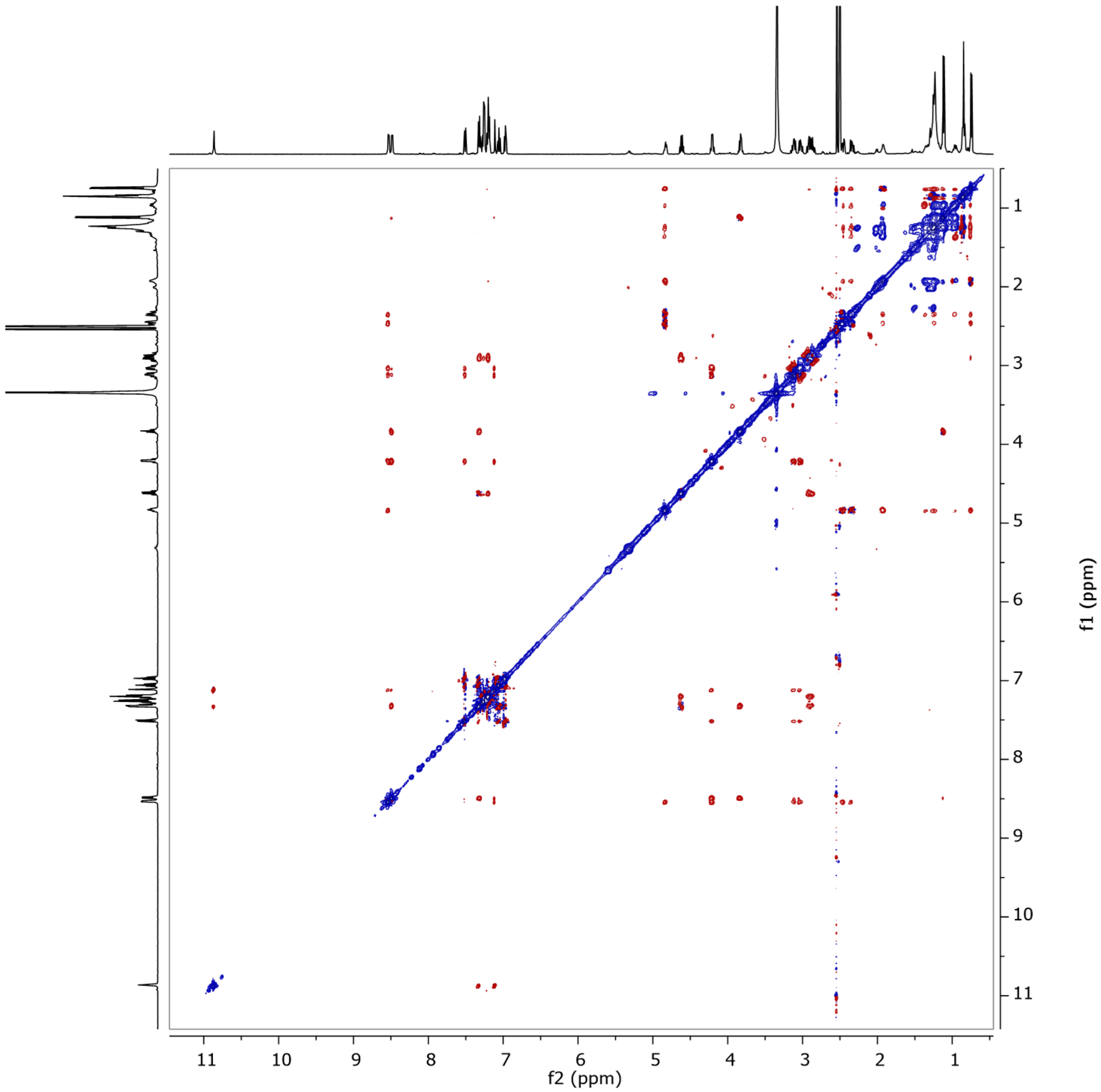

Figure S16. ROESY spectrum of **2** in DMSO-d_6 at 500 MHz.

Generic Display Report

Analysis Info

Analysis Name	S:\DATA\AmaZon\gph22_Kunthide-Gift Phutthacharoen\07-23\MY 07996-F4+F5 R3-F4_RB7_01_14777.d	Acquisition Date	06.07.2023 23:06:16
Method	14777.m	Operator	Lab
Sample Name	MY 07996-F4+F5 R3-F4	Instrument	amaZon speed
Comment			

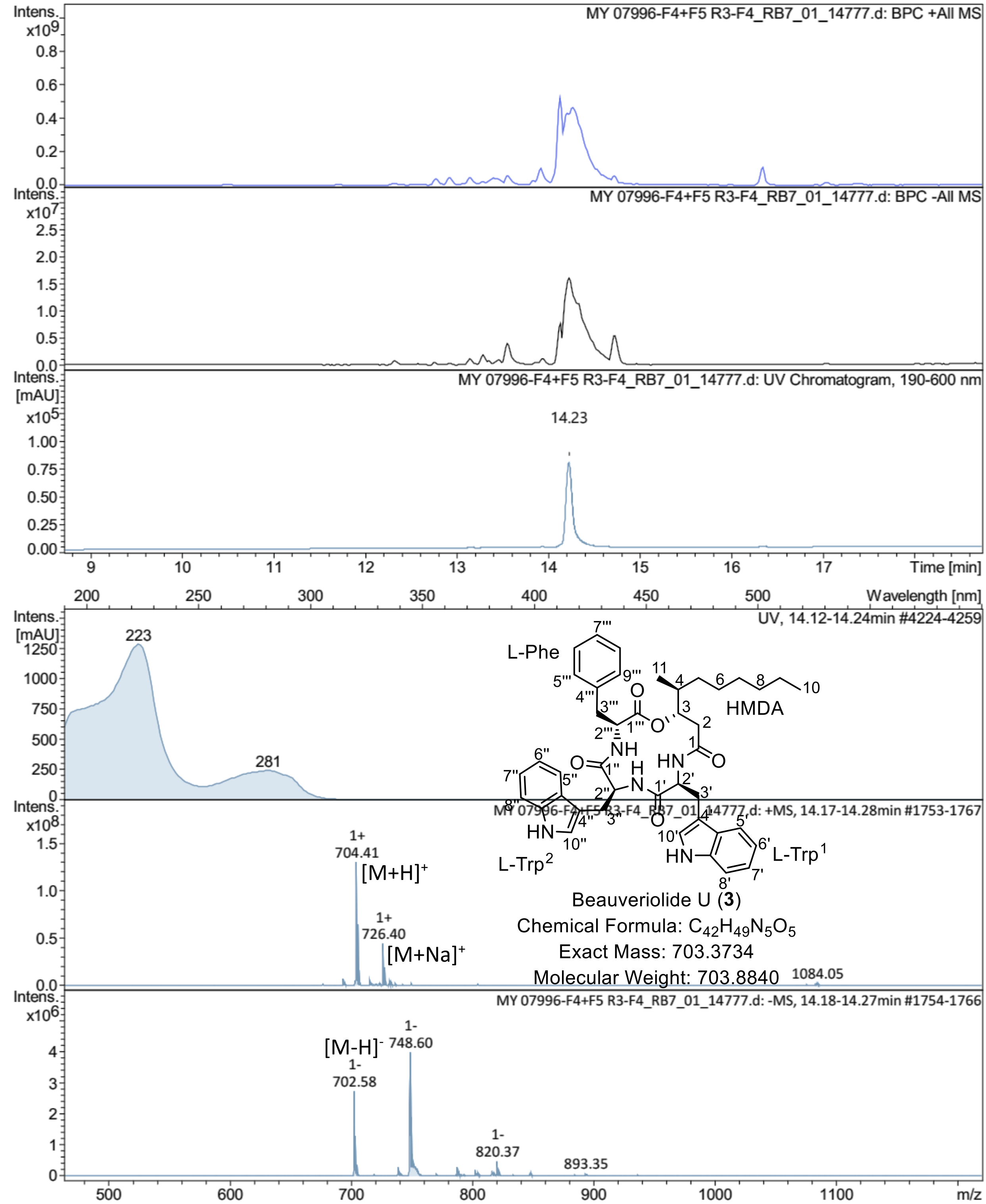

Bruker Compass DataAnalysis 4.4 printed: 19.07.2023 13:06:09 by: sel22 Page 1 of 1

Figure S17. LR-ESI-MS of **3**.

Display Report

Analysis Info

Analysis Name	S:\DATA\MaXis\GPH22_Gift_Kunthide_Phutthacharoen\23_07\23_07_20\MY 007996 F4+F5 R3-F4_57_01_12566.d	Acquisition Date	21.07.2023 01:26:02
Method	pos_säure_10000_screening_ms_100_2500_line.m	Operator	ate06
Sample Name	MY 007996 F4+F5 R3-F4	Instrument	maXis 255552.00037
Comment	Screening01<br>Waters Acquity UPLC BEH C_18 1,7um 2.1x50mm		

Acquisition Parameter

Source Type	ESI	Ion Polarity	Positive	Set Nebulizer	4.0 Bar
Focus	Not active	Set Capillary	4500 V	Set Dry Heater	200 °C
Scan Begin	50 m/z	Set End Plate Offset	-500 V	Set Dry Gas	10.0 l/min
Scan End	2500 m/z	Set Charging Voltage	0 V	Set Divert Valve	Waste
		Set Corona	0 nA	Set APCI Heater	0 °C

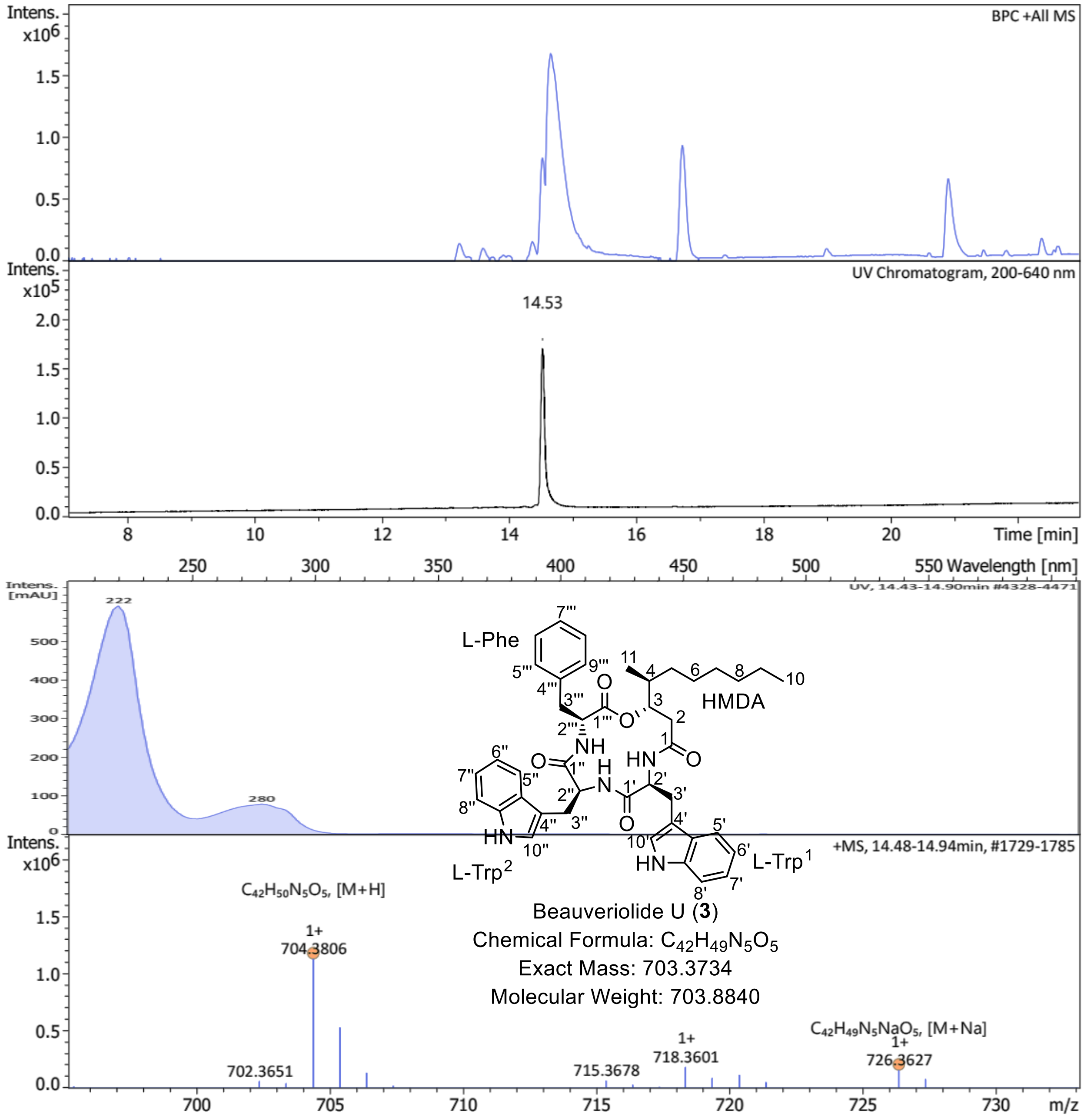

MY 007996 F4+F5 R3-F4_57_01_12566.d

Bruker Compass DataAnalysis 6.1 printed: 15.03.2024 11:06:00 by: sel22 Page 1 of 1

Figure S18. HR-ESI-MS of **3**.

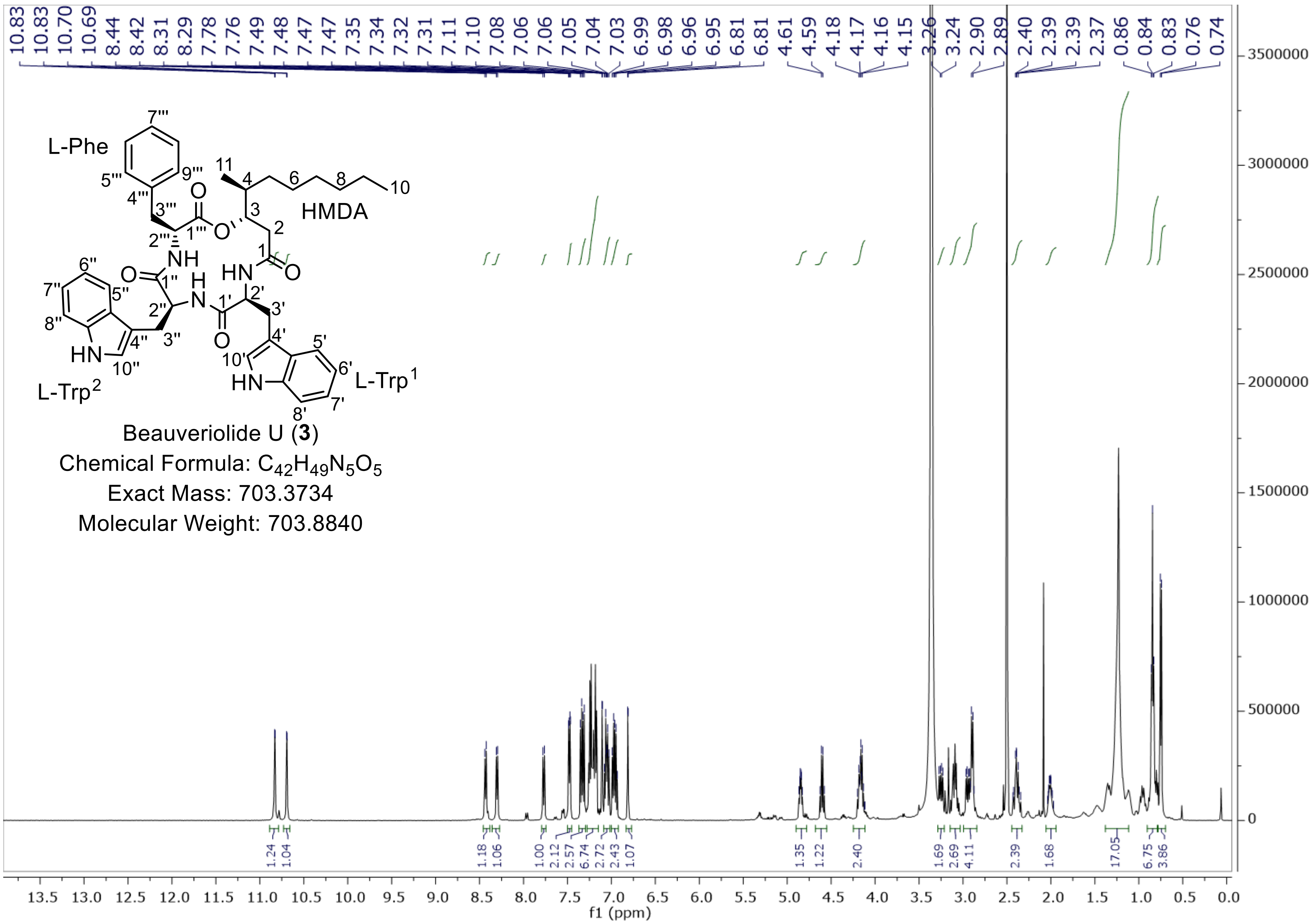

Figure S19. ^{1}H NMR spectrum of **3** in DMSO-d_6 at 500 MHz.

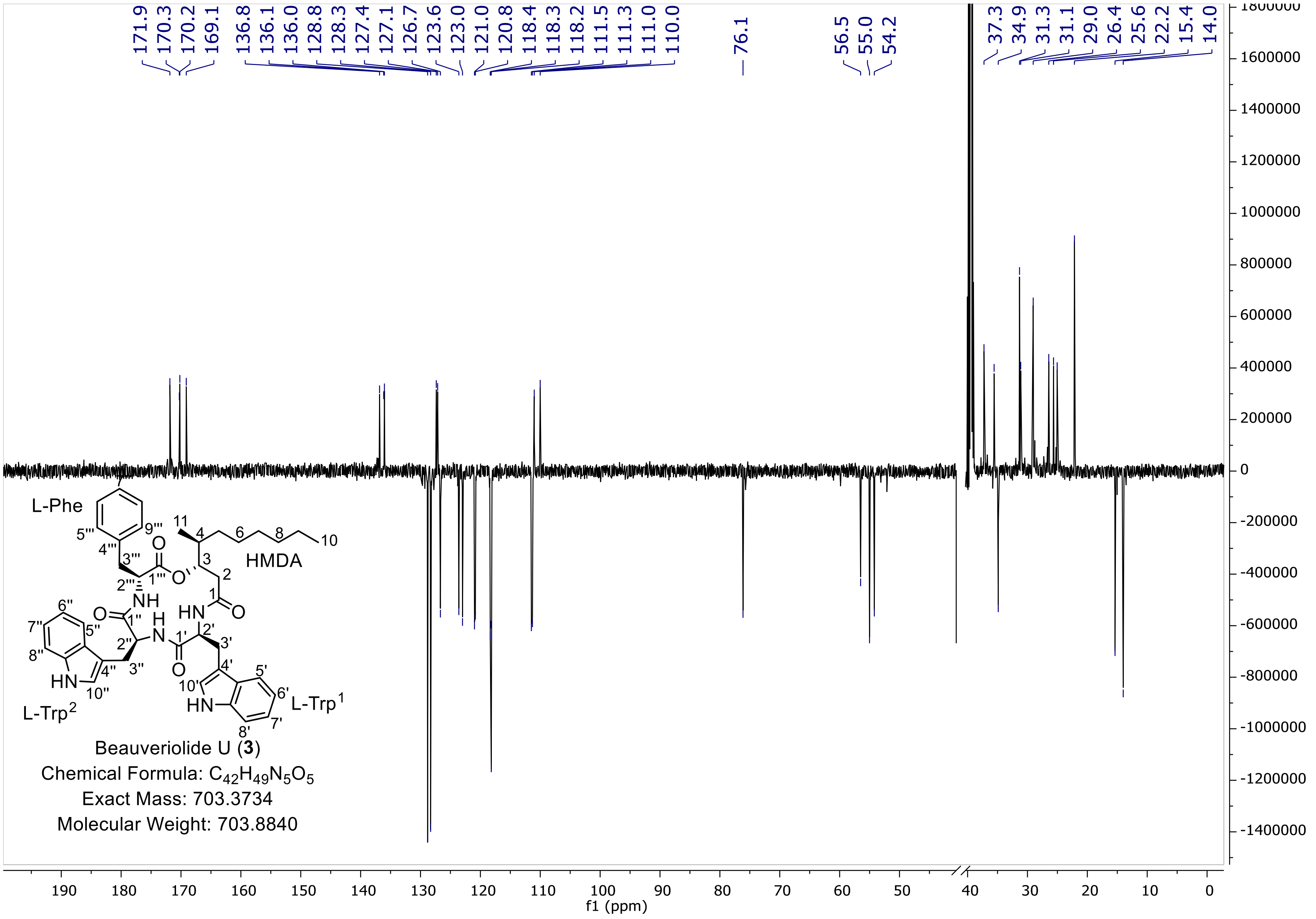

Figure S20. DEPTQ spectrum of **3** in DMSO-d_6 at 125 MHz.

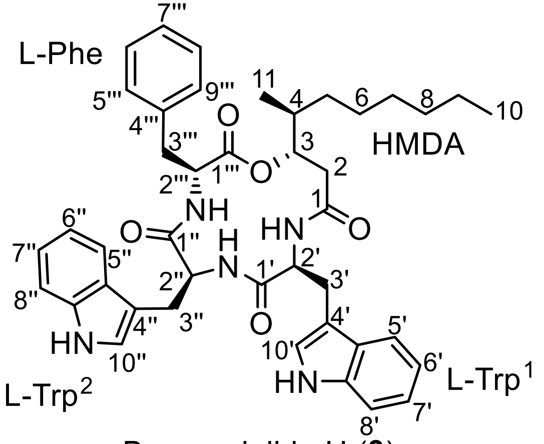

Beauveriolide U (**3**)
Chemical Formula: $C_{42}H_{49}N_5O_5$
Exact Mass: 703.3734
Molecular Weight: 703.8840

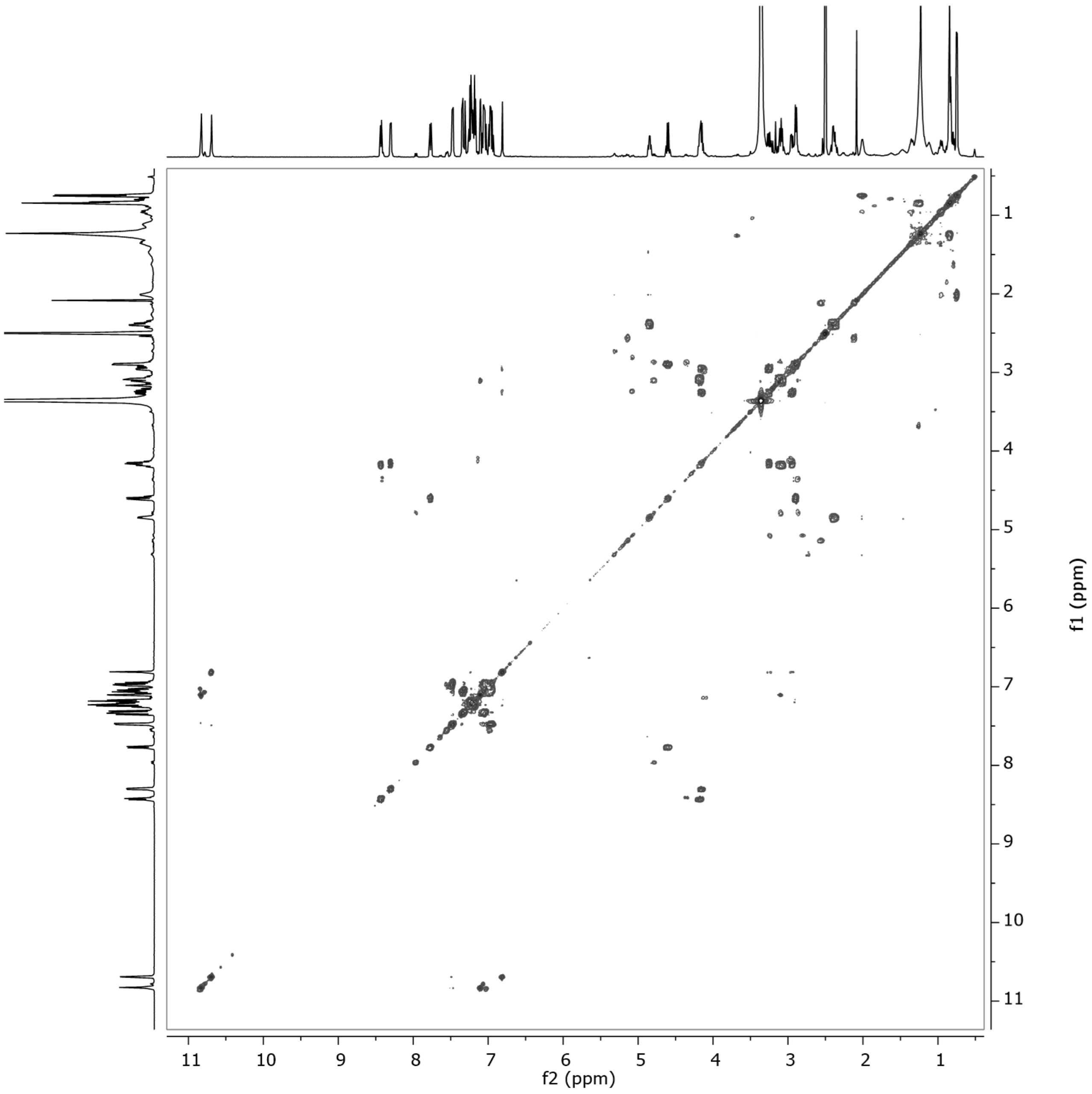

Figure S21. ^1H-^1H COSY spectrum of **3** in DMSO-d_6 at 500 MHz.

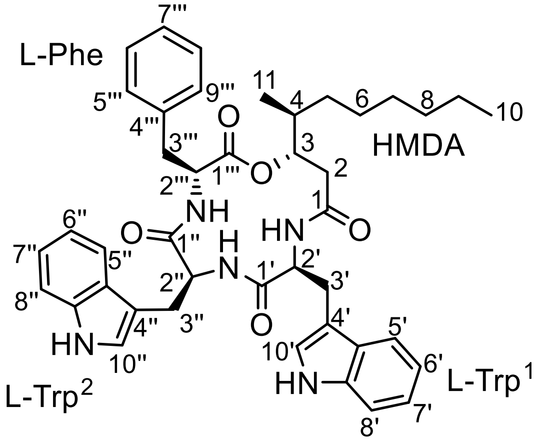

Beauveriolide U (**3**)
Chemical Formula: $C_{42}H_{49}N_5O_5$
Exact Mass: 703.3734
Molecular Weight: 703.8840

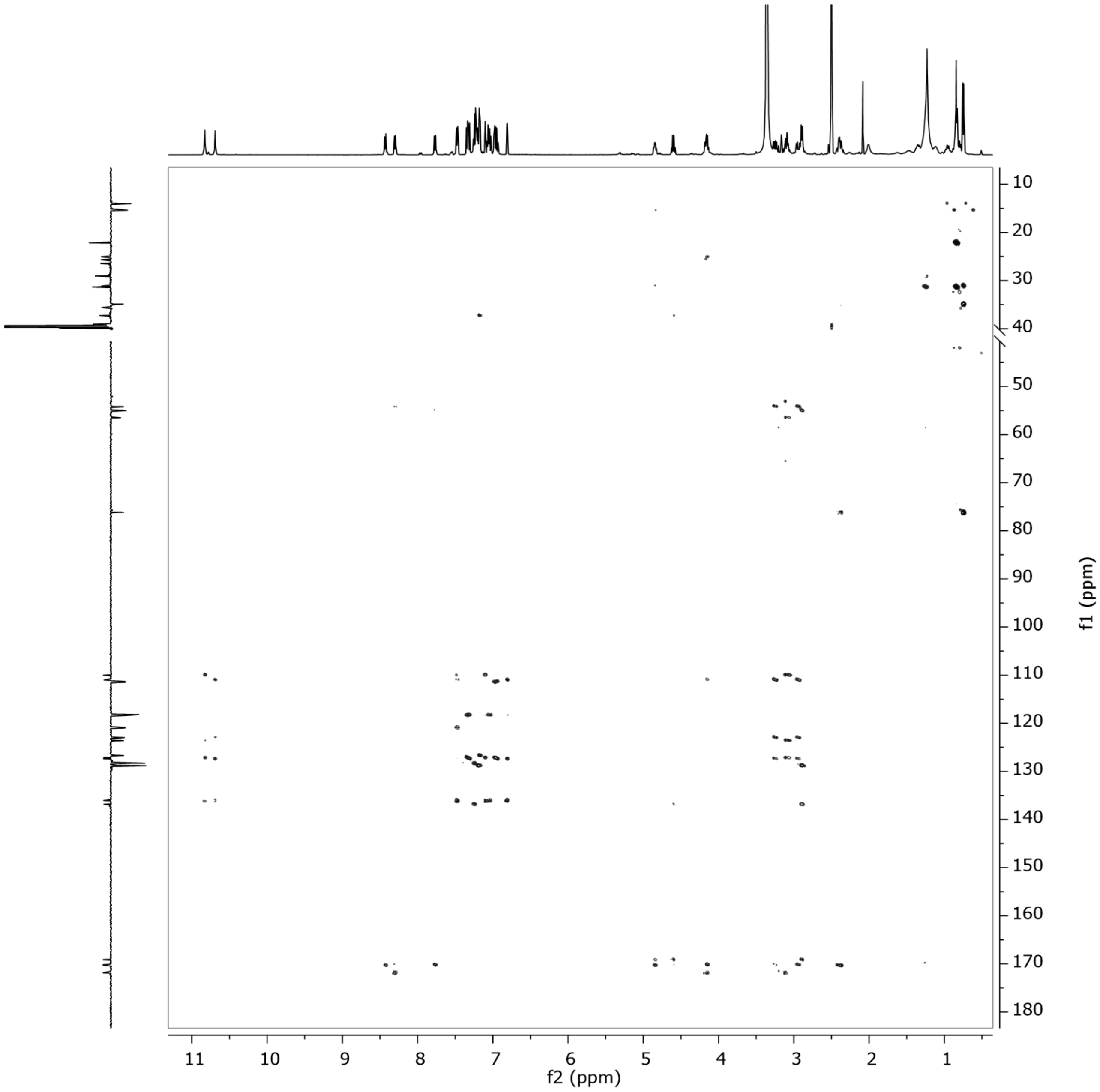

Figure S22. HMBC spectrum of **3** in DMSO-d_6 at 500 MHz.

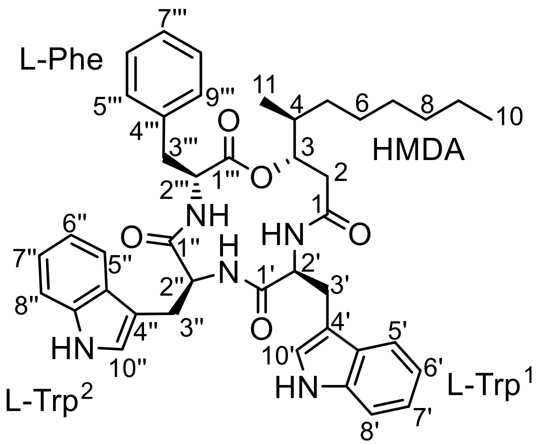

Beauveriolide U (**3**)

Chemical Formula: $C_{42}H_{49}N_5O_5$

Exact Mass: 703.3734

Molecular Weight: 703.8840

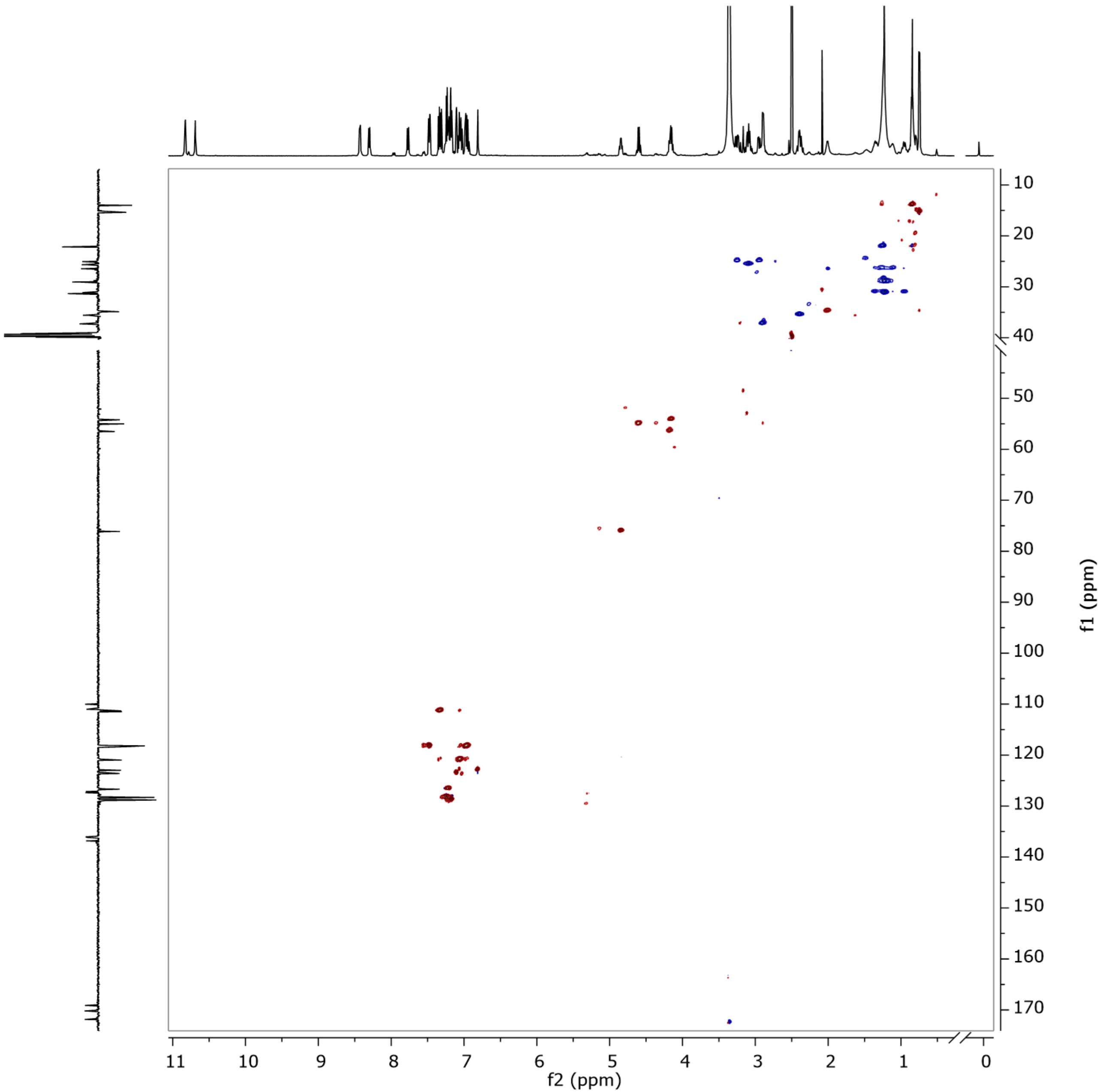

Figure S23. HSQC spectrum of **3** in DMSO-d_6 at 500 MHz.

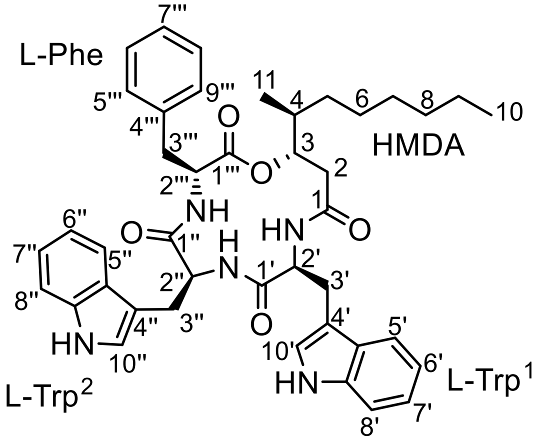

Beauveriolide U (**3**)
Chemical Formula: $C_{42}H_{49}N_5O_5$
Exact Mass: 703.3734
Molecular Weight: 703.8840

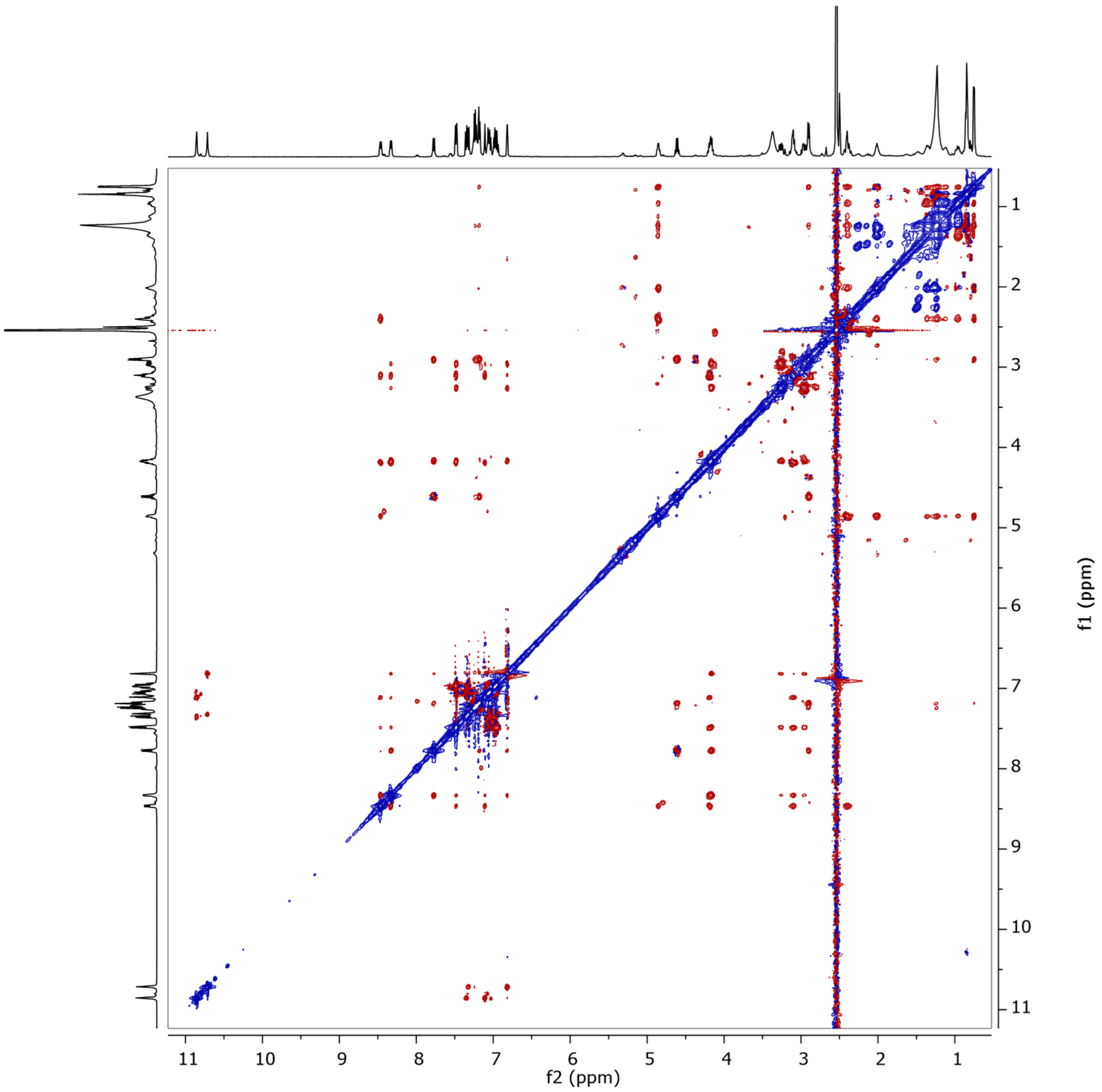

Figure S24. ROESY spectrum of **3** in DMSO-d_6 at 700 MHz.

Display Report

Analysis Info

		Acquisition Date	06.07.2023 14:35:20
Analysis Name	S:\DATA\AmaZon\gph22_Kunthide-Gift Phutthacharoen\07-23\MY 07996-F4+F5 R1-F3_RA5_01_14762.d		
Method	14762.m	Operator	lab
Sample Name	MY 07996-F4+F5 R1-F3	Instrument	amaZon speed
Comment			

Acquisition Parameter

Ion Source Type	ESI	Ion Polarity	Negative	Alternating Ion Polarity	on
Mass Range Mode	UltraScan	Scan Begin	100 m/z	Scan End	2000 m/z
Accumulation Time	4000 µs	RF Level	100 %	Trap Drive	68.9
SPS Target Mass	1000 m/z	Averages	6 Spectra		

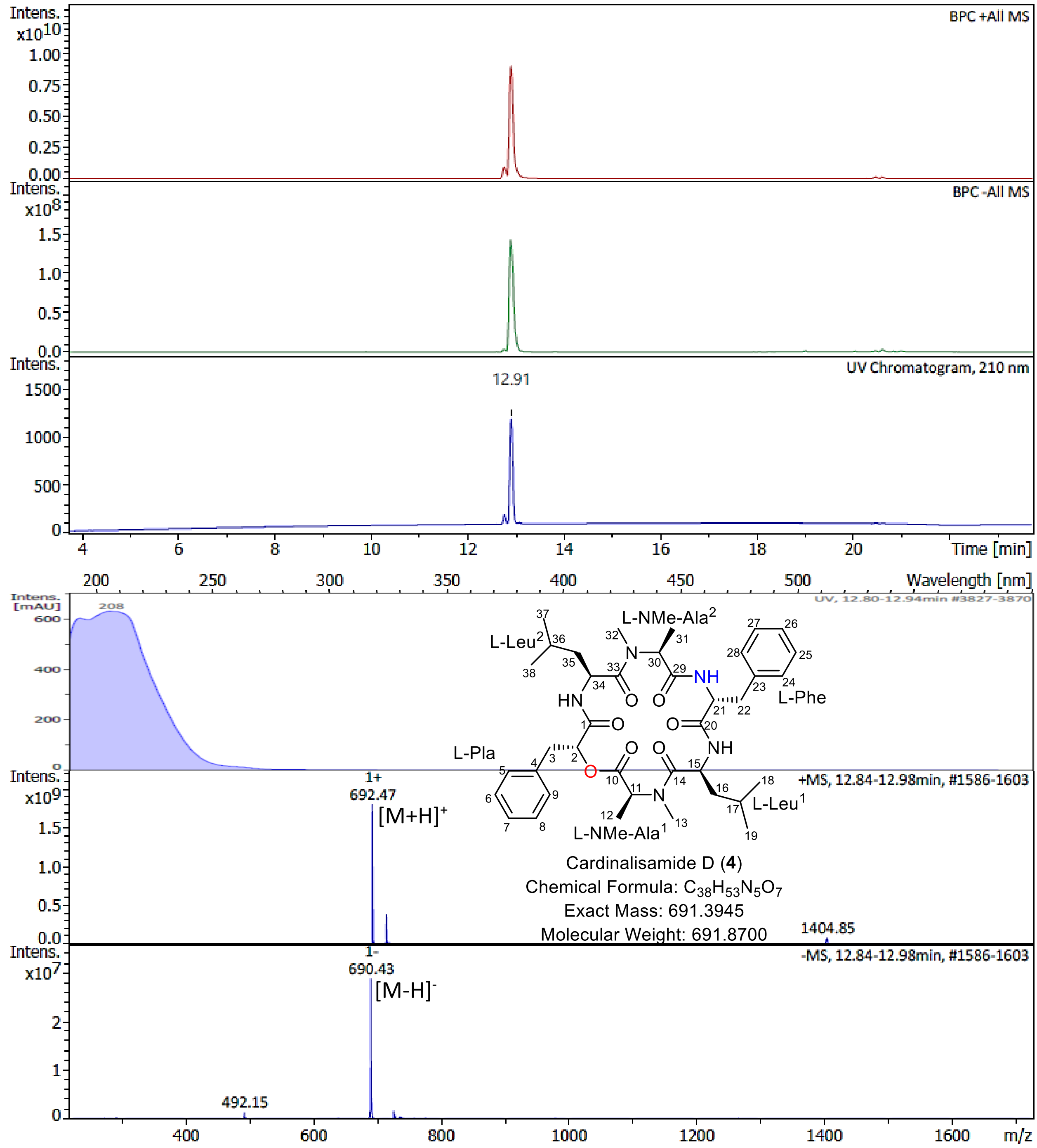

Bruker Compass DataAnalysis 6.1 printed: 27.03.2024 10:40:25 by: sel22 Page 1 of 1

Figure S25. LR-ESI-MS of **4**.

Display Report

Analysis Info

Analysis Name	S:\DATA\MaXis\GPH22_Gift_Kunthide_Phutthacharoen\23_07\MY07996-F3R1-F2_26_01_12253.d	Acquisition Date	10.07.2023 17:19:41
Method	pos_säure_10000_screening_ms_100_2500_line.m	Operator	ate06
Sample Name	MY07996-F3 R1-F2	Instrument	maXis 255552.00037
Comment	Screening01<br>Waters Acquity UPLC BEH C_18 1,7um 2.1x50mm		

Acquisition Parameter

Source Type	ESI	Ion Polarity	Positive	Set Nebulizer	4.0 Bar
Focus	Not active	Set Capillary	4500 V	Set Dry Heater	200 °C
Scan Begin	50 m/z	Set End Plate Offset	-500 V	Set Dry Gas	10.0 l/min
Scan End	2500 m/z	Set Charging Voltage	0 V	Set Divert Valve	Waste
		Set Corona	0 nA	Set APCI Heater	0 °C

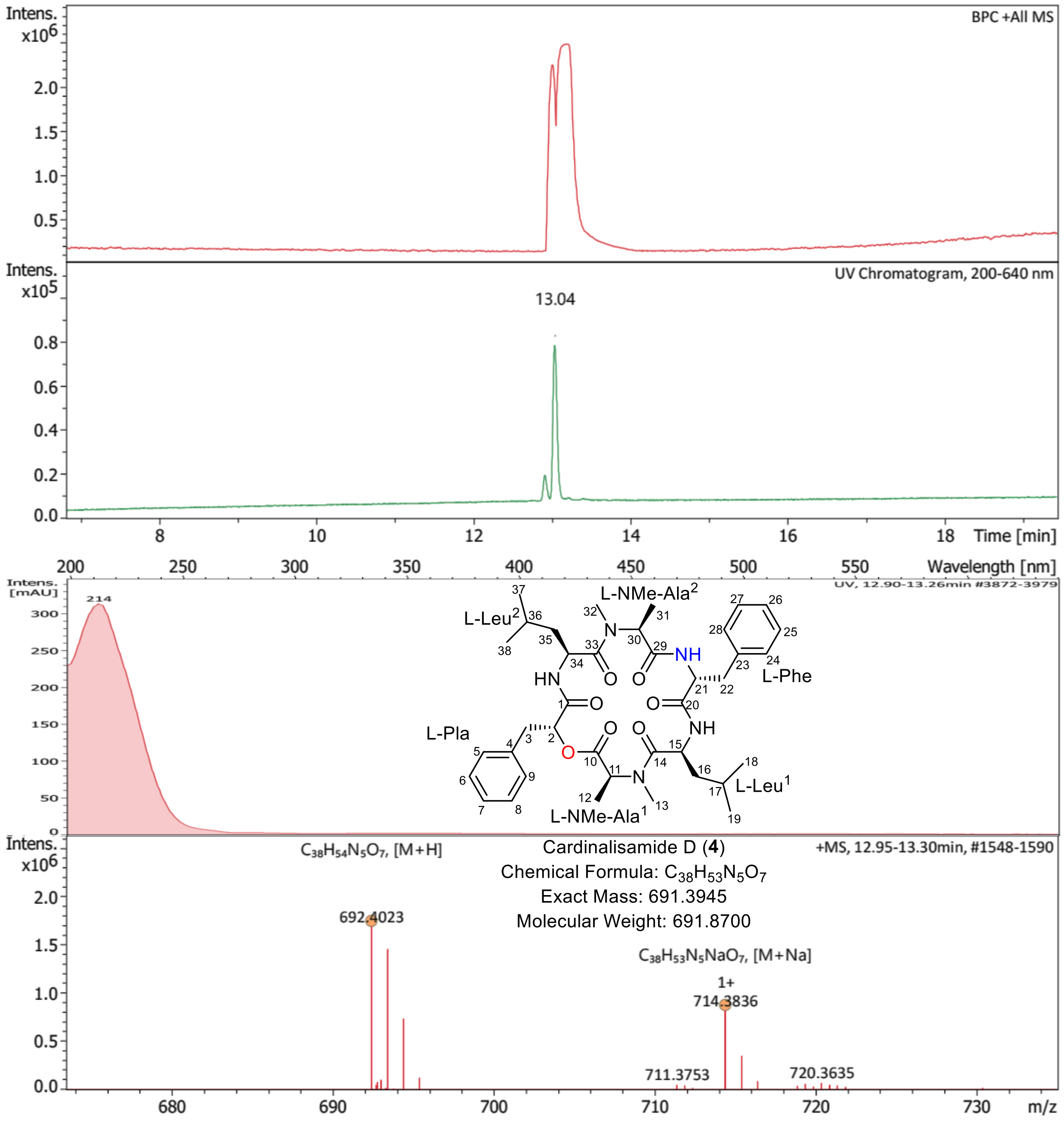

MY07996-F3 R1-F2_26_01_12253.d

Bruker Compass DataAnalysis 6.1 printed: 15.03.2024 10:01:24 by: sel22 Page 1 of 1

Figure S26. HR-ESI-MS of **4**.

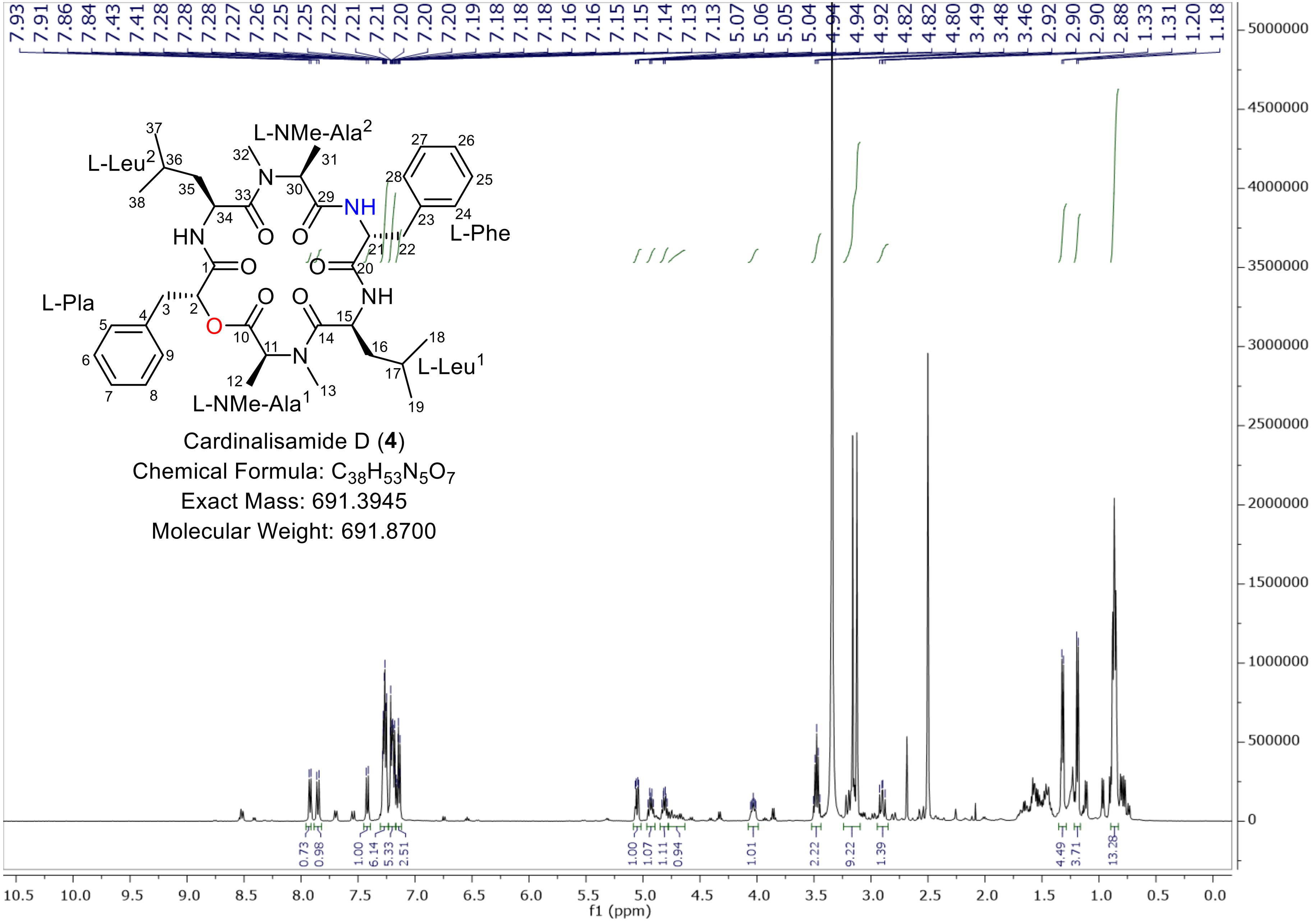

Figure S27. ^{1}H NMR spectrum of **4** in DMSO-d_6 at 500 MHz.

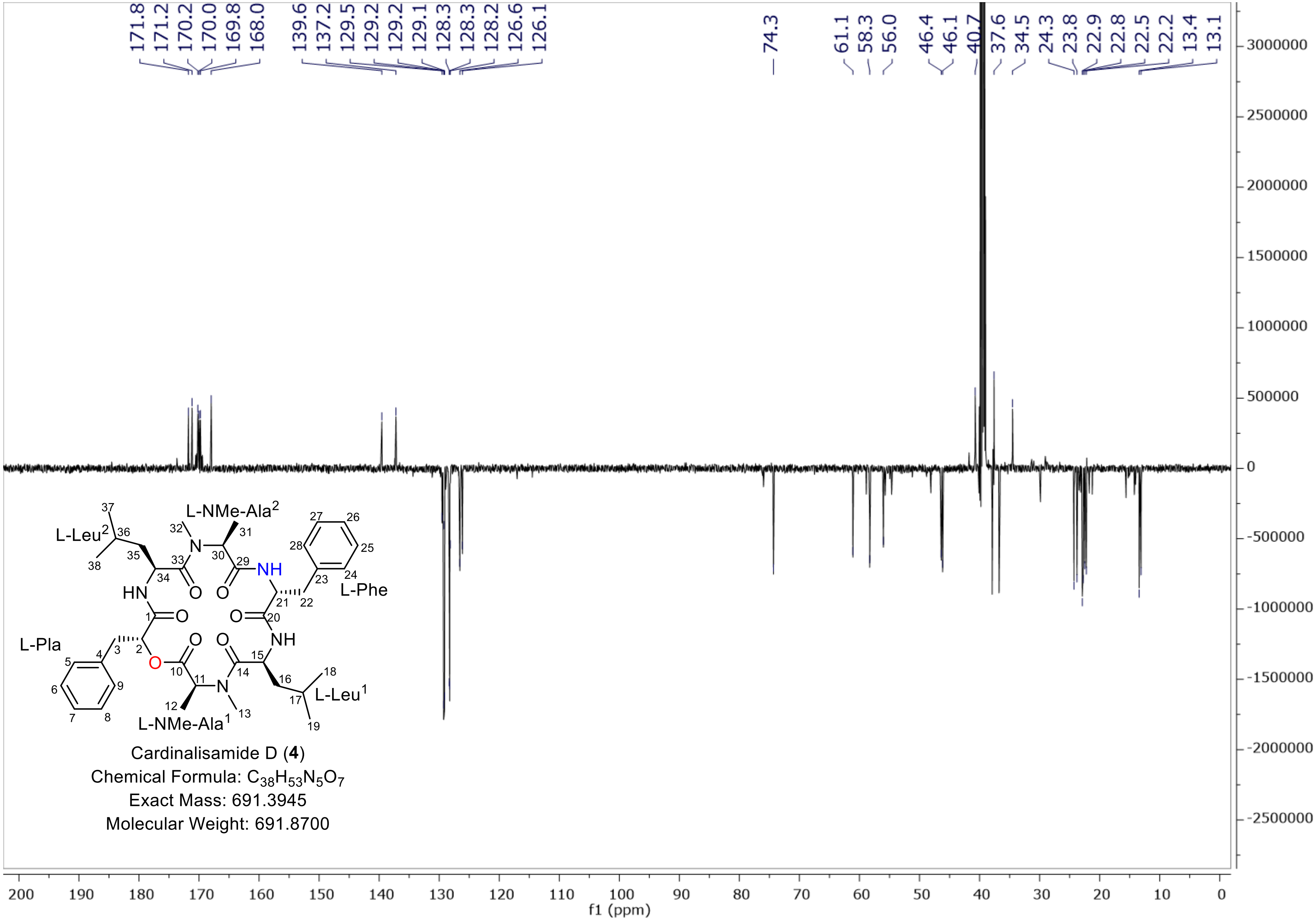

Figure S28. DEPTQ spectrum of **4** in DMSO-d_6 at 125 MHz.

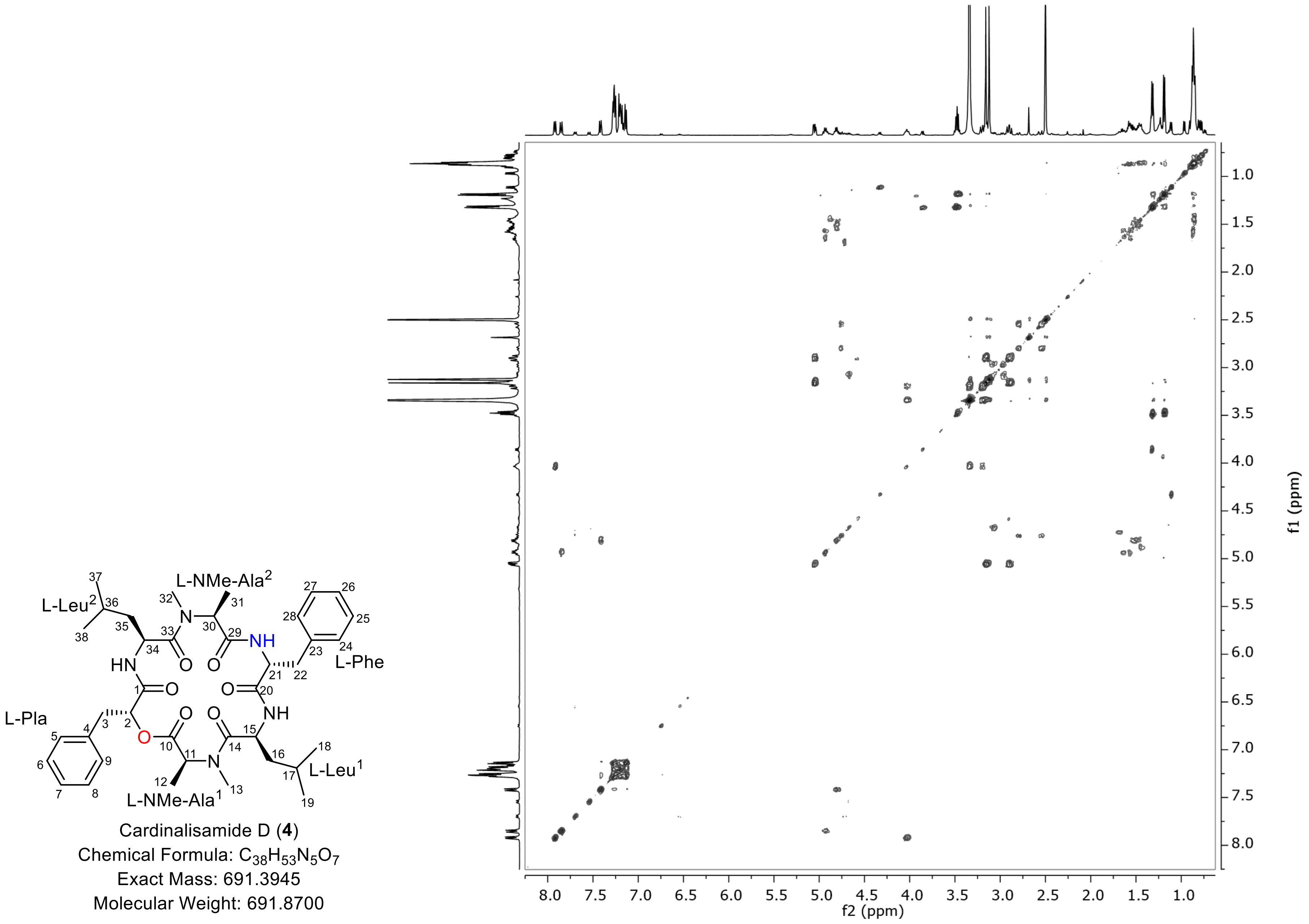

Figure S29. ^{1}H-^{1}H COSY spectrum of **4** in DMSO-d_6 at 500 MHz.

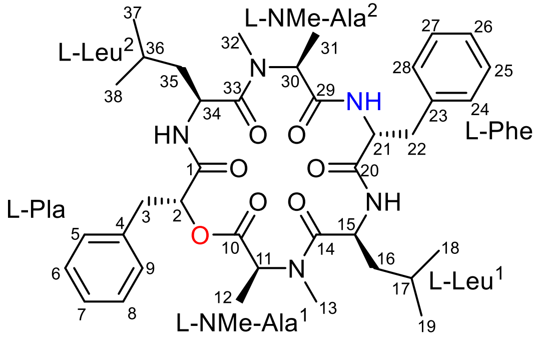

Cardinalisamide D (**4**)
Chemical Formula: $C_{38}H_{53}N_5O_7$
Exact Mass: 691.3945
Molecular Weight: 691.8700

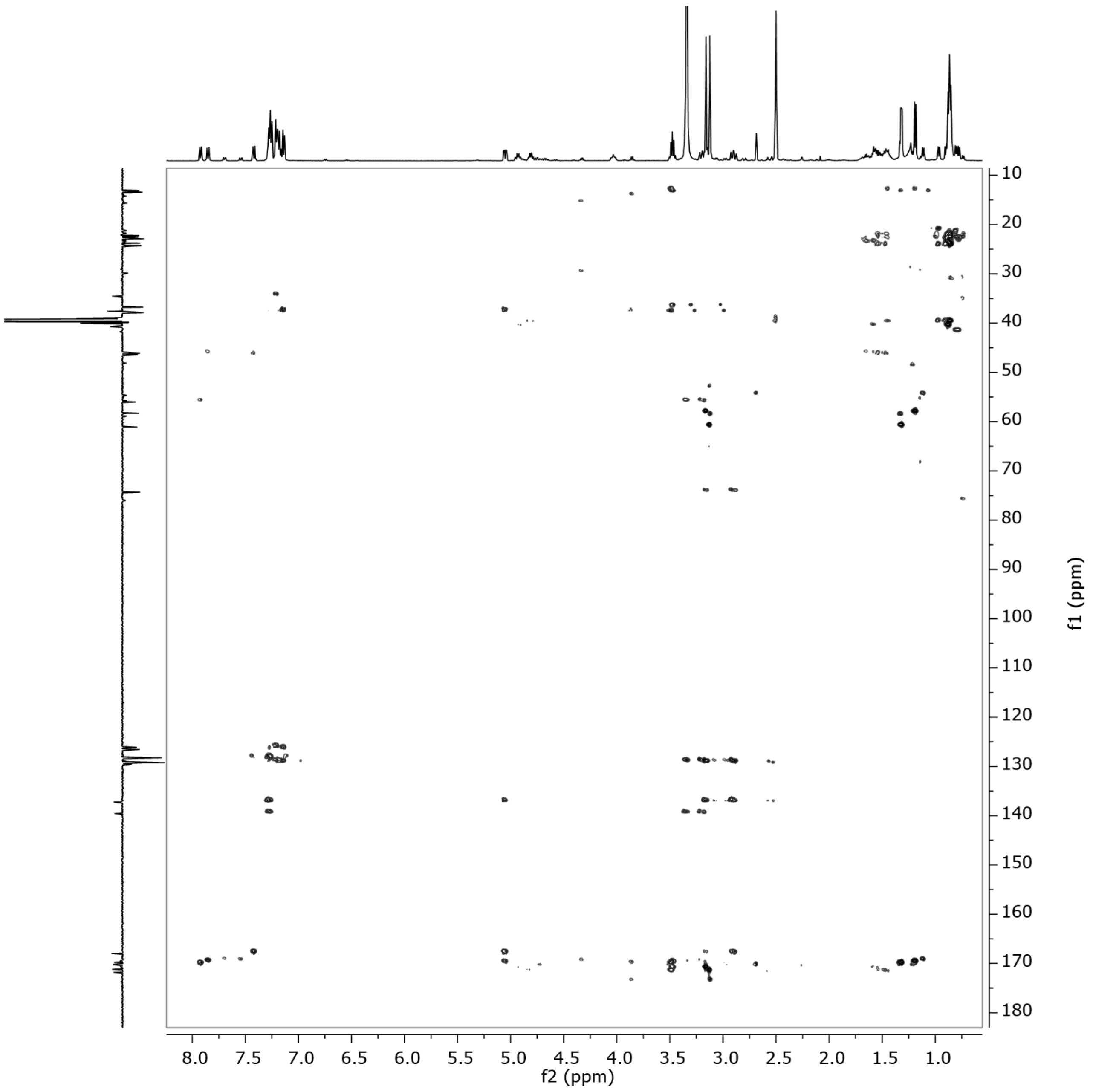

Figure S30. HMBC spectrum of **4** in DMSO-d_6 at 500 MHz.

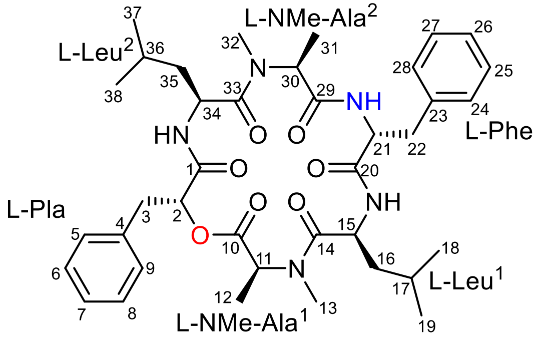

Cardinalisamide D (**4**)
Chemical Formula: $C_{38}H_{53}N_5O_7$
Exact Mass: 691.3945
Molecular Weight: 691.8700

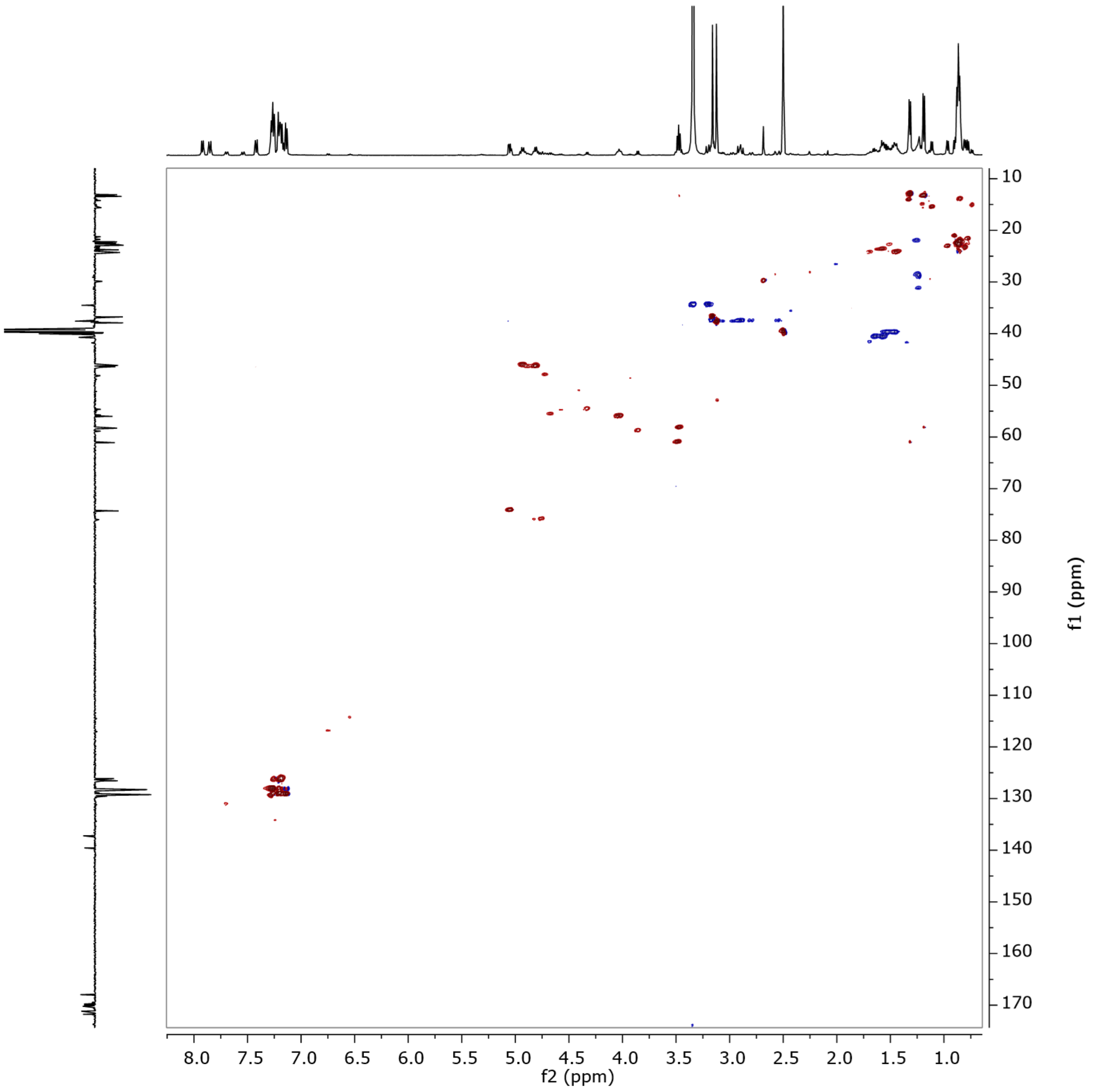

Figure S31. HSQC spectrum of **4** in DMSO-d_6 at 500 MHz.

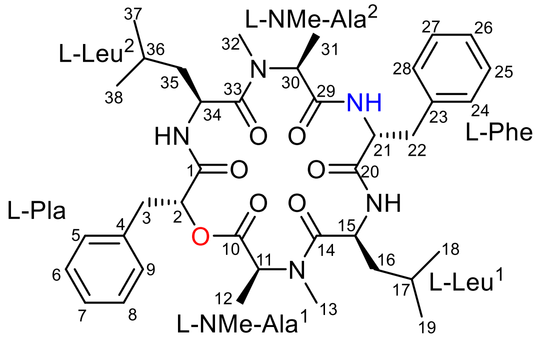

Cardinalisamide D (**4**)

Chemical Formula: $C_{38}H_{53}N_5O_7$

Exact Mass: 691.3945

Molecular Weight: 691.8700

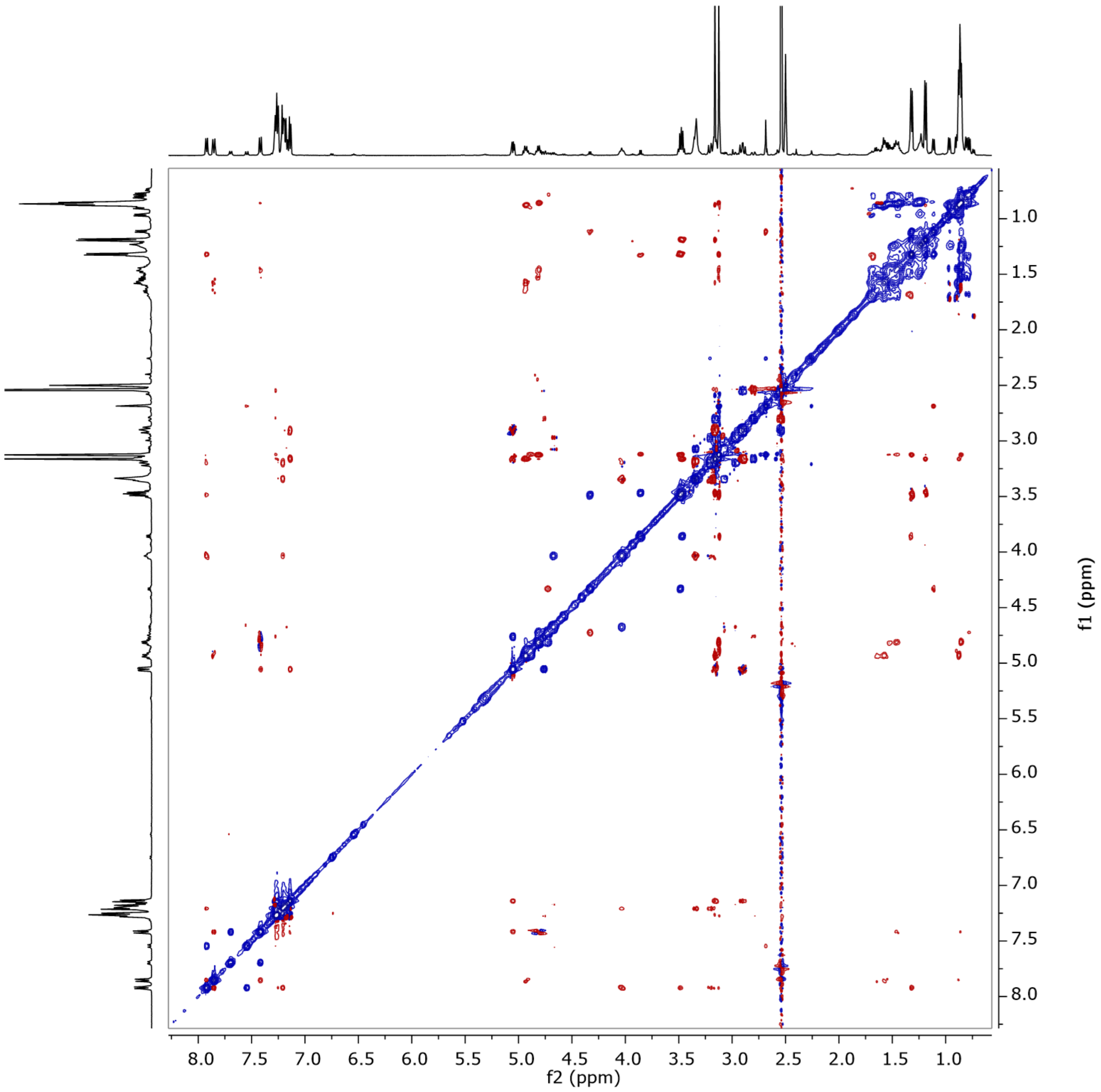

Figure S32. ROESY spectrum of **4** in DMSO-d_6 at 700 MHz.

Generic Display Report

Analysis Info

Analysis Name	S:\DATA\AmaZon\gph22_Kunthide-Gift Phutthacharoen\07-23\MY 07796-F3 R1-F9_RD4_01_14791.d	Acquisition Date	07.07.2023 07:33:03
Method	14791.m	Operator	Lab
Sample Name	MY 07796-F3 R1-F9	Instrument	amaZon speed
Comment			

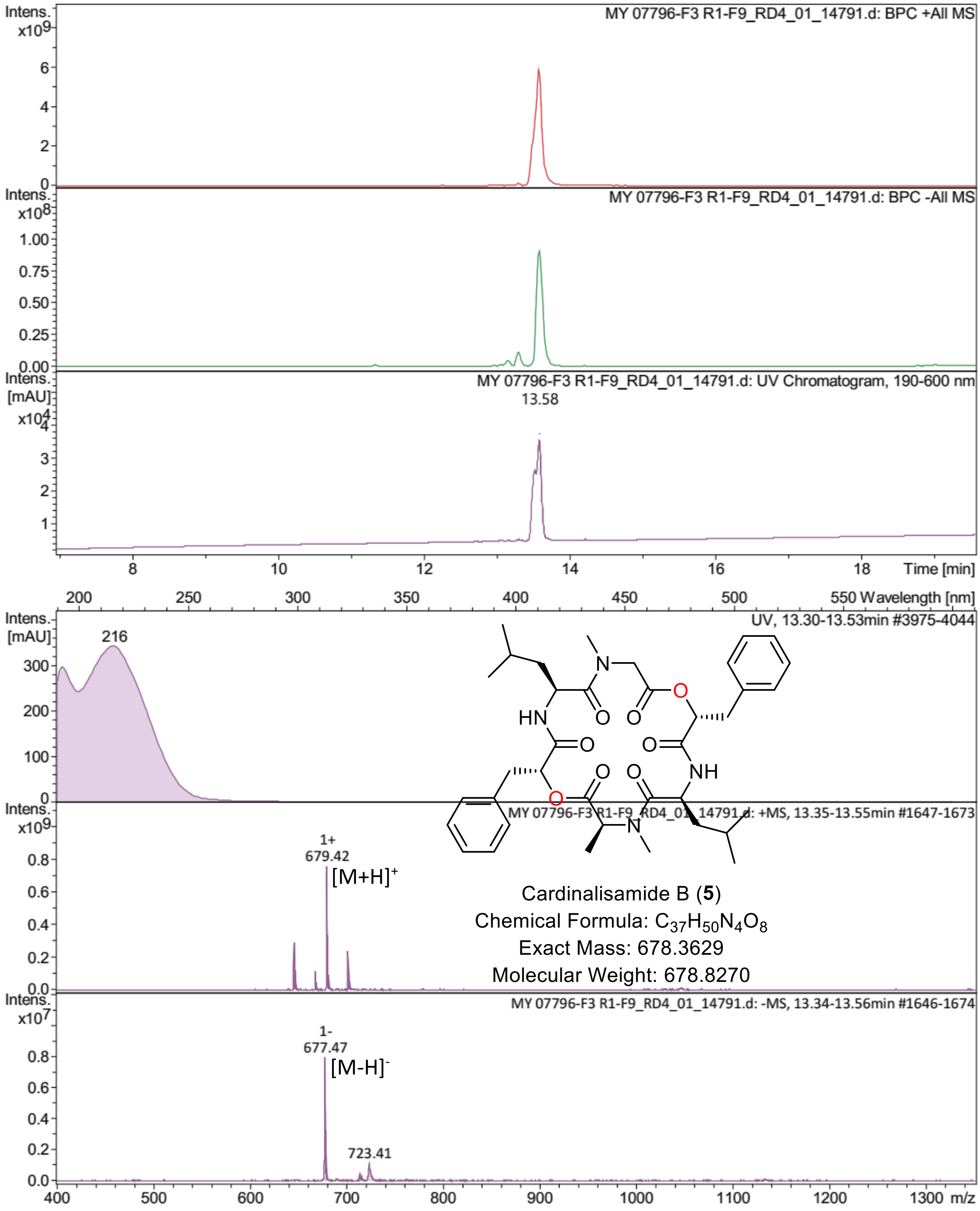

Bruker Compass DataAnalysis 4.4 printed: 19.07.2023 13:12:27 by: sel22 Page 1 of 1

Figure S33. LR-ESI-MS of **5**.

Display Report

Analysis Info

		Acquisition Date	10.07.2023 19:23:36
Analysis Name	S:\DATA\MaXis\GPH22_Gift_Kunthide_Phutthacharoen\23_07\MY07996-F3R1-F10_30_01_12257.d		
Method	pos_säure_10000_screening_ms_100_2500_line.m	Operator	ate06
Sample Name	MY07996-F3 R1-F10	Instrument	maXis
Comment	Screening01<br>Waters Acquity UPLC BEH C_18 1,7um 2.1x50mm		

Acquisition Parameter

Ion Polarity Positive

SPS Target Mass

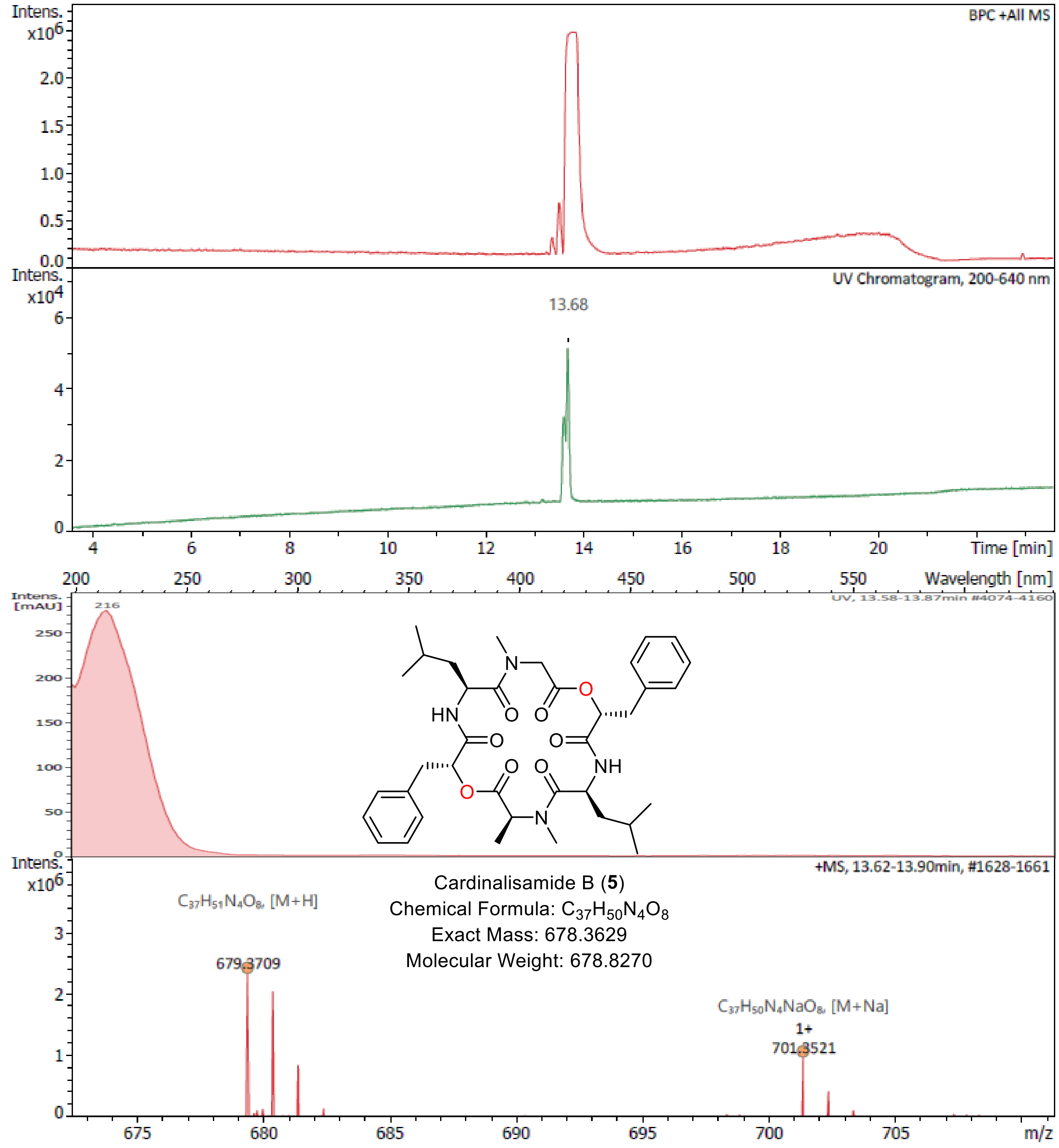

Figure S34. HR-ESI-MS of **5**.

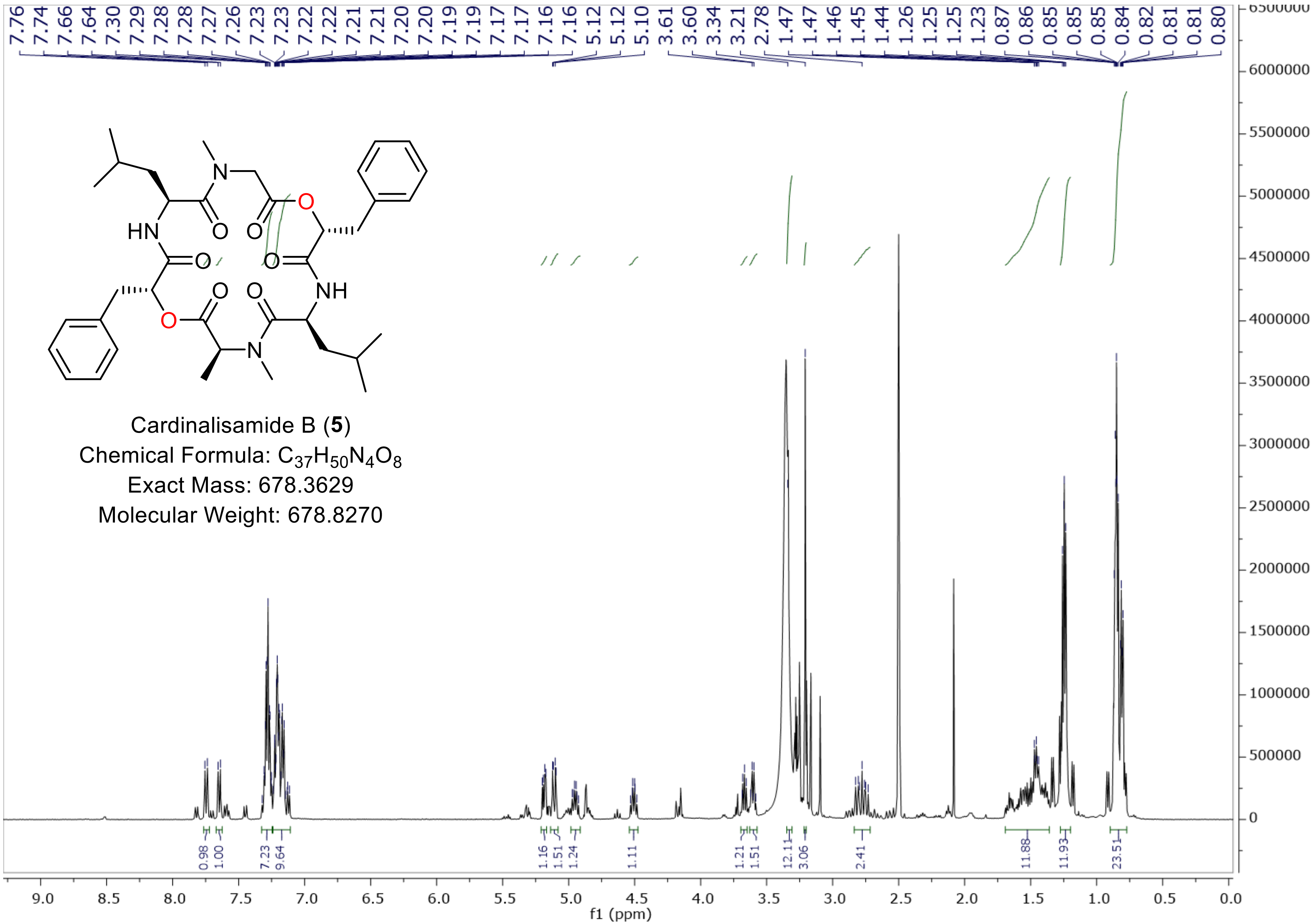

Figure S35. ^{1}H NMR spectrum of **5** in DMSO-d_6 at 500 MHz.

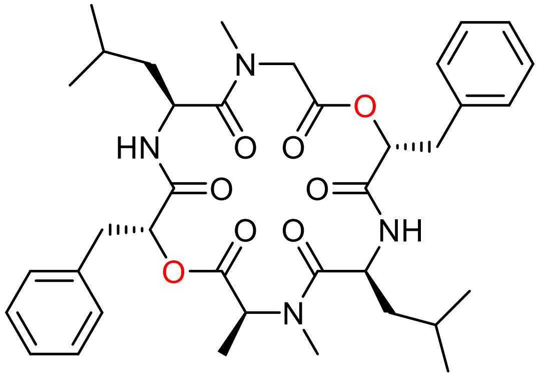

Cardinalisamide B (**5**)
Chemical Formula: $C_{37}H_{50}N_4O_8$
Exact Mass: 678.3629
Molecular Weight: 678.8270

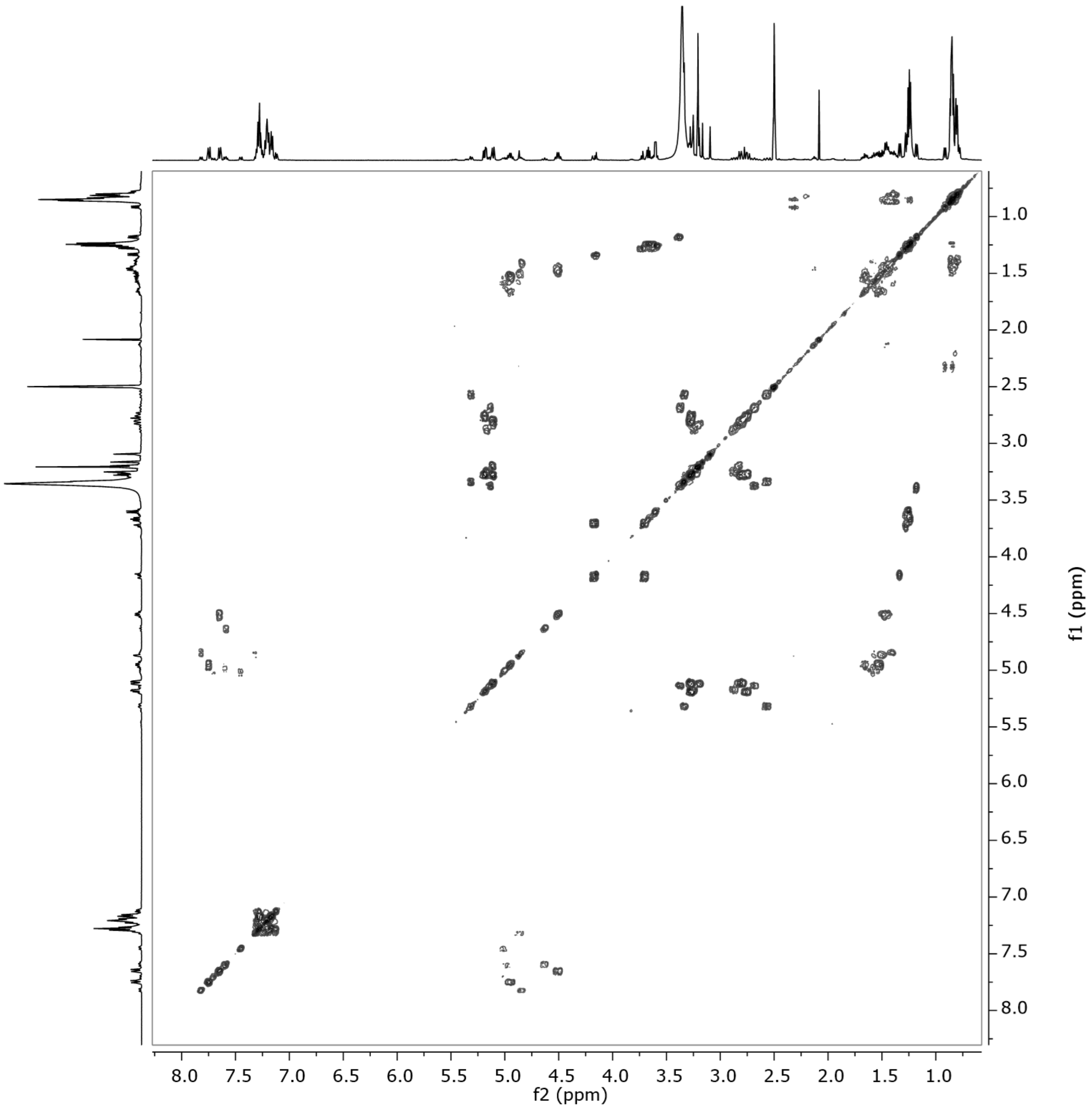

Figure S36. ^{1}H-^{1}H COSY spectrum of **5** in DMSO-d_6 at 500 MHz.

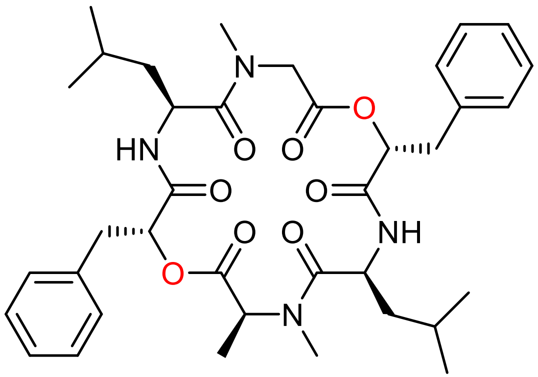

Cardinalisamide B (**5**)
Chemical Formula: $C_{37}H_{50}N_4O_8$
Exact Mass: 678.3629
Molecular Weight: 678.8270

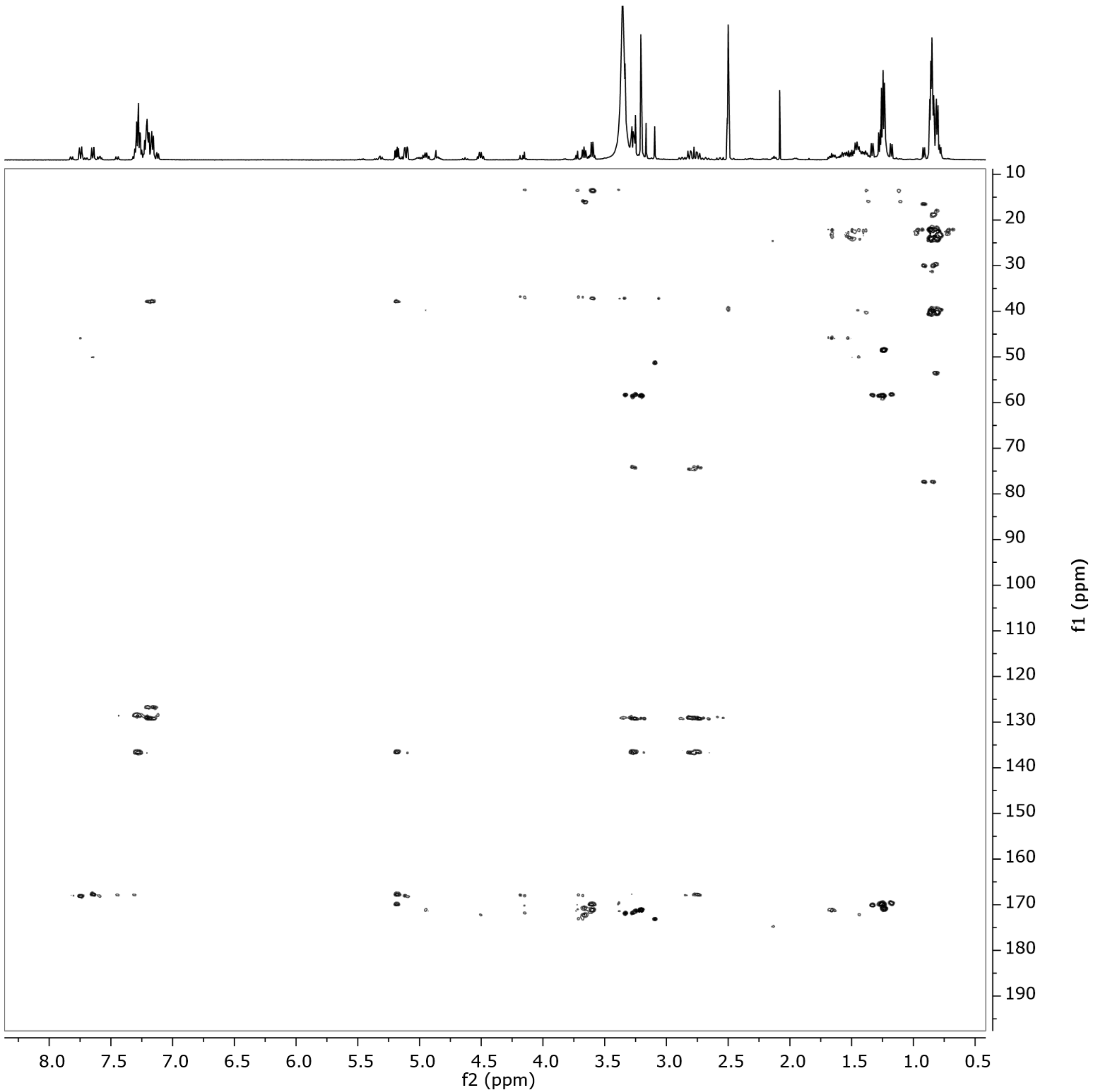

Figure S37. HMBC spectrum of **5** in DMSO-d_6 at 500 MHz.

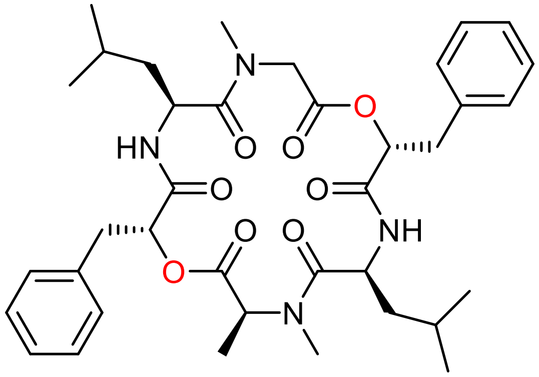

Cardinalisamide B (**5**)
Chemical Formula: $C_{37}H_{50}N_4O_8$
Exact Mass: 678.3629
Molecular Weight: 678.8270

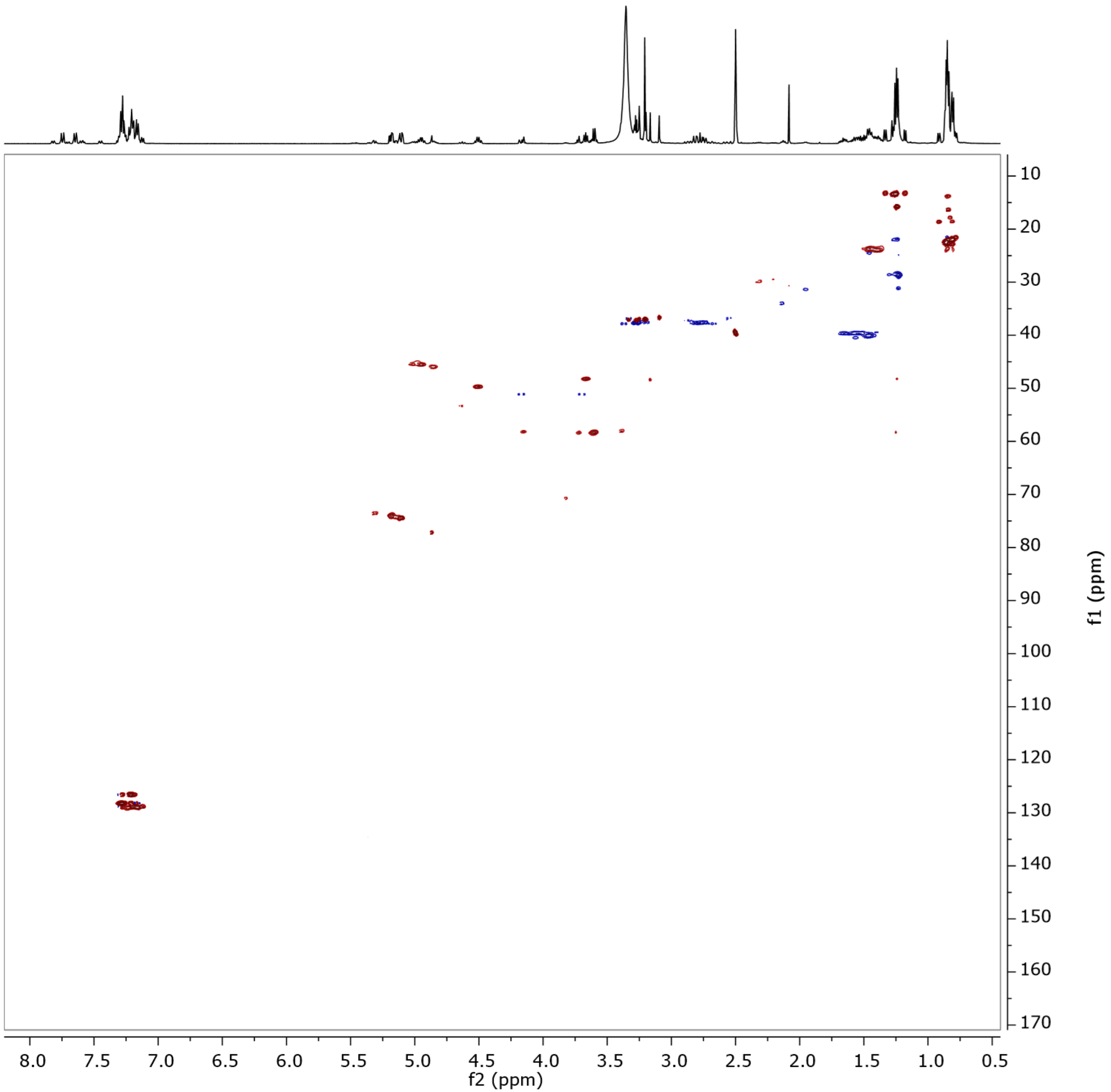

Figure S38. HSQC spectrum of **5** in DMSO-d_6 at 500 MHz.

Display Report

Analysis Info

		Acquisition Date	07.07.2023 08:45:27
Analysis Name	S:\DATA\AmaZon\gph22_Kunthide-Gift Phutthacharoen\07-23\MY 07796-F3 R1-F11_RD6_01_14793.d		
Method	14793.m	Operator	Lab
Sample Name	MY 07796-F3 R1-F11	Instrument	amaZon speed
Comment			

Acquisition Parameter

Ion Source Type	ESI	Ion Polarity	Negative	Alternating Ion Polarity	on
Mass Range Mode	UltraScan	Scan Begin	100 m/z	Scan End	2000 m/z
Accumulation Time	4000 µs	RF Level	100 %	Trap Drive	68.9
SPS Target Mass	1000 m/z	Averages	6 Spectra		

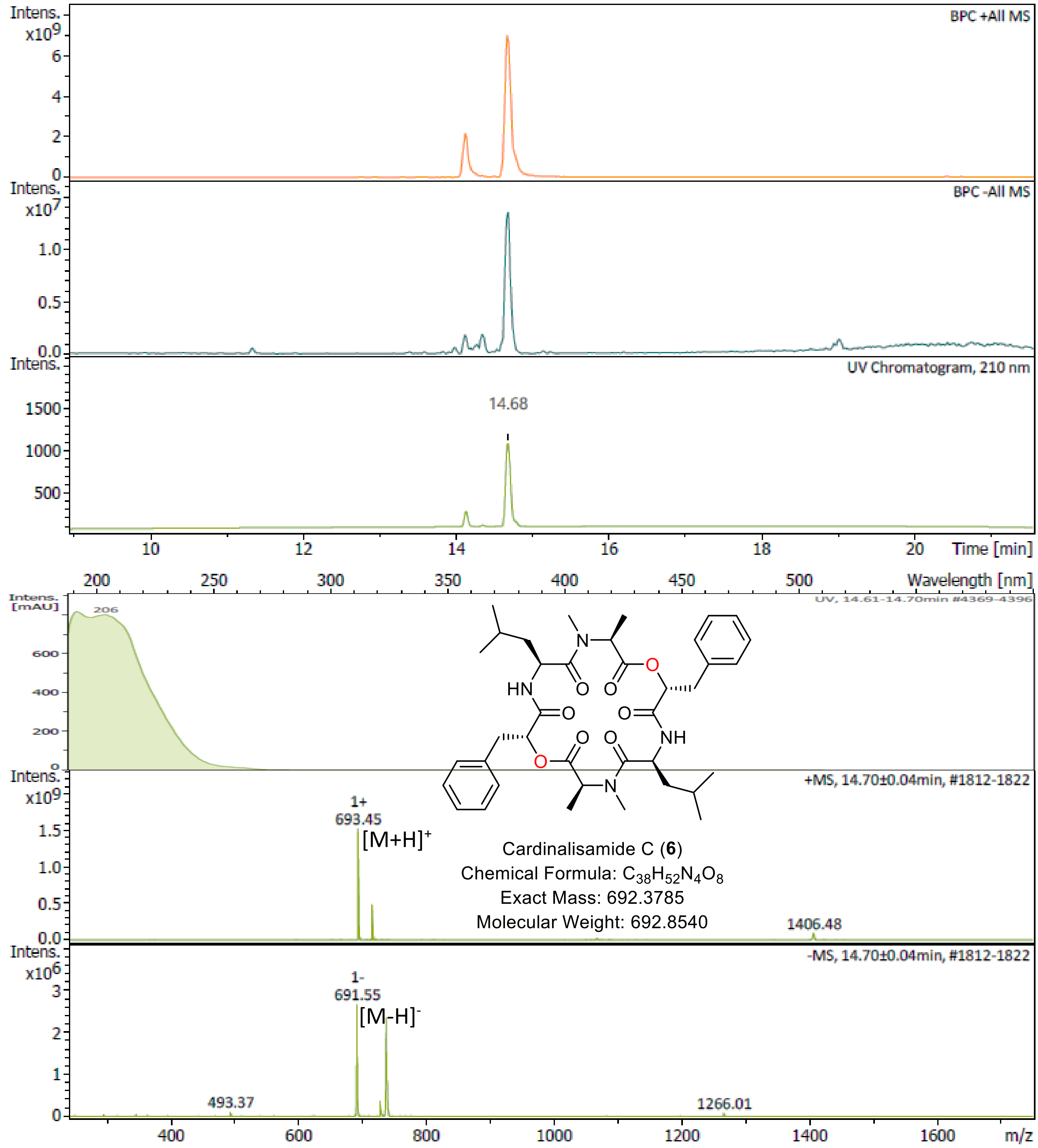

Bruker Compass DataAnalysis 6.1 printed: 27.03.2024 11:13:28 by: sel22 Page 1 of 1

Figure S39. LR-ESI-MS of **6**.

Display Report

Analysis Info

Analysis Name	S:\DATA\MaXis\GPH22_Gift_Kunthide_Phutthacharoen\23_07\MY07996-F3R1-F11_31_01_12258.d	Acquisition Date	10.07.2023 19:54:33
Method	pos_säure_10000_screening_ms_100_2500_line.m	Operator	ate06
Sample Name	MY07996-F3 R1-F11	Instrument	maXis
Comment	Screening01<br>Waters Acquity UPLC BEH C_18 1,7um 2.1x50mm		

Acquisition Parameter

Ion Polarity Positive

SPS Target Mass

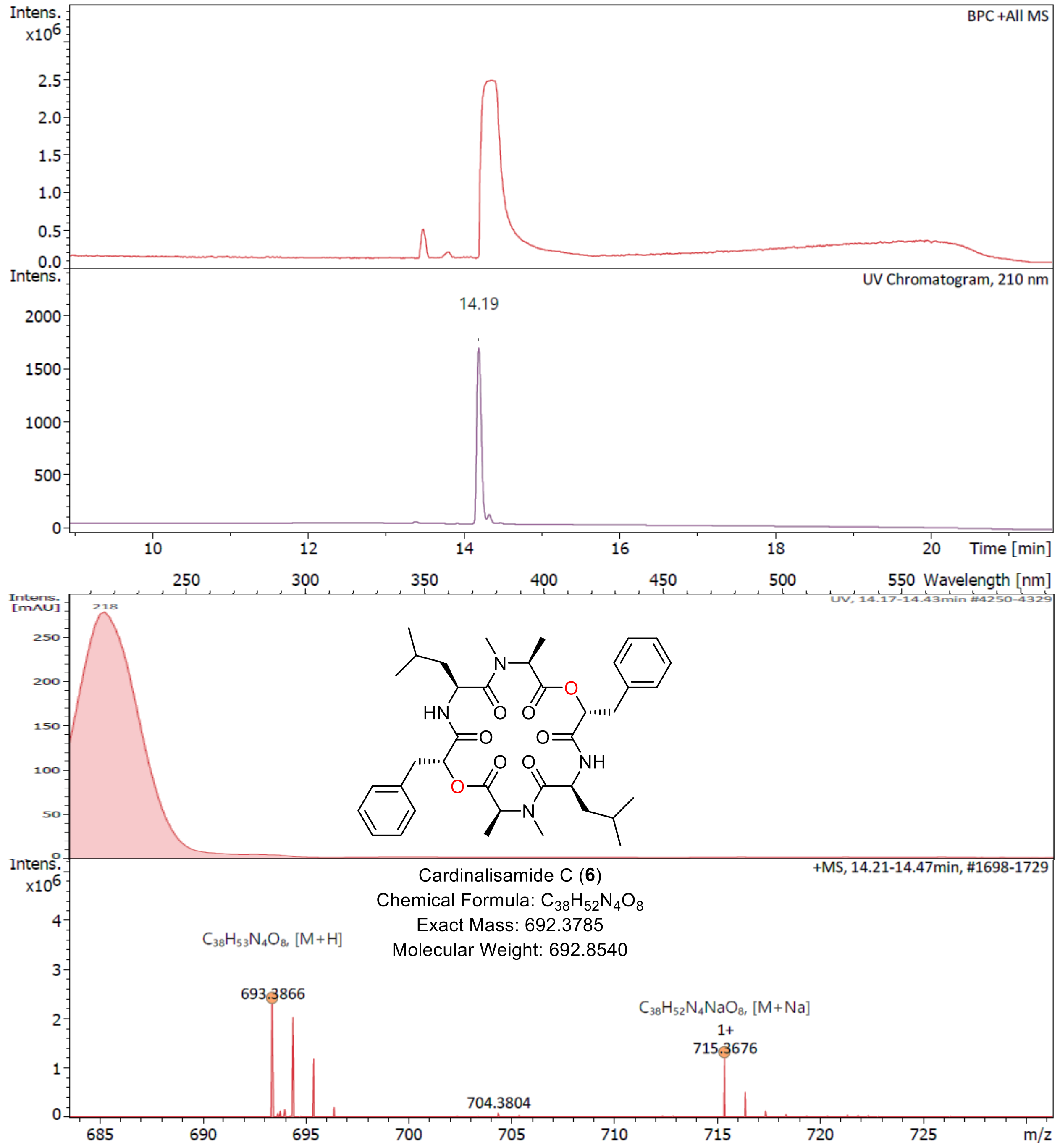

Bruker Compass DataAnalysis 6.1 printed: 27.03.2024 11:22:37 by: sel22 Page 1 of 1

Figure S40. HR-ESI-MS of **6**.

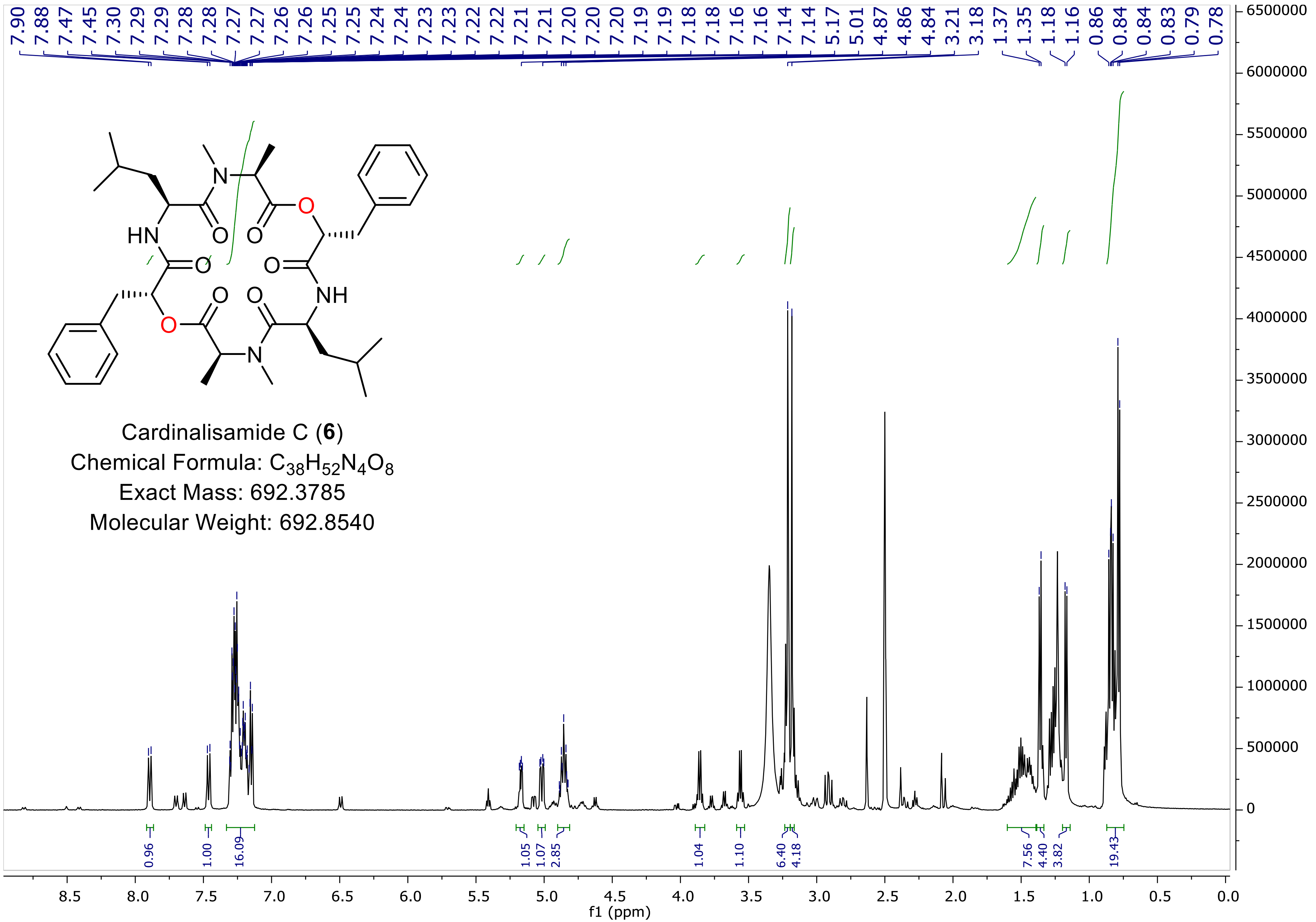

Figure S41. ^{1}H NMR spectrum of **6** in DMSO-d_6 at 500 MHz.

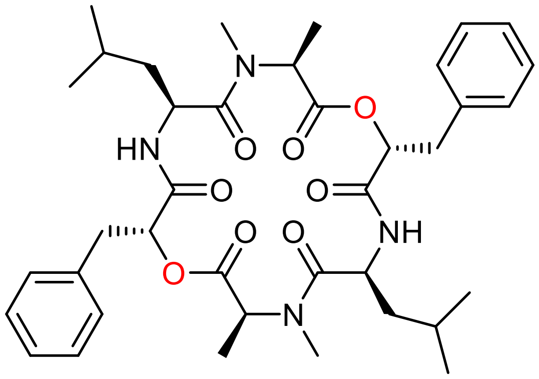

Cardinalisamide C (**6**)
Chemical Formula: $C_{38}H_{52}N_4O_8$
Exact Mass: 692.3785
Molecular Weight: 692.8540

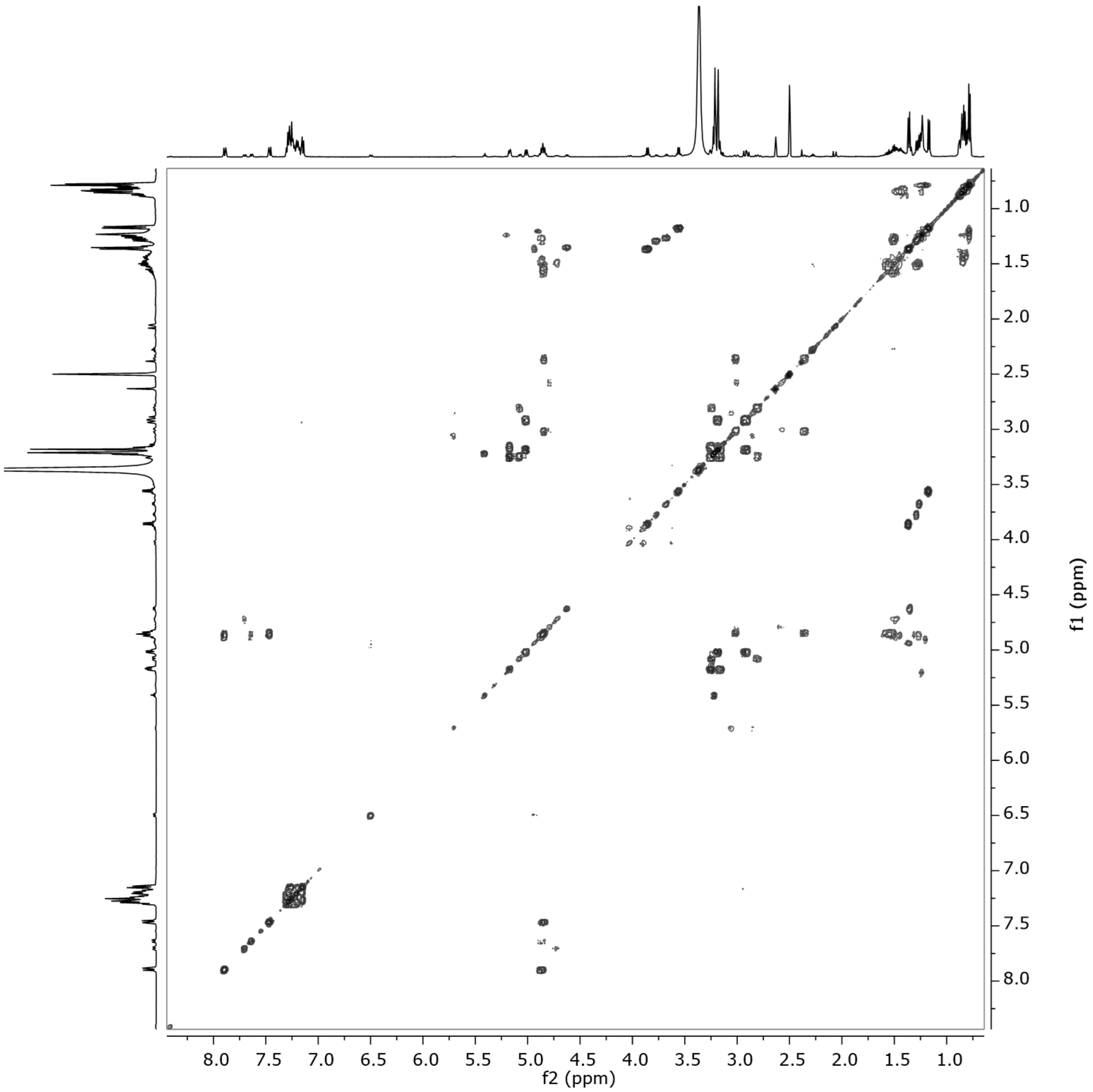

Figure S42. ^{1}H-^{1}H COSY spectrum of **6** in DMSO-d_6 at 500 MHz.

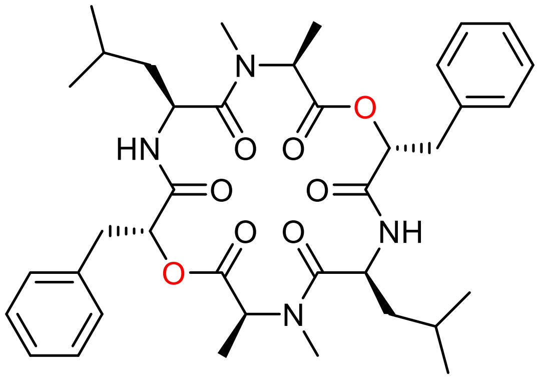

Cardinalisamide C (**6**)
Chemical Formula: $C_{38}H_{52}N_4O_8$
Exact Mass: 692.3785
Molecular Weight: 692.8540

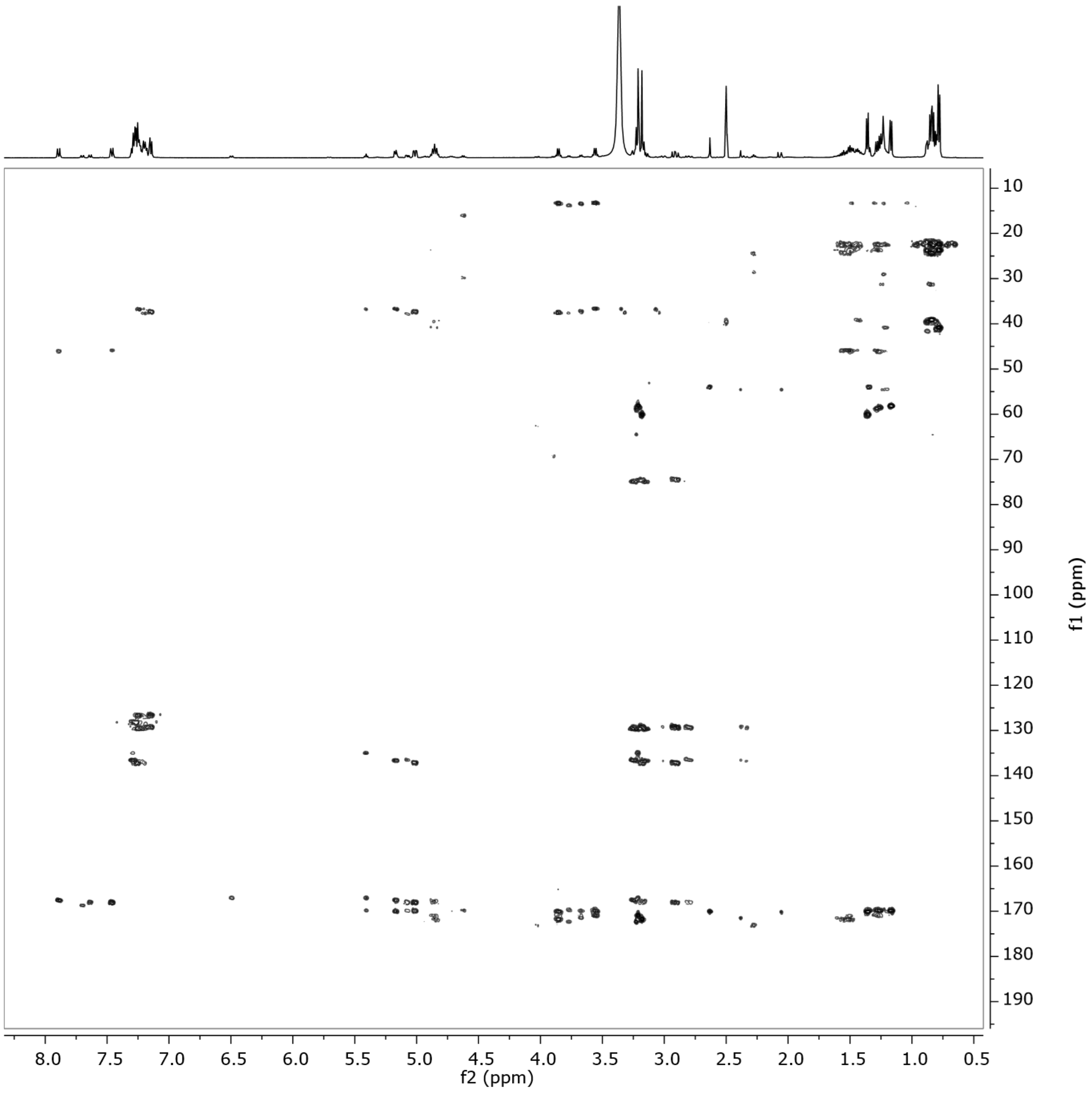

Figure S43. HMBC spectrum of **6** in DMSO-d_6 at 500 MHz.

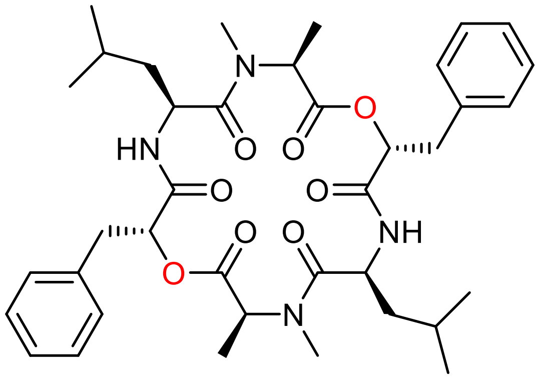

Cardinalisamide C (**6**)
Chemical Formula: $C_{38}H_{52}N_4O_8$
Exact Mass: 692.3785
Molecular Weight: 692.8540

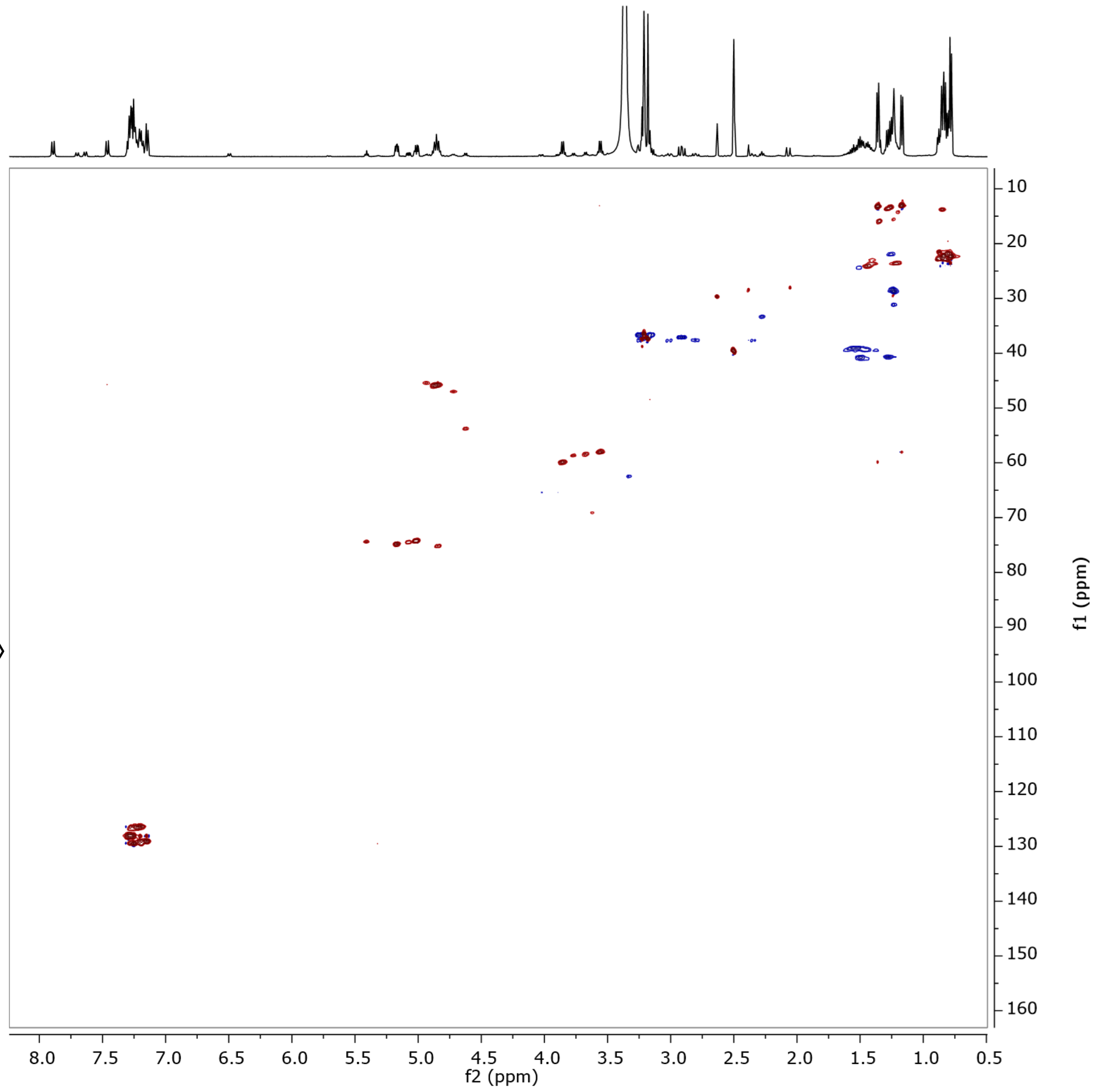

Figure S44. HSQC spectrum of **6** in DMSO-d_6 at 500 MHz.

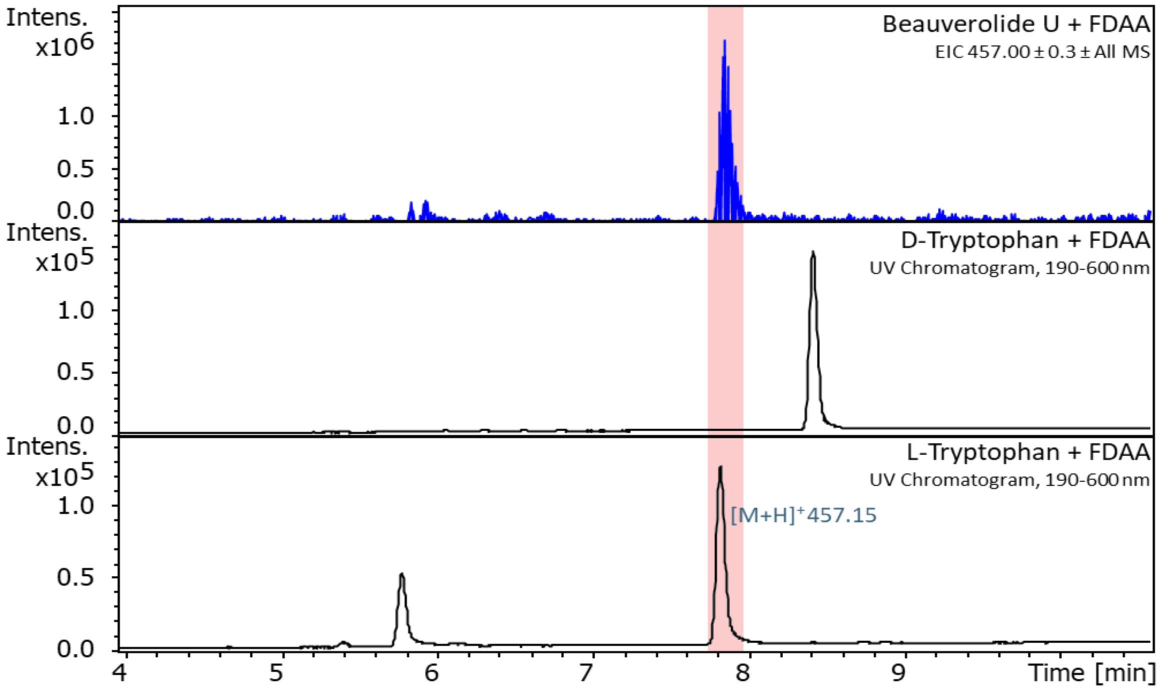

Figure S45. LC-ESI-MS spectra of beauverolide U (**3**), D-tryptophan and L-tryptophan with FDAA. Top-bottom: beauverolide U + FDAA, D-tryptophan + FDAA, L-tryptophan + FDAA. [M+H]$^+$ of the adduct is displayed in blue. Extracted ion chromatogram (*m/z* 457) and UV-chromatogram at 190-600 nm are shown. Bar indicates identical MS-Peaks (pink L-tryptophan).

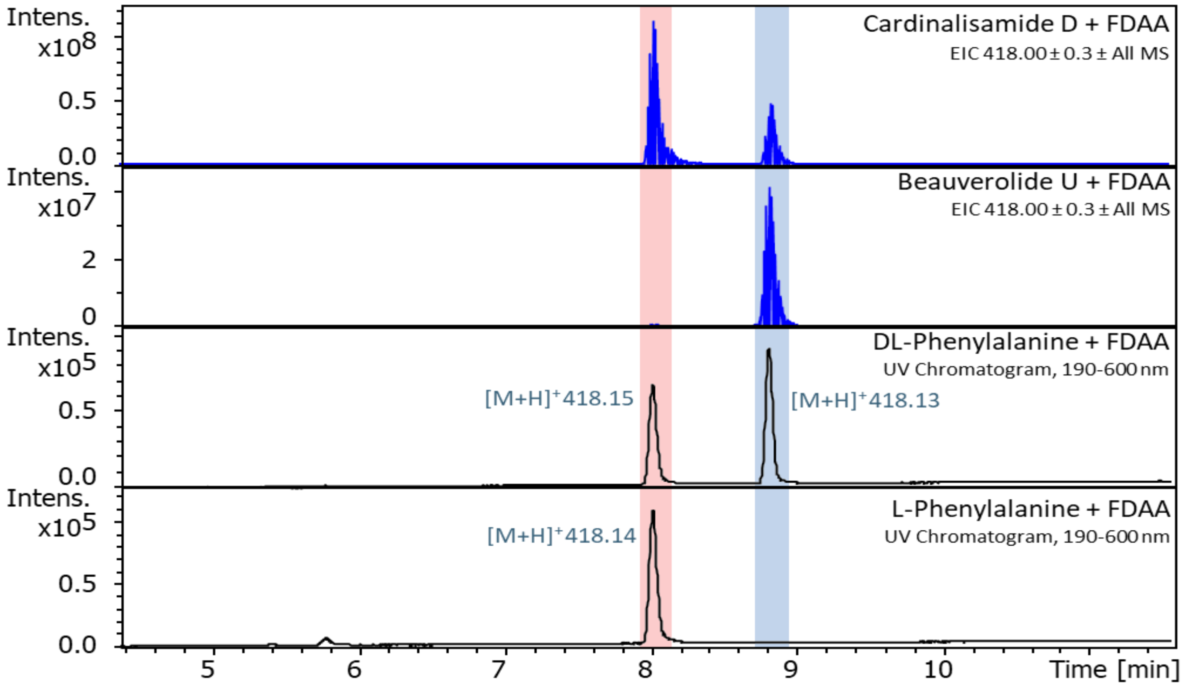

Figure S46. LC-ESI-MS spectra of cardinalisamide D (**4**), beauverolide U (**3**), DL-phenylalanine and L-phenylalanine with FDAA. Top-bottom: cardinalisamide D + FDAA, beauverolide U + FDAA, DL-phenylalanine + FDAA, L-phenylalanine + FDAA. [M+H]$^+$ of the adducts are displayed in blue. Extracted ion chromatogram (*m/z* 418) and UV-chromatogram at 190-600 nm are shown. Bars indicate identical MS-Peaks (pink L-phenylalanine and blue D-phenylalanine).

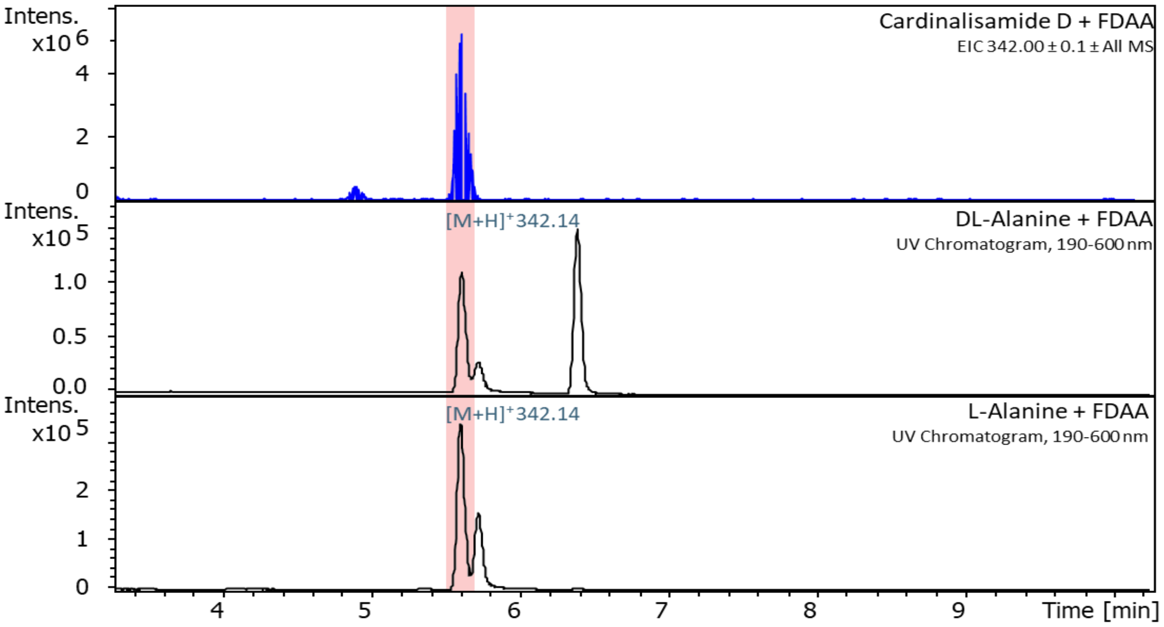

Figure S47. LC-ESI-MS spectra of cardinalisamide D (**4**), DL-alanine and L-alanine with FDAA. Top-bottom: cardinalisamide D (**4**) + FDAA, DL-alanine + FDAA, L-alanine + FDAA. $[M+H]^+$ of the adducts is displayed in blue. Extracted ion chromatogram (*m/z* 342) and UV-chromatogram at 190-600 nm are shown. Bar indicates identical MS-Peaks (pink L-alanine).

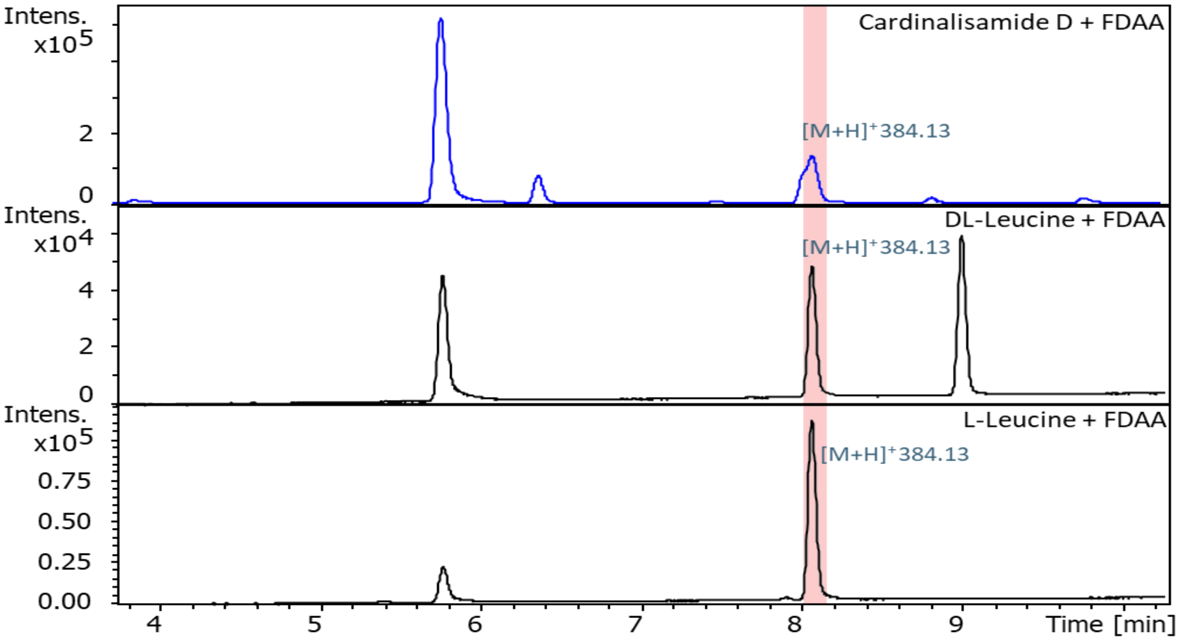

Figure S48. LC-ESI-MS spectra of cardinalisamide D (**4**), DL-leucine and L-leucine with FDAA. Top-bottom: cardinalisamide D (**4**) + FDAA, DL-leucine + FDAA, L-leucine + FDAA. $[M+H]^+$ of the adducts is displayed in blue. UV-chromatogram at 190-600 nm is shown. Bar indicates identical MS-Peaks (pink L-leucine).

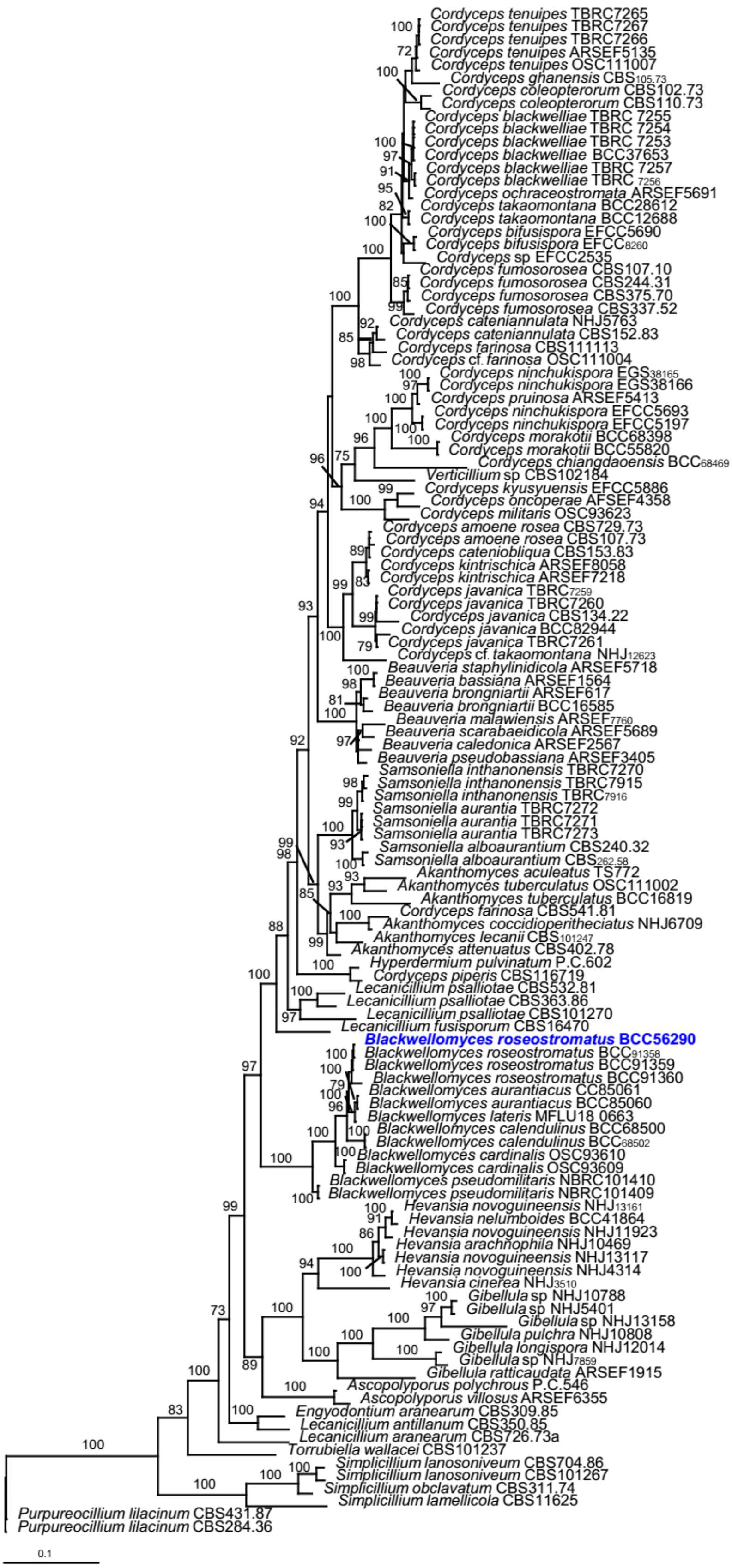

Figure S49. Maximum likelihood phylogenetic tree inferred from 117 taxa of Cordycipitaceae based on combined ITS, LSU, *EF1* and *RPB1* sequence data. MLBP values ≥70% are given above the nodes. Strain/culture numbers are given after the taxon names. The tree is rooted with *Purpureocillium lilacinum* CBS431.87 and CBS284.36. Newly generated sequence is in **blue**.

Table S1. Minimum inhibitory concentration (MIC) of compounds **1**–**6**.

	MIC (µg/mL)						
**Test Organism**	**1**	**2**	**3**	**4**	**5**	**6**	**Reference**
Schizosaccharomyces pombe (DSM 70572)	-	-	-	-	-	-	2.1 ^{N}
Pichia anomala (DSM 6766)	-	-	-	-	-	-	4.2 ^{N}
Mucor hiemalis (DSM 2656)	-	-	-	-	-	-	4.2 ^{N}
Candida albicans (DSM 1665)	-	-	-	-	-	-	2.1 ^{N}
Rhodotorula glutinis (DSM 10134)	-	-	-	-	-	-	1.0 ^{N}
Acinetobacter baumanii (DSM 30008)	-	-	-	-	-	-	0.53 ^{C}
Escherichia coli (DSM 1116)	-	-	-	-	-	-	0.83 ^{G}
Bacillus subtilis (DSM 10)	-	-	-	-	-	-	16.6 ^{O}
Mycobacterium smegmatis (ATCC 700084)	-	-	-	-	-	-	0.1 ^{K}
Staphylococcus aureus (DSM 346)	-	-	-	-	-	-	0.42 ^{G}
Pseudomonas aeruginosa (PA14)	-	-	-	-	-	-	0.42 ^{G}
Chromobacterium violaceum (DSM 30191)	-	-	-	-	-	-	1.7 ^{G}

(-): no inhibition observed, C: Ciprofloxacin lactate, G: Gentamycin, K: Kanamycin, N: Nystatin, O: Oxytetracyclin.

Table S2. Nematicidal activity of compounds **1**–**6**.

**Test Organism**	**Concentration (µg/mL)**	**Corrected mortality rate (%)**					
		**1**	**2**	**3**	**4**	**5**	**6**
***Caenorhabditis elegans* N21**	**100**	51.1 ± 5.3	36.1 ± 7.3	32.1 ± 7.0	23.4 ± 7.4	27.3 ± 8.5	53.6 ± 9.7
	**50**	31.7 ± 7.1	33.1 ± 6.7	6.0 ± 1.5	14.2 ± 4.9	17.9 ± 6.1	49.6 ± 4.5
	**10**	33.4 ± 5.3	11.6 ± 6.4	< 0	13.7 ± 5.2	29.2 ± 9.2	22.5 ± 5.5

< 0: less active than the corresponding negative control. Ivermectin (positive control): 87.4 ± 6.5 %, corrected mortality at 1 µg/mL.
